# *TRIM28* is a distinct prognostic biomarker that worsens the tumor immune microenvironment in lung adenocarcinoma

**DOI:** 10.18632/aging.103804

**Published:** 2020-10-22

**Authors:** Jie Liu, Xiao Han, Lijuan Chen, Dong Han, Xiaoqian Mu, Xiufeng Hu, Hongbo Wu, Huijuan Wu, Wenjing Liu, Yanqiu Zhao

**Affiliations:** 1 Department of Internal Medicine, The Affiliated Tumor Hospital of Zhengzhou University, Zhengzhou, China; 2 Department of Obstetrics and Gynecology, The Third Affiliated Hospital of Zhengzhou University, Zhengzhou, China; 3 Department of Oncology, The First Affiliated Hospital of Zhengzhou University, Zhengzhou, China

**Keywords:** TRIM28, tumor immune microenvironment, lung adenocarcinoma, prognosis

## Abstract

The tumor immune microenvironment (TIME) is an important determinant of cancer prognosis and treatment efficacy. To identify immune-related prognostic biomarkers of lung adenocarcinoma, we used the ESTIMATE algorithm to calculate the immune and stromal scores of 517 lung adenocarcinoma patients from The Cancer Genome Atlas (TCGA). We detected 985 differentially expressed genes (DEGs) between patients with high and low immune and stromal scores, and we analyzed their functions and protein-protein interactions. *TRIM28* was upregulated in lung adenocarcinoma patients with low immune and stromal scores, and was associated with a poor prognosis. The TISIDB and TIMER databases indicated that *TRIM28* expression correlated negatively with immune infiltration. We then explored genes that were co-expressed with *TRIM28* in TCGA, and investigated DEGs based on *TRIM28* expression in GSE43580 and GSE7670. The 429 common DEGs from these analyses were functionally analyzed. We also performed a Gene Set Enrichment Analysis using TCGA data, and predicted substrates of TRIM28 using UbiBrowser. The results indicated that TRIM28 may negatively regulate the TIME by increasing the SUMOylation of IRF5 and IRF8. Correlation analyses and validations in two lung adenocarcinoma cell lines (PC9 and H1299) confirmed these findings. Thus, TRIM28 may worsen the TIME and prognosis of lung adenocarcinoma.

## INTRODUCTION

Lung cancer is still the most common form of cancer and the leading cause of cancer death worldwide in both developing and developed regions [1]. Non-small-cell lung cancer (NSCLC) accounts for 85% of lung cancer cases, and can be classified as adenocarcinoma, squamous cell carcinoma or large cell carcinoma. Lung adenocarcinoma (LUAD) is one of the most common subtypes of NSCLC [2], and has been extensively studied in recent years due to the great success of molecular targeted therapy.

Immune evasion is acknowledged as a hallmark of tumors [3], and different immune cell types contribute to immune infiltration and immune evasion. Immunotherapies such as programmed cell death-1 (PD1) / programmed cell death ligand-1 (PD-L1) inhibitors have become standard-of-care treatment options for NSCLC patients. However, only a small subset (20-30%) of patients respond to such treatments [4–7]. At present, the prognosis of LUAD remains poor, and the overall five-year survival rate is < 15% due to local and distant recurrences [8].

Tumor-infiltrating lymphocytes and neutrophils are known to influence the prognosis of cancers and the efficacy of antitumor therapies [9, 10]. The level of infiltrating stromal and immune cells in tumor samples can be predicted with the ESTIMATE (Estimation of STromal and Immune cells in MAlignant Tumor tissues using Expression data) algorithm, which calculates immune and stromal scores based on unique gene signatures. Two main gene signatures are used: one based on 141 stroma-related genes that reflect the presence of stroma in tumor tissues, and the other based on 141 immune-related genes that represent the infiltration of immune cells into tumor tissues [11]. The current knowledge about the link between the tumor immune microenvironment (TIME) and LUAD is insufficient. Therefore, there is an urgent need to better understand tumor-immune interactions and identify more precise prognostic predictors and molecular biomarkers for lung cancer.

Tripartite motif-containing (TRIM) proteins, which include a structurally conserved RING-finger domain, one or two B-box zinc finger domains and a coiled-coil domain, are considered to be significant regulators of carcinogenesis [12]. TRIM28 (also known as KAP1, TIF1β or KRIP1), one of the 60 members of the TRIM family, is a small ubiquitin-like modifier (SUMO) E3 ligase and a fundamental component of several macromolecular complexes [13–15]. TRIM28 is a poorly understood transcriptional co-factor with pleiotropic biological activities, including inducing gene silencing, promoting cellular proliferation and differentiation, promoting neoplastic transformation, inhibiting apoptosis, facilitating DNA repair, and guarding genomic integrity [16]. TRIM28 also promotes T cell activation, T cell tolerance, and the expression of various interleukins and other proinflammatory molecules [17–25]. The upregulation of TRIM28 predicts a poor prognosis in patients with gastric cancer [26], ovarian cancer [27], breast cancer [12] and colorectal cancer [28]. Lei et al. [29] found that the upregulation of TRIM28 promoted the growth of NSCLC and was a potential predictor of metastasis and prognosis in early-stage NSCLC patients. However, Chen et al. [30] reported that TRIM28 exerted anti-proliferative activity in lung cancer by repressing E2F family members that are critical for cell proliferation. Due to these contradictory observations, the prognostic value of TRIM28 in lung cancer remains unclear.

In this study, we conducted a comprehensive analysis of immune cell infiltration and gene expression in the TIME of LUAD based on the ESTIMATE algorithm, and then correlated these data with clinical and prognostic features. The results revealed the significant prognostic value of *TRIM28* expression and a potential mechanism whereby TRIM28 alters the TIME in LUAD.

## RESULTS

### The correlations among the immune and stromal scores, clinical features and prognoses of LUAD patients

**Figure 1:**
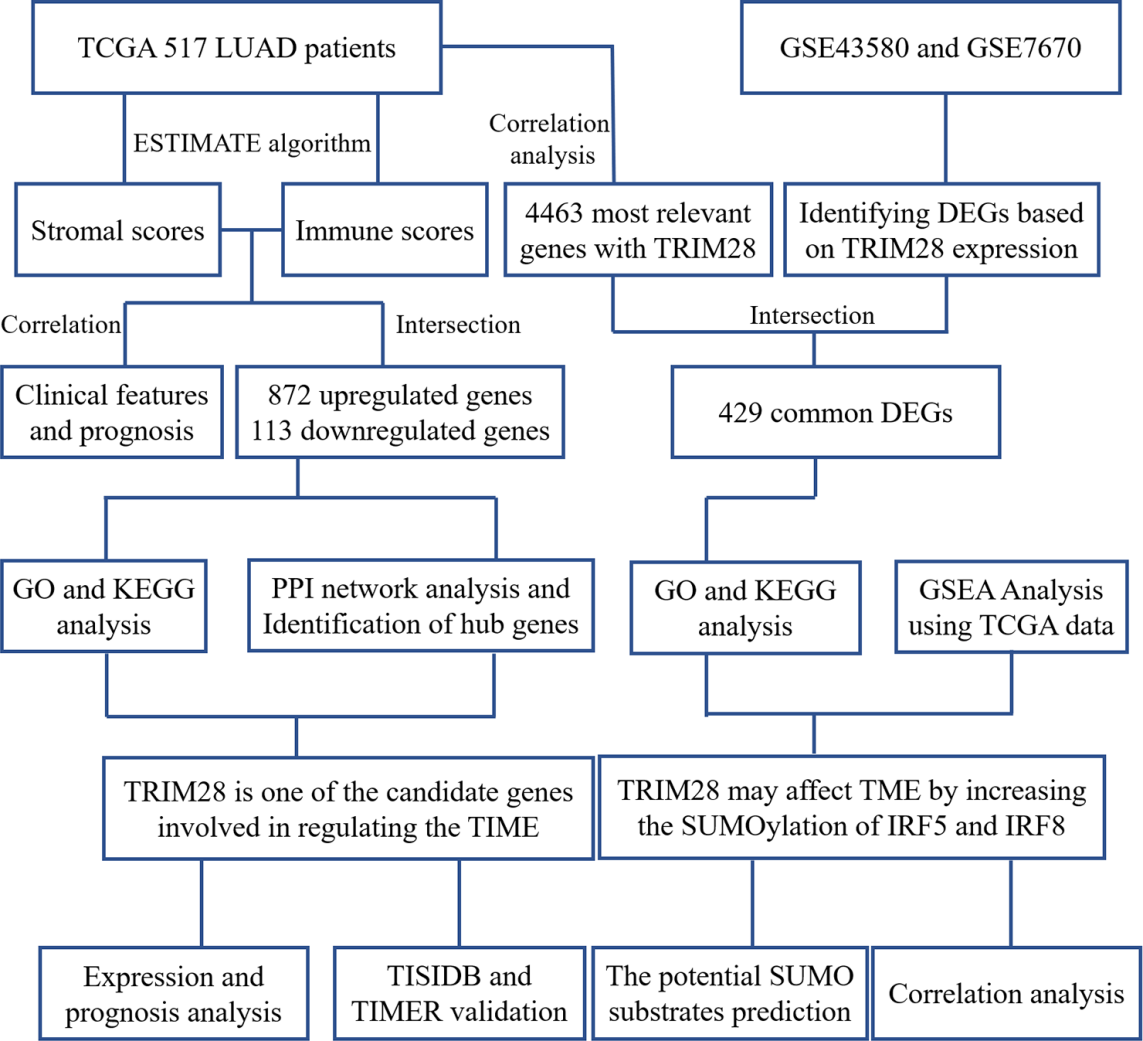
**Workflow of the present study.** TCGA, The Cancer Genome Atlas; LUAD, lung adenocarcinoma; PPI, protein-protein interaction; GSE, Gene Expression Omnibus data series; DEGs, differentially expressed genes; TIME, tumor immune microenvironment.

The overall flowchart of this study is shown in Figure 1. In total, 517 LUAD patients with RNA sequencing data and clinical information in The Cancer Genome Atlas (TCGA) database were included (http://www.cbioportal.org, Firehose Legacy, Supplementary Table 1) [31]. Patients’ immune and stromal scores were determined using the ESTIMATE algorithm based on gene expression data [11]. The detailed results are presented in Supplementary Table 2.

**Figure 2:**
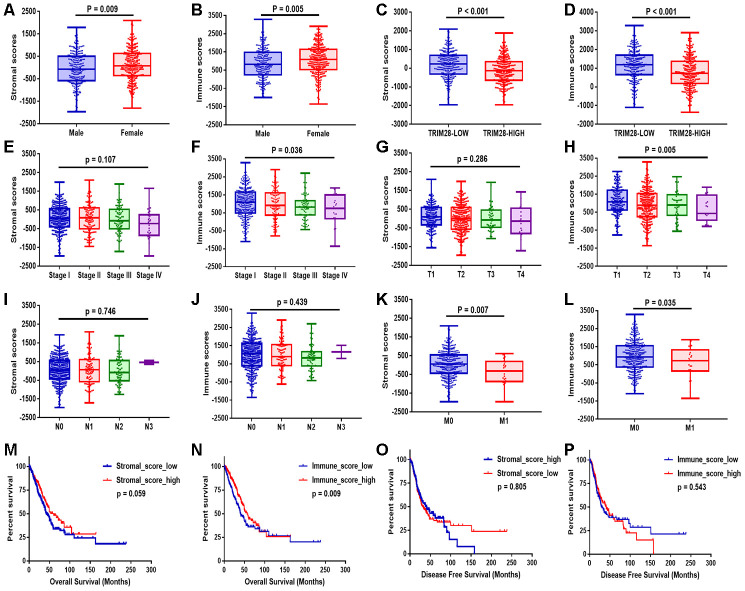
**Stromal and immune scores were associated with the clinical characteristics and OS of LUAD patients.** (**A**–**L**) The stromal and immune score distributions between patients with different genders (**A**, **B**), TRIM28 levels (**C**, **D**), TNM stages (**E**, **F**), T classifications (**G**, **H**), lymph node metastasis statuses (**I**, **J**) and distant metastasis statuses (**K**, **L**). (**M**–**P**) Patients were then divided into two groups according to the median stromal score or immune score. OS (**M**, **N**) and DFS (**O**, **P**) analyses were performed between the respective groups. OS, overall survival; LUAD, lung adenocarcinoma.

After comprehensively analyzing the stromal and immune scores, clinical information and RNA sequencing data, we found that both the stromal and immune scores were significantly lower in men (*p* = 0.009, *p* = 0.005, respectively; Figure 2A and 2B), in patients with higher *TRIM28* expression (*p* < 0.001, *p* < 0.001; Figure 2C and 2D) and in patients with metastasis (*p* = 0.007, *p* = 0.035; Figure 2K and 2L) than in their respective counterpart groups. In addition, the immune scores were lower in patients in higher tumor-node-metastasis (TNM) stages and T stages (*p* = 0.036, *p* = 0.005; Figure 2F and 2H). However, the stromal scores did not correlate with the TNM stages or T stages (*p* = 0.107, *p* = 0.286; Figure 2E and 2G). The stromal and immune scores also did not correlate significantly with the lymph node metastasis status (*p* = 0.746, *p* = 0.439; Figure 2I and 2J).

Patients were then divided into two groups according to the median value of the stromal score or the immune score. Then, overall survival (OS) (Figure 2M and 2N) and disease-free survival (DFS) (Figure 2O and 2P) were compared between the respective groups. OS was worse in patients with lower stromal scores or immune scores than in those with higher scores (*p* = 0.059, *p* = 0.009). However, DFS did not differ significantly between the respective groups.

### Identification and functional annotation of differentially expressed genes

**Figure 3:**
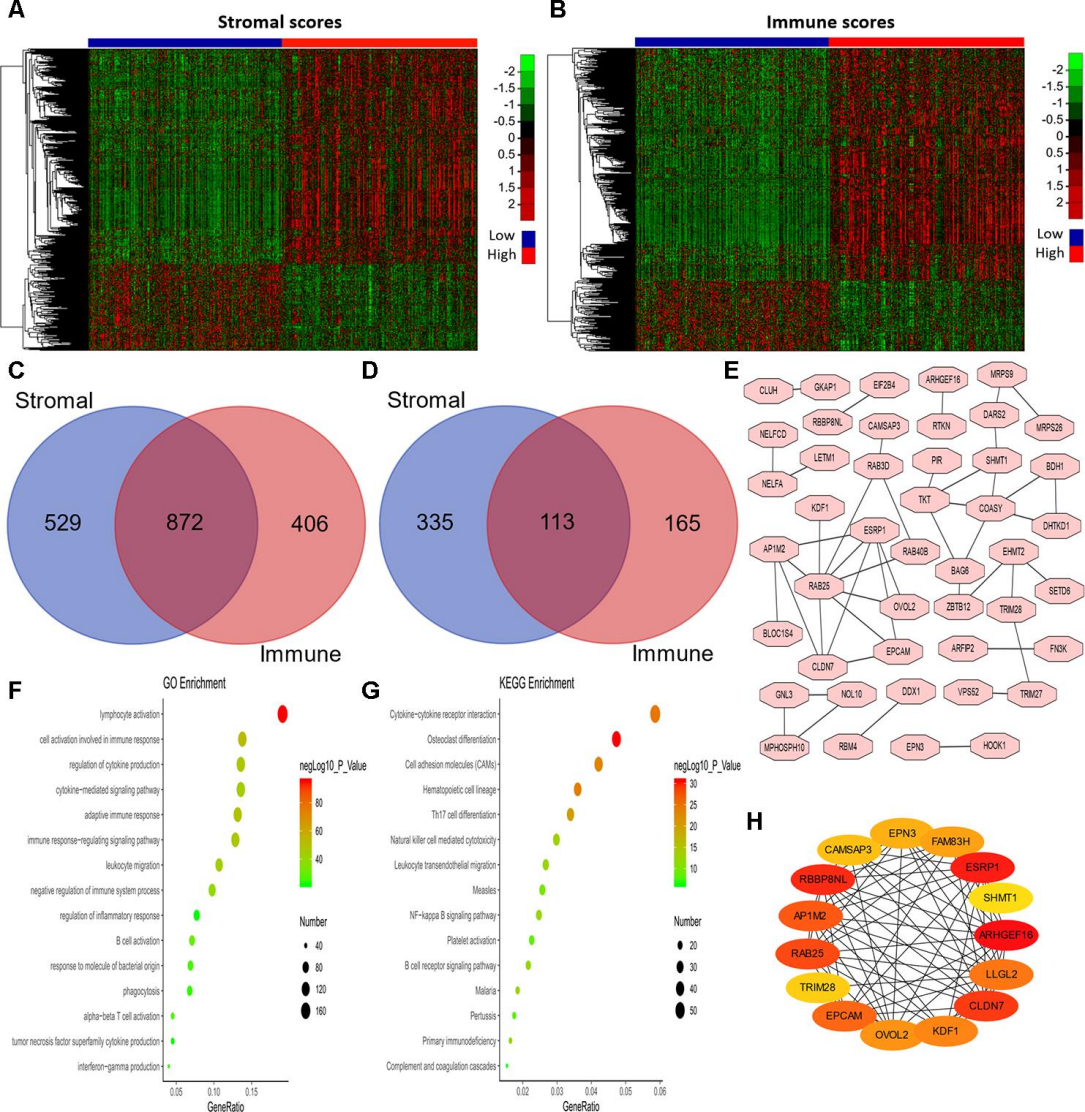
**Analysis of DEGs according to the immune and stromal scores in LUAD patients.** (**A**, **B**) Clustering Heatmap plot of the DEGs. The upper color bar represents the sample class; red represents the group with higher scores, while blue represents the group with lower scores. Genes with higher levels are shown in red, while those with lower levels are shown in green. (**C**, **D**) Venn diagrams showing the number of commonly upregulated (**C**) or downregulated (**D**) DEGs. (**E**) PPI analysis of downregulated DEGs via STRING. The interaction score was set to medium confidence (0.400). (**F**, **G**) The top 15 GO enrichment terms (**F**) and KEGG enrichment terms (**G**) for all DEGs, analyzed in Metascape. (**H**) The first 15 genes identified through the maximal clique centrality method were chosen as hub genes using the cytoHubba plugin. More red color represents more forward ranking. GO, gene ontology; KEGG, Kyoto Encyclopedia of Genes and Genomes; DEGs, differentially expressed genes; LUAD, lung adenocarcinoma; MMC, maximal clique centrality.

Next, we examined the differentially expressed genes (DEGs) between patients with high and low stromal scores, as shown in the heatmap in Figure 3A. We found that 1,401 genes were upregulated in the group with high stromal scores, while 448 genes were upregulated in the group with low stromal scores. We also evaluated the DEGs between patients with high and low immune scores (Figure 3B), and found that 1,278 genes were upregulated in the group with high immune scores, while 278 genes were upregulated in the group with low immune scores. Then, using an online tool (http://bioinformatics.psb.ugent.be/webtools/Venn/), we generated Venn diagrams to identify overlapping DEGs (Figure 3C and 3D). The results indicated that 872 genes were commonly upregulated in the groups with high stromal scores and high immune scores, while 113 genes were commonly upregulated in the groups with low stromal scores and low immune scores.

To explore the biological functions of the 985 DEGs, we performed Gene Ontology (GO) and Kyoto Encyclopedia of Genes and Genomes (KEGG) analyses using Metascape [32]. The top 15 GO enrichment terms and KEGG enrichment terms are shown in Figure 3F and 3G, respectively. Most of the terms were related to immune regulation, including lymphocyte activation, regulation of cytokine production, interferon production, etc.

Considering the poor prognoses of patients with low stromal or immune scores, we then performed a protein-protein interaction (PPI) analysis on the 113 genes that were commonly upregulated in patients with low stromal and immune scores (Figure 3E). The top 15 genes identified using the maximal clique centrality method were chosen as hub genes through the cytoHubba plugin: *ARHGEF16*, *ESRP1*, *TRIM28*, *RBBP8NL*, *CLDN7*, *RAB25*, *AP1M2*, *EPCAM*, *LLGL2*, *KDF1*, *OVOL2*, *FAM83H*, *EPN3*, *CAMSAP3* and *SHMT1* (Figure 3H). Interestingly, other methods in the cytoHubba plugin also identified *TRIM28* as a crucial hub gene (Supplementary Table 3).

### The mRNA and protein levels of TRIM28 across cancer types

**Figure 4:**
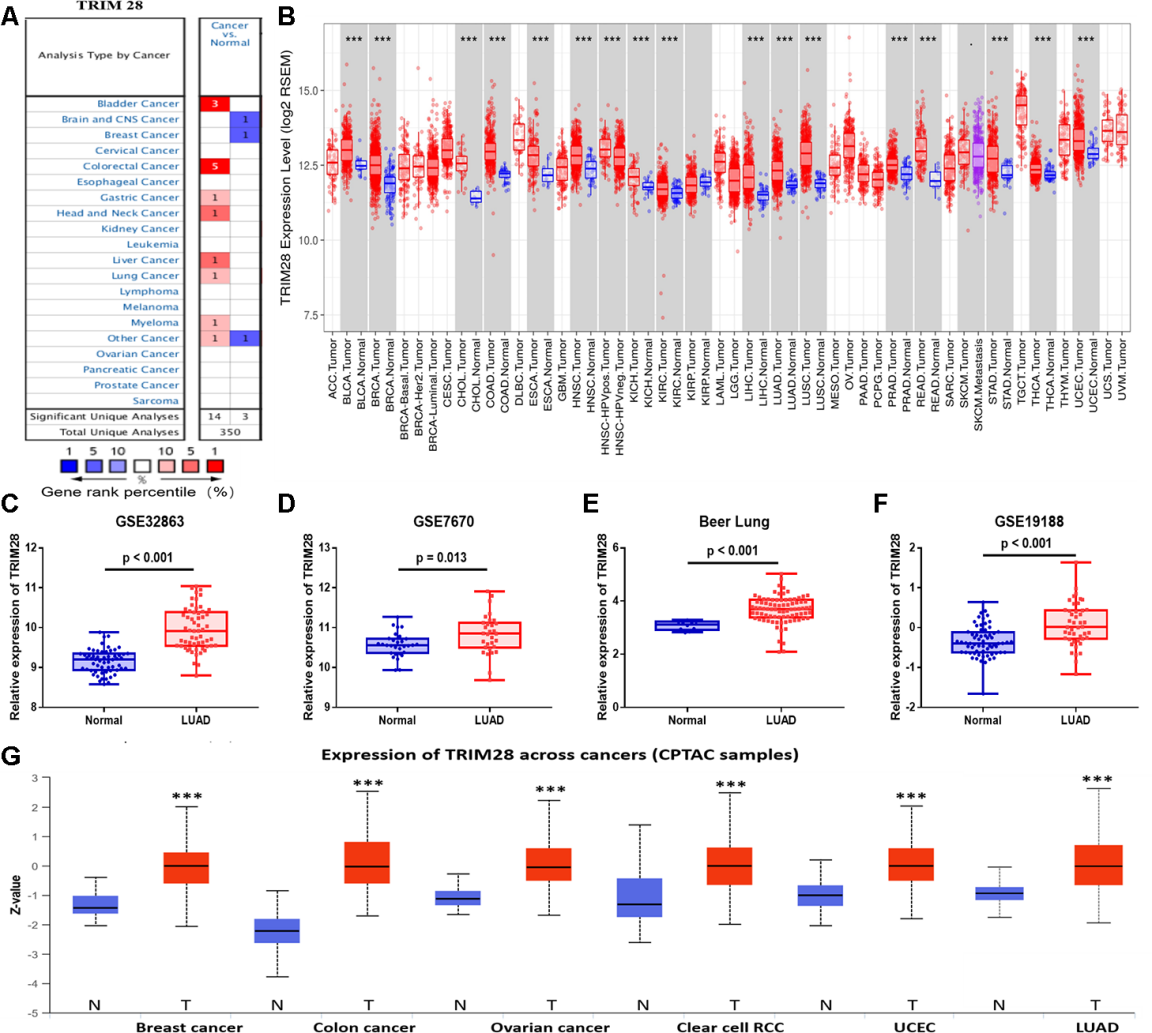
**TRIM28 levels in different human cancer types.** (**A**) Increased or reduced TRIM28 levels in different cancers compared with normal tissues in the Oncomine database. (**B**) Human TRIM28 levels in different tumor types from TCGA were determined using TIMER. (**C**–**F**) The significant increase in TRIM28 expression in LUAD was further validated in GSE32863 (**C**), GSE7670 (**D**), the Beer Lung dataset (**E**) and GSE19188 (**F**). (**G**) The protein expression of TRIM28 in various cancer tissues was detected using the UALCAN cancer database (*p < 0.05, **p < 0.01, ***p < 0.001). TCGA, The Cancer Genome Atlas; LUAD, lung adenocarcinoma; UCEC, uterine corpus endometrial carcinoma; GSE, Gene Expression Omnibus data series.

To determine whether *TRIM28* expression differed between tumor tissues and healthy tissues, we used the Oncomine database to analyze *TRIM28* mRNA levels in multiple cancer types. *TRIM28* expression was higher in bladder cancer, colorectal cancer, gastric cancer, head and neck cancer, liver cancer, lung cancer and multiple myeloma than in healthy tissues (Figure 4A). We also examined *TRIM28* RNA levels in various tumor tissues and adjacent healthy tissues using the RNA sequencing data in TCGA (Figure 4B). *TRIM28* was significantly upregulated in most of the tumor tissues, including bladder urothelial carcinoma, breast invasive carcinoma, LUAD, lung squamous cell carcinoma, etc. However, *TRIM28* RNA levels were significantly lower in kidney renal papillary cell carcinoma than in adjacent healthy tissues. The significant increase in *TRIM28* expression in LUAD was further validated in four independent data sets, including GSE32863 [33], GSE7670 [34], GSE19188 [35] and the Beer Lund dataset [36] (Figure 4C–4F).

We also used the UALCAN cancer database to examine TRIM28 protein levels in various cancer tissues. TRIM28 protein expression was upregulated in breast cancer, colon cancer, ovarian cancer, clear cell renal cell carcinoma, uterine corpus endometrial carcinoma and LUAD (Figure 4G).

### Prognostic value of *TRIM28* across cancer types

**Figure 5:**
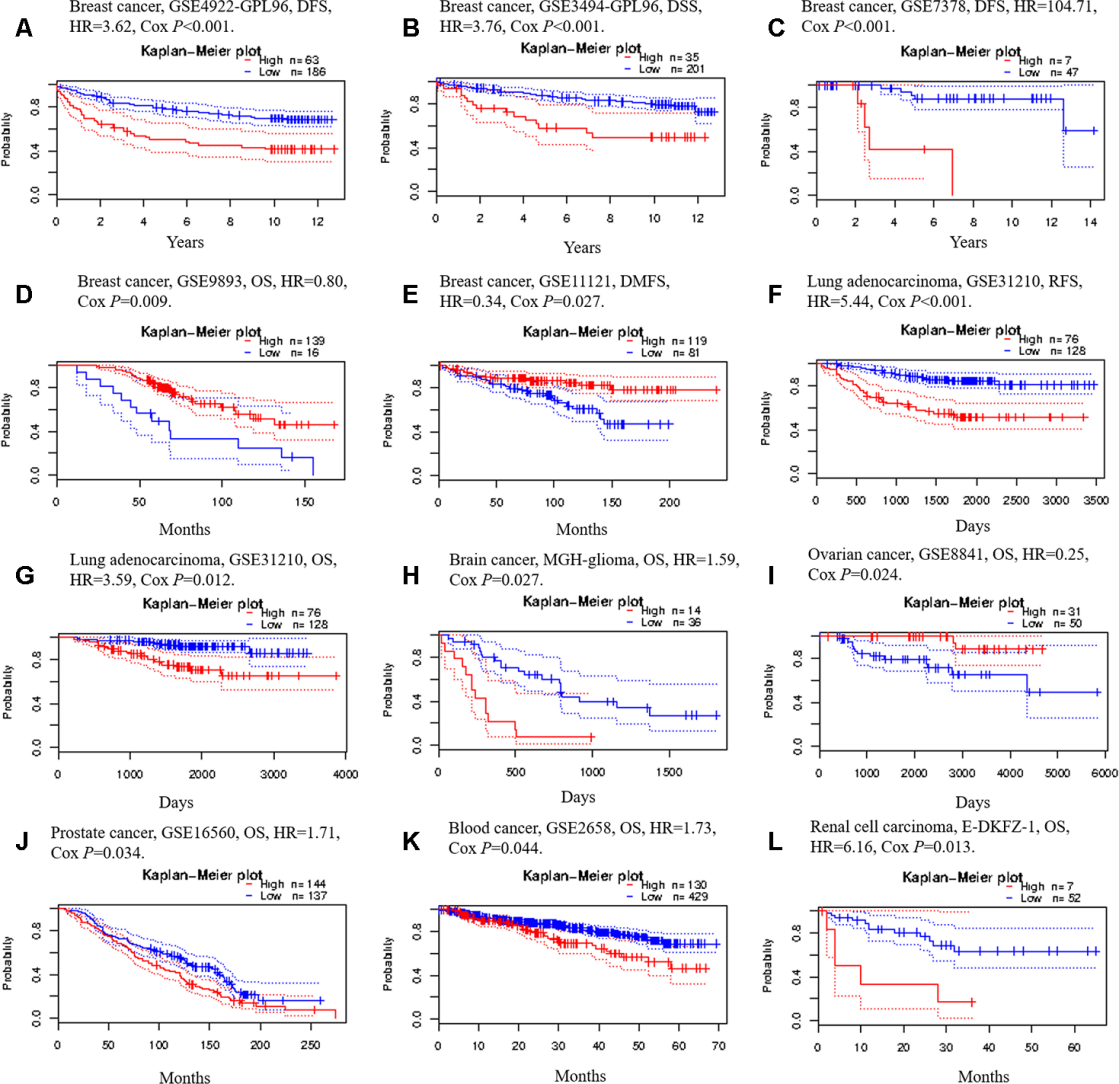
**Kaplan-Meier survival curves generated from the PrognoScan database for TRIM28 expression in different tumor types.** (**A**–**E**) DFS, DSS, DMFS and OS curves for five breast cancer cohorts (GSE4922-GPL96, GSE3494-GPL96, GSE7378, GSE9893 and GSE11121). (**F**, **G**) RFS and OS curves for lung cancer (GSE31210). (**H**) OS curve for brain cancer (MGH-glioma). (**I**) OS curve for ovarian cancer (GSE8841). (**J**) OS curve for prostate cancer (GSE16560). (**K**) OS curve for blood cancer (GSE2658). (**L**) OS curve for renal cell carcinoma. DFS, disease-free survival; DSS, disease-specific survival; DMFS, distant metastasis-free survival; OS, overall survival; RFS, recurrence-free survival; GSE, Gene Expression Omnibus data series; HR, hazard ratio.

We then used the PrognoScan database to investigate whether *TRIM28* expression correlated with the prognosis of cancer patients. Notably, *TRIM28* expression significantly impacted the prognosis of seven cancer types, including breast, lung, ovarian, brain, skin, prostate and blood cancers (Figure 5A–5L). In three cohorts (GSE4922-GPL96, GSE3494-GPL96 and GSE7378) [37–39] that respectively included 249 samples, 236 samples and 54 cases at different stages of breast cancer, higher *TRIM28* expression was marginally associated with poorer DFS or disease-specific survival (DSS) (DFS hazard ratio [HR] = 3.62, 95% confidence interval [CI] = 2.03 to 6.44, Cox *p* < 0.001; DSS HR = 3.76, 95% CI = 1.77 to 7.98, Cox *p* < 0.001; DFS HR = 104.71, 95% CI = 6.95 to 1577.65, Cox *p* < 0.001; Respectively; Figure 5A–5C). However, in two other cohorts (GSE9893 and GSE11121) [40, 41] that respectively included 155 and 200 samples at different stages of breast cancer, lower *TRIM28* expression was associated with poorer OS or distant metastasis-free survival (DMFS) (OS HR = 0.80, 95% CI = 0.68 to 0.95, Cox *p* = 0.008; DMFS HR = 0.34, 95% CI = 0.13 to 0.89, Cox *p* = 0.028; Respectively; Figure 5D, 5E). In one cohort (GSE31210) [42] that included 204 samples at different stages of LUAD, higher *TRIM28* expression was marginally associated with poorer recurrence-free survival (RFS) and OS (RFS HR = 5.44, 95% CI = 2.73 to 10.87, Cox *p* < 0.001; OS HR = 3.59, 95% CI = 1.33 to 9.68, Cox *p* = 0.012; Figure 5F and 5G). We also observed the poor prognostic value of *TRIM28* in brain cancer, prostate cancer, blood cancer and renal cell carcinoma (Figure 5H, 5J–5L) and its good prognostic value in ovarian cancer (Figure 5I). These results suggested that *TRIM28* expression influences the prognosis of LUAD and other tumor types.

To further examine the prognostic potential of *TRIM28* in different cancers based on Affymetrix microarrays, we used the Kaplan-Meier plotter database. Higher *TRIM28* expression was associated with a poorer prognosis in lung cancer and gastric cancer. However, *TRIM28* expression had less of an impact on the prognosis of ovarian cancer and breast cancer (Supplementary Figure 1). These results confirmed the significant prognostic value of *TRIM28* expression in lung cancer and gastric cancer. We also analyzed the prognostic potential of *TRIM28* in 33 different cancer types by using Gene Expression Profiling Interactive Analysis (GEPIA) to evaluate RNA sequencing data from TCGA. Higher *TRIM28* levels were associated with poorer OS in adrenocortical carcinoma, brain lower grade glioma, LUAD, mesothelioma, skin cutaneous melanoma, etc. (Supplementary Figure 2). Thus, although the prognostic value of *TRIM28* expression differed among different cancer types, the results from different databases all confirmed the prognostic value of *TRIM28* expression in LUAD.

Next, we explored the relationship between *TRIM28* expression and the clinical characteristics of lung cancer patients in the Kaplan-Meier plotter database. Overexpression of *TRIM28* was associated with worse OS and worse first progression (FP), regardless of gender and smoking history (*p* < 0.001). Interestingly, when patients were analyzed according to the type of lung cancer, the upregulation of *TRIM28* was associated with worse OS and FP in LUAD (OS HR = 2.65, *p* < 0.001; FP HR = 2.00, *p* < 0.001), but was not associated with OS in lung squamous cell carcinoma (OS HR = 1.25, *p* = 0.095). Moreover, higher *TRIM28* expression was associated with worse OS in stage 1, stage 2, stage N0 and stage M0, but was not associated with worse OS according to the grade, stage 3, stage T, stage N1 or stage N2 (Table 1). These results indicated that *TRIM28* expression had more significant prognostic value in LUAD patients than in lung squamous cell carcinoma patients, and had more significant prognostic value in early-stage than in late-stage LUAD patients.

**Table 1:** Correlation between TRIM28 mRNA expression and prognosis in lung cancer patients with different clinicopathological characteristics, determined via Kaplan-Meier plotter.

**Clinicopathological characteristics**	**OS (n = 1928)**			**FP (n = 646)**		
	**N**	**Hazard ratio**	***P*-value**	**N**	**Hazard ratio**	***P*-value**
Sex						
Female	715	2.11(1.54-2.9)	<0.001	468	2.09(1.57-2.78)	<0.001
male	1387	1.48(1.25-1.75)	<0.001	514	1.87(1.44-2.44)	<0.001
Histology						
Adenocarcinoma	720	2.65(1.96-3.59)	<0.001	461	2.00(1.45-2.75)	<0.001
Squamous cell carcinoma	524	1.25(0.96-1.64)	0.095	141	2.15(1.27-3.64)	0.003
Grade						
I	201	0.81(0.56-1.16)	0.250	140	0.81(0.51-1.29)	0.380
II	310	1.34(0.96-1.87)	0.079	165	1.68(1.08-2.62)	0.020
III	77	0.64(0.28-1.47)	0.290	51	1.36(0.6-3.05)	0.460
Stage						
1	577	2.45(1.85-3.25)	<0.001	325	1.47(0.94-2.31)	0.091
2	244	2.2(1.45-3.35)	<0.001	130	0.79(0.46-1.35)	0.380
3	70	0.69(0.39-1.21)	0.200	19	-	-
4	4	-	-	0	-	-
Stage T						
1	437	1.37(0.97-1.92)	0.070	177	2.31(1.38-3.89)	0.001
2	589	1.26(0.99-1.6)	0.056	351	1.45(1.08-1.96)	0.014
3	81	0.8(0.47-1.34)	0.390	21	0.58(0.21-1.62)	0.290
4	46	1.46(0.72-2.96)	0.290	7	-	-
Stage N						
0	781	1.31(1.05-1.64)	0.015	374	1,83(1.3-2.56)	<0.001
1	252	1.29(0.94-1.76)	0.110	130	1.96(1.24-3.1)	0.004
2	111	1.18(0.78-1.78)	0.430	51	1.82(0.91-3.65)	0.087
Stage M						
0	681	1.61(1.3-1.99)	<0.001	195	1.56(0.94-2.59)	0.080
1	10	-	-	0	-	-
Smoking history						
Never smoked	205	4.1(2.17-7.73)	<0.001	193	2.72(1.66-4.45)	<0.001
Smoked	820	1.87(1.39-2.52)	<0.001	603	1.71(1.34-2.18)	<0.001

OS, overall survival; FP, first progression.

### *TRIM28* expression is associated with the immune infiltration level

**Figure 6:**
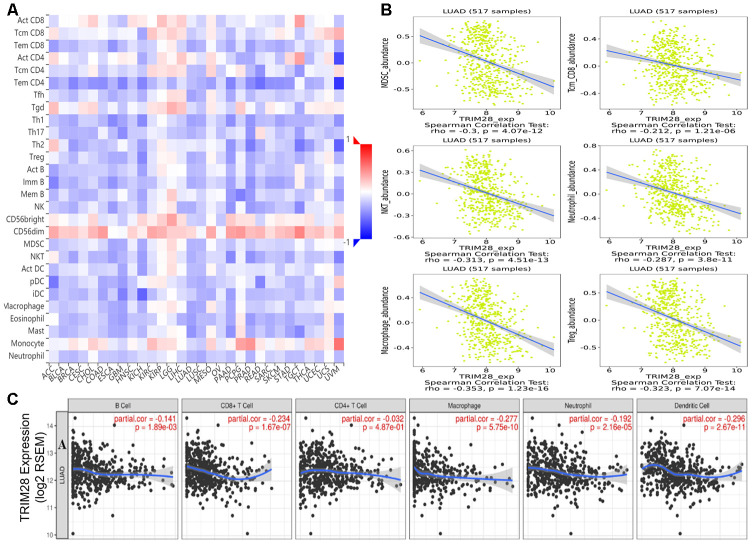
**Correlation of TRIM28 expression with immune cell infiltration.** (**A**) TRIM28 levels were significantly negatively associated with the levels of most tumor-infiltrating lymphocytes across human tumors in the TISIDB database. (**B**) TRIM28 levels correlated negatively with the levels of central memory CD8+ T cells, myeloid-derived suppressor cells, natural killer T cells, macrophages, neutrophils and regulatory T cells. (**C**) TRIM28 levels correlated negatively with the infiltrating levels of B cells, CD8+ T cells, macrophages, neutrophils and dendritic cells in LUAD in the TIMER database. LUAD, lung adenocarcinoma; Tcm_CD8, Central memory CD8 T cell; MDSC, Myeloid-derived suppressor cell; NKT, Natural killer T cell; Treg, regulatory T cell. TILs, tumor-infiltrating lymphocytes.

Numerous studies have demonstrated that the immune infiltrates in various human tumor types are associated with the prognosis and response to therapy [9, 10, 43]. We used the TISIDB and Tumor IMmune Estimation Resource (TIMER) databases to assess whether *TRIM28* expression was associated with the level of immune infiltration across human tumors. *TRIM28* levels correlated negatively with the levels of 28 types of tumor-infiltrating lymphocytes across human tumors in the TISIDB database (Figure 6A). *TRIM28* levels also correlated negatively with the levels of central memory CD8+ T cells (R = -0.212, *p* < 0.001), macrophages (R = -0.353, *p* < 0.001), natural killer T cells (R = -0.313; *p* < 0.001), myeloid-derived suppressor cells (R = -0.3, *p* < 0.001), regulatory T cells (R = -0.323, *p* < 0.001) and neutrophils (R = -0.287, *p* < 0.001) (Figure 6B). We then analyzed the relationship between *TRIM28* expression and immune infiltration in 39 tumor types in the TIMER database. *TRIM28* levels had strong negative associations with the infiltrating levels of CD8+ T cells in 24 tumor types, CD4+ T cells in 14 tumor types, macrophages in 25 tumor types, neutrophils in 27 tumor types and dendritic cells in 24 tumor types. In LUAD samples, *TRIM28* levels correlated negatively with the infiltrating levels of B cells (R = -0.141, *p* = 1.89e-03), CD8+ T cells (R = -0.234, *p* = 1.67e-07), macrophages (R = -0.277, *p* = 5.75e-10), neutrophils (R = -0.192, *p* = 2.16e-05) and dendritic cells (R = -0.296, *p* = 2.67e-11) (Figure 6C). These results strongly suggested that TRIM28 inhibits immune infiltration in LUAD.

We then explored the effects of *TRIM28* levels, clinicopathological characteristics and immune infiltration levels on survival using a multivariate Cox proportional hazards model. We found that age (*p* = 0.028), stage (*p* < 0.001), infiltrating B cell levels (*p* = 0.014) and *TRIM28* levels (*p* < 0.001) were independent predictors of survival in LUAD. However, only age (*p* = 0.024) and stage 3 (*p* = 0.011) were independent predictors of survival in lung squamous cell carcinoma (Table 2).

**Table 2:** A multivariate Cox proportional hazards model was used to explore the effects of TRIM28 expression, clinicopathological characteristics and immune infiltration levels on survival.

**LUAD (N=436)**	**Variable**	**Coef**	**HR**	**95%CI_l**	**95%CI_u**	***P*-value**
	Age	0.019	1.019	1.002	1.037	0.028
	Gender	-0.258	0.772	0.553	1.079	0.130
	Stage2	0.857	2.356	1.550	3.583	<0.001
	Stage3	1.065	2.901	1.900	4.430	<0.001
	Stage4	1.256	3.510	1.918	6.424	<0.001
	Purity	0.357	1.430	0.628	3.257	0.395
	B cell	-3.507	0.030	0.002	0.493	0.014
	CD8+Tcell	0.471	1.601	0.217	11.801	0.644
	CD4+Tcell	2.090	8.086	0.537	121.759	0.131
	Macrophage	0.782	2.187	0.138	34.629	0.579
	Neutrophil	-1.888	0.151	0.003	7.376	0.341
	Dendritic	0.219	1.245	0.297	5.208	0.765
	TRIM28	0.595	1.813	1.317	2.495	<0.001
LUSC (N=452)		Coef	HR	95%CI_l	95%CI_u	*P*-value
	Age	0.02	1.02	1.003	1.038	0.024
	Gender	0.303	1.353	0.957	1.913	0.087
	Stage2	0.107	1.113	0.792	1.564	0.539
	Stage3	0.495	1.641	1.121	2.402	0.011
	Stage4	0.982	2.67	0.949	7.507	0.063
	Purity	-0.128	0.88	0.422	1.834	0.733
	B cell	1.298	3.663	0.298	45.061	0.311
	CD8+Tcell	-1.727	0.178	0.028	1.124	0.066
	CD4+Tcell	0.705	2.023	0.153	26.753	0.593
	Macrophage	-0.339	0.713	0.061	8.326	0.787
	Neutrophil	0.980	2.665	0.099	71.525	0.559
	Dendritic	0.722	2.059	0.489	8.662	0.325
	TRIM28	0.000	1.000	0.774	1.292	1.000

Coef, regression coefficient; HR, hazard ratio; 95%CI_l, 95% confidence interval lower limit; 95%CI_u, 95% confidence interval upper limit.

### Analysis of genes co-expressed with *TRIM28* in LUAD

**Figure 7:**
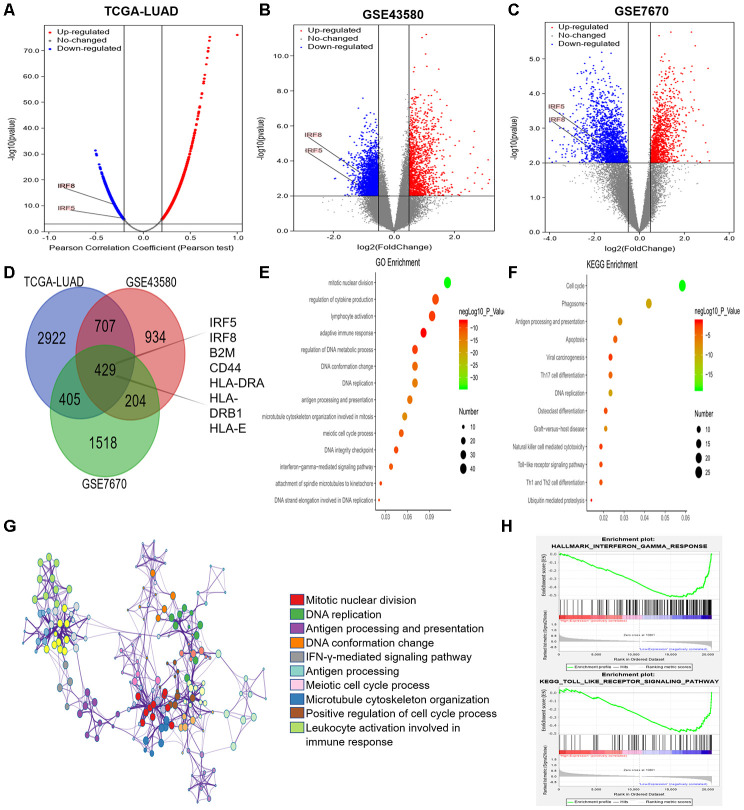
**Analysis of DEGs according to TRIM28 expression in LUAD patients.** (**A**) Volcano map showing all the genes associated with TRIM28 in LUAD. (**B**, **C**) Volcano maps showing all the DEGs based on TRIM28 expression. (**D**) Venn diagram showing the number of common DEGs. (**E**, **F**) Top 14 GO enrichment terms (**E**) and top 13 KEGG enrichment terms (**F**) for all DEGs, analyzed in Metascape. (**G**) Associations among the top 10 cluster enrichment terms analyzed by Metascape, displayed as a network. An edge links terms with a similarity score > 0.3. (**H**) Gene Set Enrichment Analysis according to the expression of TRIM28 in TCGA. GO, gene ontology; DEGs, differentially expressed genes; LUAD, lung adenocarcinoma; GSEA, Gene Set Enrichment Analysis; TCGA, The Cancer Genome Atlas; GSE, Gene Expression Omnibus data series.

Next, we used LinkedOmics [44] to identify genes that were co-expressed with *TRIM28* based on mRNA sequencing data from LUAD patients in TCGA. We generated a volcano map of all the genes associated with *TRIM28*, and found that interferon regulatory factor 5 (*IRF5*) and *IRF8* levels correlated negatively with *TRIM28* levels (Figure 7A). We also downloaded two mRNA expression datasets (GSE43580 and GSE7670) from the Gene Expression Omnibus (GEO) [34, 45]. We divided the samples in each dataset into two groups according to *TRIM28* expression, and we analyzed the DEGs between patients with higher and lower *TRIM28* levels. The volcano graphs in Figures 7B and 7C display the DEGs in GSE43580 and GSE7670, respectively. We then used a Venn diagram to evaluate the overlapping DEGs from Figure 7A–7C (Figure 7D). There were 429 common DEGs, including *IRF5*, *IRF8*, *B2M*, *CD44*, *HLA-DRA*, *HLA-DRB1* and *HLA-E*.

We then performed GO and KEGG analyses of these DEGs using Metascape [32]. We identified the top 14 GO enrichment terms (Figure 7E), which included the regulation of cytokine production and the interferon-gamma signaling pathway. The top 13 KEGG enrichment terms (Figure 7F) included the Toll-like receptor signaling pathway and ubiquitin-induced proteolysis. The correlations among the top 10 enriched terms from the GO analysis are shown as a network in Figure 7G. We also performed a Gene Set Enrichment Analysis using TCGA data, and found that the interferon-gamma and Toll-like receptor signaling pathways were enriched (Figure 7H). Combined with our previous results, these results may indicate that TRIM28 inhibits the interferon-gamma and Toll-like receptor signaling pathways by increasing the ubiquitination (degradation) of IRF5 and IRF8, ultimately suppressing immune infiltration.

### TRIM28 may worsen the TIME by increasing the SUMOylation of IRF5 and IRF8

**Figure 8:**
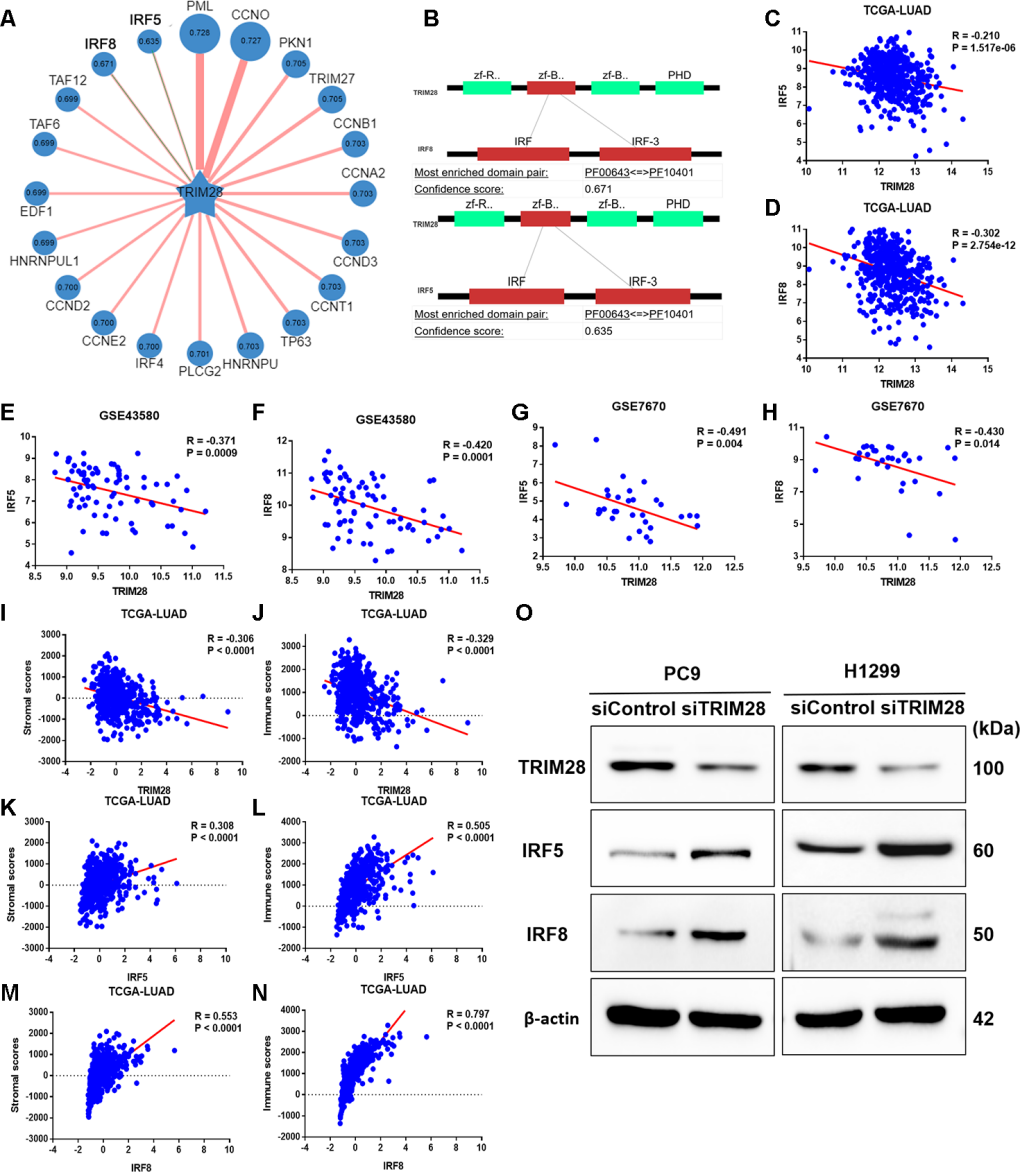
**The association of TRIM28 with IRF5 and IRF8.** (**A**) Network view of predicted E3-substrate interactions in UbiBrowser web services. In network view, the central node is the queried E3 ligase, and the surrounding nodes are the predicted substrates. The width of the edge reflects the confidence of the interaction. (**B**) The possible binding regions of IRF5 and IRF8 to TRIM28. (**C**–**H**) TRIM28 expression exhibited a significant negative correlation with IRF5 and IRF8 expression in TCGA (**C**, **D**), GSE43580 (**E**, **F**) and GSE7670 (**G**, **H**). (**I**–**N**) TRIM28 expression exhibited a strong negative correlation with stromal scores (**I**) and immune scores (**J**), while IRF5 and IRF8 levels exhibited strong positive relationships with stromal scores (**K**, **M**) and immune scores (**L**, **N**), respectively. (**O**) The expression of IRF5 and IRF8 after knocking down TRIM28 in two different LUAD cell lines (PC9 and H1299) through Western blotting. The gels have been run under the same experimental conditions. The blot of IRF5 in H1299 is obtained from the combined image merging the blot image and the ladder image. LUAD, lung adenocarcinoma; TCGA, The Cancer Genome Atlas; GSE, Gene Expression Omnibus data series.

To identify potential SUMO substrates of TRIM28, we queried TRIM28 as E3 in the web tool of UbiBrowser [46]. The 79 predicted substrates with middle-confidence interactions and 347 predicted substrates with low-confidence interactions are presented in Supplementary Table 4. Figure 8A displays some of the substrates, and Figure 8B displays the predicted binding regions of IRF5 and IRF8 to TRIM28. We then performed a correlation analysis, which indicated that *TRIM28* expression correlated negatively with *IRF5* and *IRF8* expression in TCGA (R = -0.210, *p* < 0.001; R = -0.302, *p* < 0.001; Figure 8C and 8D), GSE43580 (R = -0.371, *p* < 0.001; R = -0.420, *p* < 0.001; Figure 8E and 8F) and GSE7670 (R = -0.491, *p* = 0.004; R = -0.430, *p* = 0.014; Figure 8G and 8H), respectively. Moreover, *TRIM28* expression exhibited a strong negative correlation with stromal scores and immune scores (Figure 8I and 8J), while *IRF5* and *IRF8* expression exhibited strong positive correlations with stromal scores (Figure 8K and 8M) and immune scores (Figure 8L and 8N), respectively.

To verify our hypothesis that TRIM28 downregulates IRF5 and IRF8, we knocked down *TRIM28* in two different LUAD cell lines (PC9 and H1299). The knockdown efficiency was validated through Western blotting (Figure 8O). As expected, IRF5 and IRF8 levels increased significantly when *TRIM28* was knocked down (Figure 8O).

## DISCUSSION

Despite the latest advances in molecular targeted therapy and immunotherapy for LUAD, local and distant failures remain major therapeutic issues. In addition, immunotherapy is only effective in 20-30% of patients, and our knowledge of the TIME is inadequate. Therefore, we performed a comprehensive bioinformatics analysis to identify genes that may alter the TIME and prognosis of LUAD patients. We found that *TRIM28* levels correlated negatively with the stromal scores, immune scores and immune cell infiltration levels of LUAD patients. TRIM28 was previously reported as a cofactor that regulates the activity of various immune-related cells and the expression of multiple cytokines [17–25]. Our functional analyses of TRIM28 supported these conclusions.

Most of the published data suggest that TRIM28 exerts oncogenic effects, and microarray analyses in a wide variety of tumors have revealed that *TRIM28* mRNA levels are significantly greater in tumor tissues than in normal tissues [35, 36]. Our study also demonstrated that *TRIM28* mRNA levels were significantly greater in most tumor tissues than in adjacent healthy tissues, especially in the case of LUAD. However, we found some discrepancies in particular cancer types. For example, in breast cancer, three cohorts indicated that higher *TRIM28* levels were marginally associated with poorer DFS and DSS (Figure 5A–5C), but two other datasets demonstrated that lower *TRIM28* levels were associated with poorer DMFS and OS in the PrognoScan database (Figure 5D and 5E). In addition, *TRIM28* expression had little impact on breast cancer prognosis in the Kaplan-Meier plotter (Supplementary Figure 1) and no effect on breast cancer prognosis in GEPIA. These discrepancies may reflect differing data collection approaches or underlying mechanisms. However, in all the databases we examined, higher *TRIM28* expression was associated with a poorer prognosis of LUAD. These findings strongly suggested that *TRIM28* is a prognostic biomarker in LUAD.

The immune system is a critical regulator of tumor biology and has the capacity to support or inhibit tumor development, growth, invasion and metastasis. Tumor cells adopt a variety of mechanisms to avoid immune recognition and destruction, including: 1) downregulating human leukocyte antigen (HLA) class I molecules such as HLA-A, HLA-B, HLA-C and B2M on the cancer cell surface; 2) altering the antigen-presenting cell number or function; 3) lacking costimulation molecules such as B7-1, B7-2 and CD40; 4) promoting negative immune regulation by regulatory T cells and mesenchymal stem cells; 5) secreting immunosuppressive cytokines such as interleukin (IL)-10, transforming growth factor β and IL-6; 6) aberrantly expressing apoptosis-related molecules such as Fas, Fas ligand, tumor necrosis factor-related apoptosis inducing ligand and BAX; and 7) inhibiting effector cells via inhibitory ligands such as PD-L1, cytotoxic T-lymphocyte associated protein 4 and lymphocyte activation gene 3 [47–52]. The types and frequencies of these immune escape mechanisms vary among different cancer types. The proportions and activities of effector cells and antigen-presenting cells such as dendritic cells, B cells and macrophages are often reduced in the peripheral blood of cancer patients, while the numbers of immune-suppressive mesenchymal stem cells, natural killer T cells and regulatory T cells are generally elevated [49, 50]. Using different bioinformatics methods, we demonstrated that *TRIM28* expression correlated negatively with the immune infiltration of LUAD. Thus, we proposed that TRIM28 may negatively regulate the TIME and thereby promote tumor development and progression.

There are several possible mechanisms by which TRIM28 could impair immune cell infiltration. 1) TRIM28 critically inhibits the induction of Foxp3, the number of regulatory T cells, Th17 cells differentiation [20, 53, 54], and macrophage activation [18, 24], suggesting that TRIM28 may impair the TIME. 2) TRIM28 inhibits the effects of IRF5 on gene expression, and IRF5 has been reported to repress anti- inflammatory genes such as *IL-10* [18]. 3) TRIM28 negatively regulates IRF7, which is a potent transcription factor of type I interferons and interferon-stimulated genes and is known as the master regulator of type I interferon-dependent immune responses [22]. 4) TRIM28 suppressed TNF-a–induced IL-6 production and transcriptional activation of NF-kB [24, 25]. 5) TRIM28 functions as an important negative regulator of the expression of IFN-β, IFN-γ, IL-6 and IL-8 during viral infection [55]. We found that TRIM28 may inhibit the interferon-gamma and Toll-like receptor signaling pathways by increasing the SUMOylation of IRF5 and IRF8, ultimately suppressing immune infiltration in LUAD. We confirmed these findings by performing a correlation analysis and validations in LUAD cell lines. Overall, TRIM28 appears to globally manage various immune-related cells and to reduce immune infiltration by altering the expression of diverse chemokines and molecular signaling pathways.

Using a multivariate Cox proportional hazards model, we confirmed that *TRIM28* expression and infiltrating B cell levels were independent predictors of survival in LUAD. Tumor-infiltrating B cell levels are strongly associated with the prognosis of various tumor types [56–59]. The mechanisms by which tumor-infiltrating B cells influence tumor immunity may include: 1) functioning as antigen-presenting cells to facilitate innate cellular immunity in the TIME; 2) activating CD8+ T cells to promote antigen-specific antitumor immune responses [60]; and 3) promoting adaptive immunity by inducing the release of circulating cytokines to recruit immunosuppressive cells [61]. However, further study is needed to understand their functions and mechanisms.

The PD1/PD-L1 cascade is a highly effective therapeutic target in immunotherapy [4–7]. PD-L1 is expressed in a variety of cancer types in either a constitutive (or intrinsic) or interferon-induced manner. The results of our research and previous studies suggest that TRIM28 regulates interferons in multiple ways. Liang et al. [62] reported that verteporfin, a small-molecule inhibitor, inhibited PD-L1 by inducing autophagy and disrupting the STAT1-IRF1-TRIM28 signaling axis, thus exerting antitumor effects in immunotherapy. However, the efficacy of immunotherapy depends not only on immune infiltration and PD-L1 expression, but also on the tumor mutation burden, epidermal growth factor receptor mutation status and other unknown factors [63]. Thus, further research is needed to evaluate the potential of TRIM28 as a therapeutic target in immunotherapy.

There are several limitations to our research. First, this was a retrospective analysis based on public databases (TCGA and GEO). The number of included patients was limited, and it was difficult to account for variations in race, age and geographic area. Thus, additional *in vivo* and *in vitro* experiments are required for functional and clinical verification. Second, considering the possible spatial and temporal heterogeneity of the TIME, immune and stromal assessments should ideally be performed at the core and infiltrating edges of the tumor, respectively. However, all the data in this study were from samples in the core area of the tumor.

In conclusion, the present study demonstrated that TRIM28 worsens the TIME and is highly expressed in LUAD. Increased *TRIM28* expression was associated with reduced levels of various infiltrating immune cells, and was an independent prognostic factor in LUAD. TRIM28 may negatively regulate the TIME by increasing the SUMOylation of IRF5 and IRF8. Thus, our research has provided new insights into the suppressive function of TRIM28 in the TIME and the potential of *TRIM28* as a prognostic biomarker in LUAD.

## MATERIALS AND METHODS

### Gene expression profile data

TCGA data containing RNA sequencing results and clinical information (level 3 data) were downloaded from the cBioPortal database (http://www.cbioportal.org). The *TRIM28* expression data from GSE32863 [33], GSE7670 [34], GSE19188 [35] and the Beer Lund dataset [36] were downloaded from Oncomine (https://www.oncomine.org/resource/main.html) [64]. Two mRNA expression datasets (GSE43580 and GSE7670) were downloaded from the GEO database (https://www.ncbi.nlm.nih.gov/geo/) [34, 45], and were based on the GPL570 (Affymetrix Human Genome U133 Plus 2.0 Array) and GPL96 (Affymetrix Human Genome U133A Array) platforms, respectively. The GSE43580 dataset included 77 LUAD patients, while the GSE7670 dataset included 28 LUAD patients.

### DEG identification

R software (version 3.6.1) was used for detailed analyses in this study. We used the LinkedOmics database (http://www.linkedomics.org/login.php) [44] to analyze the genes co-expressed with *TRIM28* in LUAD. All DEG analyses were performed using the "limma" R package. Fold-changes in gene expression were calculated with threshold criteria of a |log2fold-change| > 0.5, false discovery rate < 0.05 and adjusted *p* < 0.001 for DEG selection.

### Functional enrichment analysis of DEGs

To explore the functions of the overlapping DEGs, we performed GO and KEGG analyses in Metascape (http://metascape.org/gp) [32]. The selected GO terms were from the "Biological Process" annotation datasets. The cutoff value for pathway screening was set to *p* < 0.01. The levels of significant DEGs were visualized on a heatmap based on hierarchical clustering analyses using the average linkage method.

### PPI network construction and analysis

STRING (Version 11.0, http://string-db.org) is a database of known and predicted PPI networks. We used this tool to construct PPI networks and predict potential interactions between candidate genes. Interactions were considered significant above a cutoff score of 0.4. In addition, Cytoscape software (Version 3.7.2, http://www.cytoscape.org/) [65] and the cytoHubba plugin [66] were used to explore the hub genes in the PPI network. Eleven methods can be used to explore essential nodes in PPI networks, but maximal clique centrality performs better than the others.

### RNA and protein expression analyses

Oncomine [64] was used to explore the *TRIM28* levels in different tumor types. The parameters were adjusted according to the following criteria: *p*-value of 1e-4, fold change of 2 and gene ranking in the top 10%.

TIMER (https://cistrome.shinyapps.io/timer/) [67] is a comprehensive database that can be used to estimate the abundance of immune infiltrates and characterize the tumor-immune interactions across diverse tumor types. The levels of six tumor-infiltrating immune subsets, including B cells, CD4+ T cells, CD8+ T cells, neutrophils, macrophages and dendritic cells, are precalculated for 10,897 tumors from TCGA. We used TIMER to explore *TRIM28* expression in various tumor types.

In addition, the UALCAN database [68] can be used to analyze protein expression based on data from the Clinical Proteomic Tumor Analysis Consortium Confirmatory/Discovery dataset. We evaluated the protein levels of TRIM28 in various cancers by performing a Clinical Proteomic Tumor Analysis Consortium analysis.

### Prognosis analysis

The PrognoScan database (http://www.abren.net/PrognoScan/) was used to determine the relationship between *TRIM28* levels and the prognoses of different tumor types [69]. PrognoScan analyzes the correlations between gene levels and prognostic indicators such as DFS and OS using a large number of public tumor microarray datasets. The threshold was set to a Cox *p*-value of 0.05.

Kaplan-Meier plotter (http://kmplot.com/analysis/) [70], an online database of published microarray datasets, can be used to assess the impact of 54,675 genes on survival using 18,674 cancer samples, including samples from 5,143 breast, 1,816 ovarian, 2,437 lung and 1,065 gastric cancer patients. We used the Kaplan-Meier plotter to explore the relationship between *TRIM28* expression and prognosis in breast, lung, ovarian and gastric cancers. The HR, 95% CI and *p*-value were all calculated.

GEPIA (http://gepia.cancer-pku.cn/index.html) [71] is an online database that can be used for differential gene expression analysis, profile plotting, correlation analysis, patient survival analysis, similar gene detection and dimensionality reduction analysis based on TCGA and Genotype-Tissue Expression data. We used GEPIA to analyze the prognostic value of *TRIM28* expression based on the log-rank test in 33 cancer types.

### Stromal and immune score calculation and immune infiltration analysis

Using the "Estimate" R package, we calculated the stromal and immune scores of LUAD patients based on their gene expression profiles [11]. To verify the relationships between the target genes and the TIME, we used the TIMER database and another comprehensive database (TISIDB, http://cis.hku.hk/TISIDB/index.php) [72]. TISIDB integrates multiple heterogeneous data types. Spearman correlations between *TRIM28* levels and tumor-infiltrating lymphocyte levels across human cancers were analyzed. All hypothetical tests were two-sided, and *p*-values < 0.05 were considered significant.

### Query for E3-TRIM28 interactions in UbiBrowser

UbiBrowser (http://ubibrowser.ncpsb.org/) [46] is an integrated bioinformatics platform that can be used to predict proteome-wide human E3-substrate networks based on naïve Bayesian networks. It currently contains 1,295 literature-reported E3-substrate interactions and 8,255 predicted E3-substrate interactions. We used it to predict the potential substrates of TRIM28.

### Cell culture and transfection

We obtained the PC9 cell line from the RIKEN BioResource Center (Tsukuba, Japan), and purchased the H1299 cell line from the American Type Culture Collection (Manassas, VA, USA). The cells were propagated in RPMI 1640 medium (Invitrogen, Carlsbad, CA, USA) supplemented with 10 % fetal bovine serum and antibiotics.

For transfection, the cells (1 × 10^5^) were seeded in six-well plates. The cells were transfected using jetPRIME reagent (Polyplus transfection) with 110 pmol of ON-TARGETplus SMARTpool-Human TRIM28 (L-005046-00-0020, Dharmacon) according to the manufacturers’ protocols.

### Western blotting

The Western blot assay was conducted as previously described. Briefly, the cells were washed in cold 1× phosphate-buffered saline and lysed in ice-cold radioimmunoprecipitation assay buffer supplemented with protease inhibitors on ice for 30 min. The protein concentrations were quantified using the bicinchoninic acid method according to the manufacturer’s instructions. The protein samples were subjected to sodium dodecyl sulfate-polyacrylamide gel electrophoresis and then transferred to nitrocellulose membranes (BioRad, USA). The membranes were blocked with 5% non-fat milk for one hour, and then incubated overnight with primary antibodies diluted in 2% bovine serum albumin. After being washed, the membranes were incubated with horseradish peroxidase-conjugated secondary antibodies for one hour. The primary antibodies used for immunodetection were rabbit monoclonal anti-TRIM28 (#4124, Cell Signaling Technology, USA), rabbit polyclonal anti-IRF5 (#20261, Cell Signaling Technology), rabbit monoclonal anti-IRF8 (#5628, Cell Signaling Technology) and rabbit polyclonal anti-β-actin (#ab8227, Abcam, UK). The secondary antibody was obtained from Abcam (#ab205718). Blots were visualized with an enhanced chemiluminescence reagent (Supersignal; Pierce, Rockford, IL, USA).

### Statistical analysis

The results from the Oncomine database are displayed with *p*-values, fold-changes and ranks. Interactive heatmaps were constructed using Next-Generation Clustered Heatmaps [73]. PrognoScan, GEPIA and Kaplan-Meier plots were used to create survival curves, and the results are displayed with HRs and *p-*values or Cox *p*-values. A multivariate Cox proportional hazards model was used to analyze the independent prognostic factors for lung cancer. *P*-values < 0.05 were considered statistically significant.

## Supplementary Material

Supplementary Figures

Supplementary Table 1

Supplementary Table 2

Supplementary Table 3

Supplementary Table 4
