# Supplementary material for: TRIM28 is a distinct prognostic biomarker that worsens the tumor immune microenvironment in lung adenocarcinoma
Source: Aging (Albany NY). 2020 Oct 22;12(20):20308–31. doi: 10.18632/aging.103804 (PMC7655206; doi:10.18632/aging.103804)
Supplement: Supplementary Figures [file aging-12-103804-s001..pdf]

SUPPLEMENTARY FIGURES

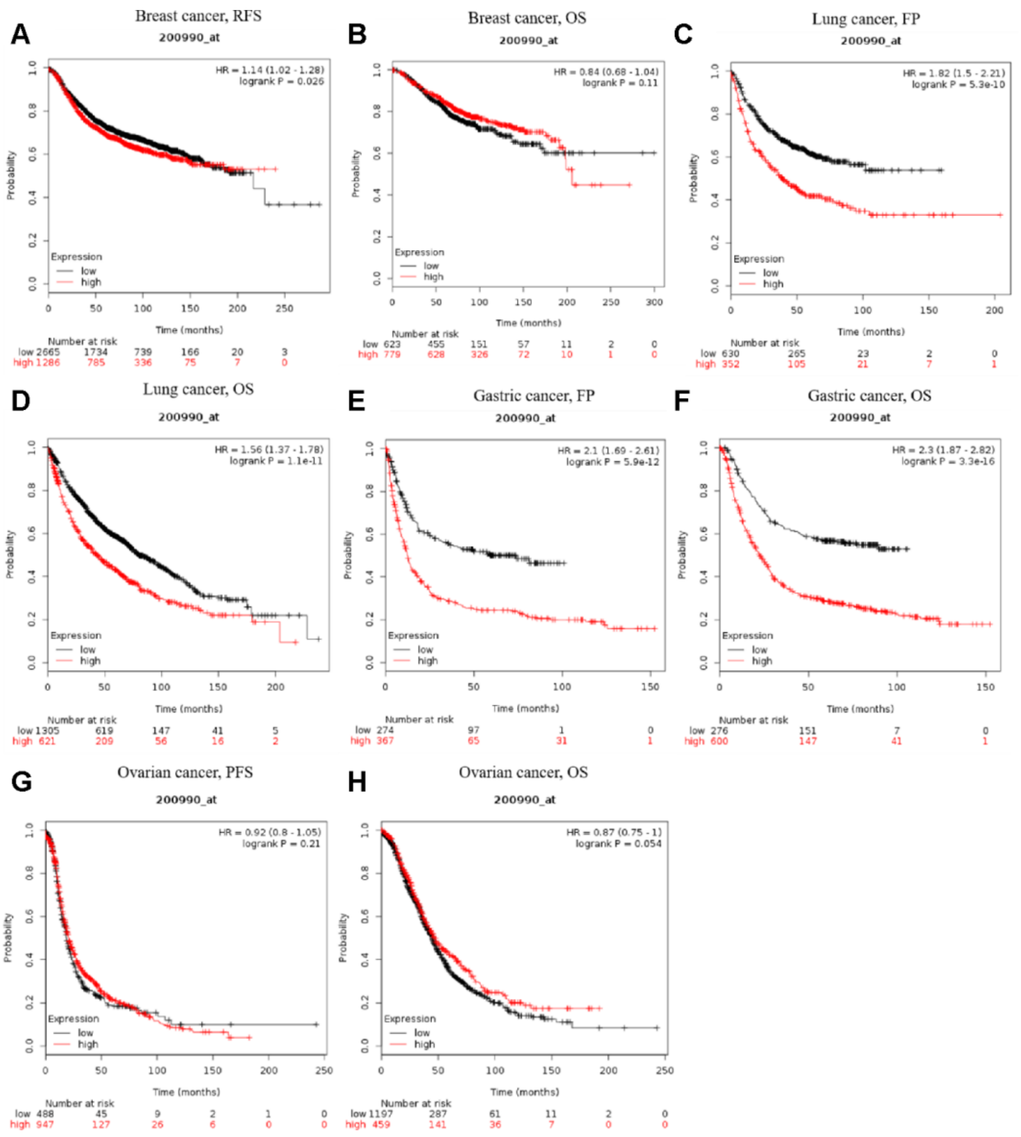

Supplementary Figure 1. Kaplan-Meier survival curves generated using Kaplan-Meier plotter for TRIM28 expression in different tumor types. (**A**, **B**) RFS and OS curves in breast cancer cohorts (n = 1,926, n = 641). (**C**, **D**) FP and OS curves in lung cancer cohorts (n = 982, n = 1,926). (**E**, **F**) FP and OS curves in gastric cancer cohorts (n = 641, n = 876). (**G**, **H**) PFS and OS curves in ovarian cancer cohorts (n = 1,435, n = 1,656). FP, first progression; PFS, Progression-free survival; OS, overall survival; RFS, recurrence-free survival.

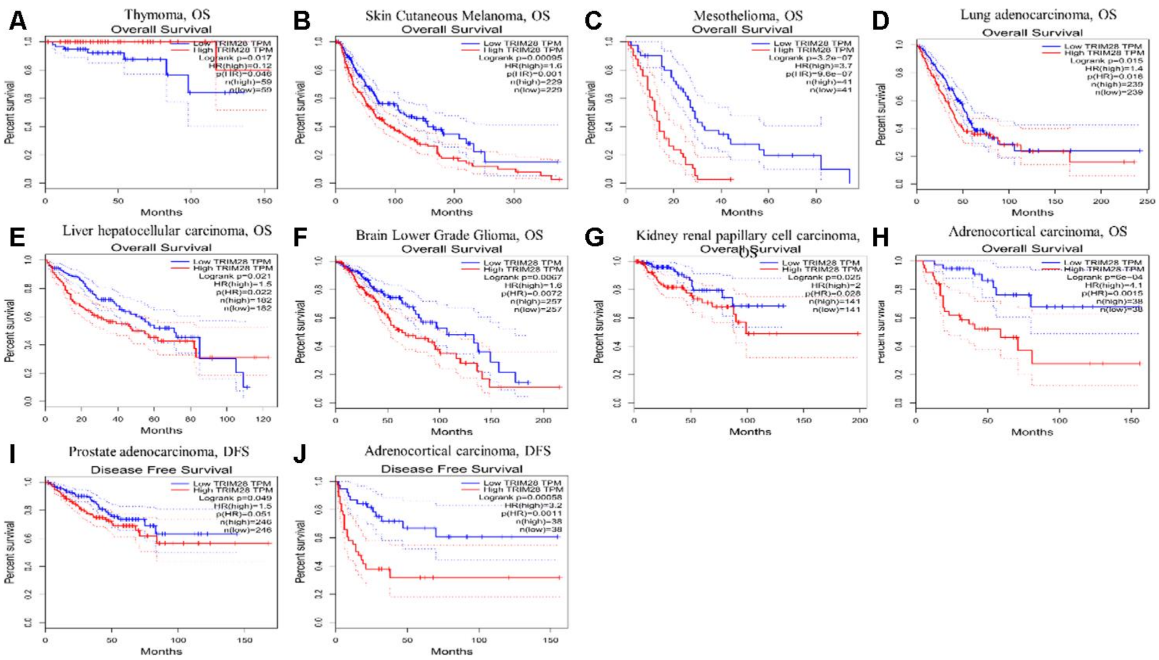

Supplementary Figure 2. Kaplan-Meier survival curves generated from the GEPIA database for TRIM28 expression in different tumor types (**A**–**J**). DFS, disease-free survival; OS, overall survival. 17. Clinical information data of 517 LUAD patients.
