# Supplementary material for: TRIM28 is a distinct prognostic biomarker that worsens the tumor immune microenvironment in lung adenocarcinoma
Source: Aging (Albany NY). 2020 Oct 22;12(20):20308–31. doi: 10.18632/aging.103804 (PMC7655206; doi:10.18632/aging.103804)
Supplement: Supplementary Table 1 [file aging-12-103804-s002..docx]

Supplementary Table 1. Clinical information of 517 LUAD patients.

| Patient ID | M | N | Stage | T | DFS | DFS Status | os | OS Status | Sex |
| --- | --- | --- | --- | --- | --- | --- | --- | --- | --- |
| TCGA-05-4244 | M1 | N2 | Stage IV | T2 | 0 | DiseaseFree | 0 | LIVING | MALE |
| TCGA-05-4249 | M0 | N0 | Stage IB | T2 | 50 | DiseaseFree | 50.03 | LIVING | Male |
| TCGA-05-4250 | M0 | N1 | Stage IIIA | T3 | NA | NA | 3.98 | DECEASED | Female |
| TCGA-05-4382 | M0 | N0 | Stage IB | T2 | 11 | Recurred/Progressed | 19.94 | LIVING | Male |
| TCGA-05-4384 | M0 | N2 | Stage IIIA | T2 | 6 | Recurred/Progressed | 13.99 | LIVING | Male |
| TCGA-05-4389 | M0 | N0 | Stage IA | T1 | 45 | DiseaseFree | 44.97 | LIVING | Male |
| TCGA-05-4390 | M0 | N0 | Stage IB | T2 | 13 | Recurred/Progressed | 36.99 | LIVING | Female |
| TCGA-05-4395 | M0 | N2 | Stage IIIB | T4 | NA | NA | 0 | DECEASED | Male |
| TCGA-05-4396 | M0 | N1 | Stage IIIB | T4 | NA | NA | 9.95 | DECEASED | Male |
| TCGA-05-4397 | M0 | N1 | Stage IIB | T2 | NA | NA | 24.01 | DECEASED | Male |
| TCGA-05-4398 | M0 | N3 | Stage IIIB | T4 | 47 | DiseaseFree | 47.01 | LIVING | Female |
| TCGA-05-4402 | M1 | NX | Stage IV | T2 | NA | NA | 8.02 | DECEASED | Female |
| TCGA-05-4403 | M0 | N0 | Stage IB | T2 | 19 | DiseaseFree | 18.99 | LIVING | Male |
| TCGA-05-4405 | M0 | N0 | Stage IB | T2 | 20 | DiseaseFree | 20.04 | LIVING | Female |
| TCGA-05-4410 | M0 | N0 | Stage IB | T2 | 0 | DiseaseFree | 0 | LIVING | Male |
| TCGA-05-4415 | M0 | N2 | Stage IIIB | T4 | 2 | Recurred/Progressed | 2.99 | DECEASED | Male |
| TCGA-05-4417 | M0 | N0 | Stage IB | T2 | 15 | DiseaseFree | 14.95 | LIVING | Female |
| TCGA-05-4418 | M0 | N2 | Stage IIIA | T3 | NA | NA | 9 | DECEASED | Male |
| TCGA-05-4420 | M0 | N0 | Stage IB | T2 | 30 | DiseaseFree | 29.96 | LIVING | Male |
| TCGA-05-4422 | M0 | N0 | Stage IB | T2 | 12 | DiseaseFree | 11.99 | LIVING | Male |
| TCGA-05-4424 | M0 | N0 | Stage IIB | T3 | 5 | Recurred/Progressed | 29.99 | LIVING | Male |
| TCGA-05-4425 | M1 | N0 | Stage IV | T2 | 22 | DiseaseFree | 21.98 | LIVING | Female |
| TCGA-05-4426 | M0 | N0 | Stage IB | T2 | 15 | Recurred/Progressed | 25.99 | LIVING | Male |
| TCGA-05-4427 | M0 | N1 | Stage IIB | T2 | 26 | DiseaseFree | 25.99 | LIVING | Female |
| TCGA-05-4430 | M0 | N0 | Stage IB | T2 | 25 | DiseaseFree | 25 | LIVING | Female |
| TCGA-05-4432 | M0 | N1 | Stage IIB | T2 | 25 | DiseaseFree | 25 | LIVING | Male |
| TCGA-05-4433 | M0 | N0 | Stage IB | T2 | 24 | DiseaseFree | 23.98 | LIVING | Male |
| TCGA-05-4434 | M1 | N1 | Stage IV | T4 | NA | NA | 15.01 | DECEASED | Female |
| TCGA-05-5420 | M0 | N2 | Stage IIIA | T2 | 8.1 | Recurred/Progressed | 15.01 | LIVING | Male |
| TCGA-05-5423 | M0 | N1 | Stage IIB | T2 | 5 | DiseaseFree | 4.96 | LIVING | Male |
| TCGA-05-5425 | M0 | N1 | Stage IIB | T2b | 16 | Recurred/Progressed | 28.98 | LIVING | Male |
| TCGA-05-5428 | M0 | N1 | Stage IIA | T1b | 22 | DiseaseFree | 22.01 | LIVING | Male |
| TCGA-05-5429 | M0 | N2 | Stage IIIA | T3 | NA | NA | 9.03 | DECEASED | Male |
| TCGA-05-5715 | M0 | N0 | Stage IB | T2a | 2 | DiseaseFree | 2.04 | LIVING | Female |
| TCGA-35-3615 | M0 | N0 | Stage IB | T2 | 0.5 | DiseaseFree | 0.46 | LIVING | Male |
| TCGA-35-4122 | M0 | N0 | Stage IA | T1 | 7.4 | DiseaseFree | 7.39 | LIVING | Male |
| TCGA-35-4123 | M0 | N0 | Stage IA | T1 | 6 | DiseaseFree | 5.98 | LIVING | Male |
| TCGA-35-5375 | M0 | N2 | Stage IIIA | T2 | 8.7 | DiseaseFree | 8.67 | LIVING | Male |
| TCGA-38-4625 | M0 | N0 | Stage IB | T2a | 98 | DiseaseFree | 97.67 | LIVING | Female |
| TCGA-38-4626 | M0 | N0 | NA | T2b | 83 | Recurred/Progressed | 120.7 | LIVING | Female |
| TCGA-38-4627 | M0 | N1 | Stage IIA | T1b | NA | NA | 37.68 | DECEASED | Female |
| TCGA-38-4628 | M0 | N1 | Stage IIB | T2 | 36 | Recurred/Progressed | 49.01 | DECEASED | Female |
| TCGA-38-4629 | M0 | N0 | Stage IIB | T3 | 12 | Recurred/Progressed | 28.38 | DECEASED | Male |
| TCGA-38-4630 | M0 | N0 | Stage IB | T2 | 27 | Recurred/Progressed | 35.25 | DECEASED | Female |
| TCGA-38-4631 | M0 | N0 | Stage IB | T2 | NA | NA | 11.63 | DECEASED | Female |
| TCGA-38-4632 | M1 | N1 | Stage IV | T2 | 30 | Recurred/Progressed | 44.58 | DECEASED | Male |
| TCGA-38-6178 | NA | N2 | Stage IIIA | T2b | 15 | DiseaseFree | 14.72 | LIVING | Female |
| TCGA-38-7271 | M0 | N0 | Stage IA | T1 | 21 | Recurred/Progressed | 26.28 | DECEASED | Female |
| TCGA-38-A44F | M0 | N0 | Stage IB | T2a | 4.4 | DiseaseFree | 4.37 | LIVING | Male |
| TCGA-44-2655 | M0 | N0 | Stage IA | T1 | 33 | Recurred/Progressed | 43.5 | LIVING | Female |
| TCGA-44-2656 | M0 | N0 | Stage IB | T2 | 19 | Recurred/Progressed | 46.94 | LIVING | Male |
| TCGA-44-2657 | M0 | NX | Stage IB | T2 | 44 | DiseaseFree | 44.38 | LIVING | Female |
| TCGA-44-2659 | M0 | N1 | Stage IIB | T1 | 38 | Recurred/Progressed | 44.91 | LIVING | Female |
| TCGA-44-2661 | M0 | N0 | Stage IA | T1 | 38 | DiseaseFree | 38.07 | LIVING | Female |
| TCGA-44-2662 | M0 | N0 | Stage IB | T2 | 8.1 | Recurred/Progressed | 42.05 | LIVING | Male |
| TCGA-44-2665 | M0 | N1 | Stage IIB | T2 | 43 | DiseaseFree | 42.74 | LIVING | Female |
| TCGA-44-2666 | M0 | N0 | Stage IB | T2 | NA | NA | 3.19 | DECEASED | Male |
| TCGA-44-2668 | M0 | N0 | Stage IB | T2 | 14 | Recurred/Progressed | 25 | DECEASED | Male |
| TCGA-44-3396 | M0 | N2 | Stage IIIA | T2 | 37 | DiseaseFree | 37.12 | LIVING | Female |
| TCGA-44-3398 | M0 | N0 | Stage IA | T1b | 38 | DiseaseFree | 38.21 | LIVING | Female |
| TCGA-44-3918 | M0 | N0 | Stage IA | T1 | 17 | Recurred/Progressed | 34.03 | LIVING | Female |
| TCGA-44-3919 | M0 | N0 | Stage IA | T1 | 32 | Recurred/Progressed | 33.71 | DECEASED | Female |
| TCGA-44-4112 | M0 | N0 | Stage IB | T2a | 20 | Recurred/Progressed | 26.54 | DECEASED | Female |
| TCGA-44-5643 | M0 | N2 | Stage IIIA | T2b | 33 | DiseaseFree | 33.28 | LIVING | Male |
| TCGA-44-5644 | NA | N0 | Stage IB | T2a | 28 | DiseaseFree | 28.35 | LIVING | Female |
| TCGA-44-5645 | NA | NX | Stage IA | T1 | 28 | DiseaseFree | 27.99 | LIVING | Female |
| TCGA-44-6145 | M0 | N0 | Stage IA | T1 | 20 | DiseaseFree | 19.55 | LIVING | Female |
| TCGA-44-6146 | M0 | N0 | Stage IIB | T3 | 21 | Recurred/Progressed | 23.92 | LIVING | Male |
| TCGA-44-6147 | M0 | NX | Stage IA | T1b | 28 | DiseaseFree | 27.76 | LIVING | Female |
| TCGA-44-6148 | M0 | N0 | Stage IA | T1b | 23 | DiseaseFree | 23.13 | LIVING | Male |
| TCGA-44-6774 | M0 | N2 | Stage IIIA | T1 | 22 | DiseaseFree | 21.62 | LIVING | Female |
| TCGA-44-6775 | MX | N0 | Stage IB | T2a | 22 | Recurred/Progressed | 23.16 | LIVING | Female |
| TCGA-44-6776 | MX | N0 | Stage IA | T1 | 86 | DiseaseFree | 85.94 | LIVING | Female |
| TCGA-44-6777 | MX | NX | Stage IB | T2 | NA | NA | 32.42 | DECEASED | Female |
| TCGA-44-6778 | MX | N0 | Stage IA | T1 | 61 | DiseaseFree | 61.24 | LIVING | Male |
| TCGA-44-6779 | MX | N1 | Stage IIB | T2 | 7.6 | Recurred/Progressed | 16.43 | DECEASED | Female |
| TCGA-44-7659 | MX | N0 | Stage IA | T1b | 23 | DiseaseFree | 22.7 | LIVING | Male |
| TCGA-44-7660 | MX | N0 | Stage IB | T2 | 8.3 | Recurred/Progressed | 19.45 | LIVING | Male |
| TCGA-44-7661 | M0 | N0 | Stage IB | T2a | 11 | Recurred/Progressed | 18.3 | DECEASED | Female |
| TCGA-44-7662 | MX | N0 | Stage IB | T2a | 7.2 | DiseaseFree | 7.16 | LIVING | Male |
| TCGA-44-7667 | MX | N0 | Stage IIB | T3 | 36 | DiseaseFree | 36.04 | LIVING | Female |
| TCGA-44-7669 | MX | N1 | Stage IIA | T1b | 17 | Recurred/Progressed | 18.86 | DECEASED | Male |
| TCGA-44-7670 | M0 | N1 | Stage IIA | T1b | 29 | DiseaseFree | 28.98 | LIVING | Female |
| TCGA-44-7671 | M0 | N0 | Stage IB | T2a | 29 | Recurred/Progressed | 29.2 | LIVING | Male |
| TCGA-44-7672 | M0 | N0 | Stage IA | T1b | 24 | DiseaseFree | 23.62 | LIVING | Female |
| TCGA-44-8117 | M0 | N0 | Stage IB | T2a | 13 | DiseaseFree | 12.65 | LIVING | Female |
| TCGA-44-8119 | M0 | N0 | Stage IIB | T3 | 9.4 | DiseaseFree | 9.36 | LIVING | Male |
| TCGA-44-8120 | M0 | N0 | Stage IB | T2a | 8.5 | DiseaseFree | 8.54 | LIVING | Male |
| TCGA-44-A479 | MX | N0 | Stage IB | T2 | 14 | Recurred/Progressed | 15.97 | LIVING | Female |
| TCGA-44-A47A | MX | N0 | Stage IB | T2a | 13 | Recurred/Progressed | 15.31 | LIVING | Female |
| TCGA-44-A47B | M0 | N0 | Stage IB | T2a | 9.4 | DiseaseFree | 9.43 | LIVING | Male |
| TCGA-44-A47G | M0 | N0 | Stage IA | T1 | 12 | DiseaseFree | 11.53 | LIVING | Female |
| TCGA-44-A4SS | M0 | N0 | Stage IA | T1b | 14 | DiseaseFree | 13.63 | LIVING | Male |
| TCGA-44-A4SU | MX | N0 | Stage IA | T1a | 8.5 | Recurred/Progressed | 13.44 | DECEASED | Female |
| TCGA-49-4486 | M0 | N0 | Stage IA | T1 | 67 | Recurred/Progressed | 76.15 | DECEASED | Male |
| TCGA-49-4487 | M0 | N0 | Stage IA | T1 | 23 | Recurred/Progressed | 28.09 | DECEASED | Female |
| TCGA-49-4488 | MX | N0 | Stage IA | T1 | 21 | Recurred/Progressed | 28.55 | DECEASED | Female |
| TCGA-49-4490 | M0 | N2 | Stage IIIA | T3 | NA | NA | 12.65 | DECEASED | Female |
| TCGA-49-4494 | M0 | N2 | Stage IIIA | T3 | NA | NA | 35.51 | DECEASED | Male |
| TCGA-49-4501 | M0 | N0 | Stage IB | T2 | 18 | Recurred/Progressed | 46.68 | DECEASED | Female |
| TCGA-49-4505 | M0 | N1 | Stage IIB | T2 | 14 | Recurred/Progressed | 14.06 | DECEASED | Female |
| TCGA-49-4506 | M0 | N1 | Stage IIB | T2 | 23 | Recurred/Progressed | 32.82 | DECEASED | Female |
| TCGA-49-4507 | M0 | N1 | Stage IIIA | T3 | 5.2 | Recurred/Progressed | 8.8 | DECEASED | Female |
| TCGA-49-4510 | M0 | N1 | Stage IIB | T2 | 16 | Recurred/Progressed | 29.43 | DECEASED | Female |
| TCGA-49-4512 | MX | N2 | Stage IIIA | T2 | NA | NA | 29.73 | DECEASED | Female |
| TCGA-49-4514 | M0 | N0 | Stage IA | T1 | 56 | DiseaseFree | 55.85 | LIVING | Female |
| TCGA-49-6742 | M0 | N1 | Stage IIA | T2a | 7 | Recurred/Progressed | 16.03 | DECEASED | Male |
| TCGA-49-6743 | MX | N2 | Stage IIIA | T1 | 53 | DiseaseFree | 53.25 | LIVING | Female |
| TCGA-49-6744 | MX | N1 | Stage IIA | T2a | 55 | DiseaseFree | 55.29 | LIVING | Female |
| TCGA-49-6745 | M0 | N2 | Stage IIIA | T2a | 17 | DiseaseFree | 17.15 | LIVING | Male |
| TCGA-49-6761 | MX | N2 | Stage IIIA | T1 | 12 | DiseaseFree | 11.63 | LIVING | Female |
| TCGA-49-6767 | MX | N0 | Stage IIB | T3 | 22 | DiseaseFree | 22.24 | LIVING | Female |
| TCGA-49-AAQV | MX | N1 | Stage II | T1 | 17 | Recurred/Progressed | 22.24 | DECEASED | Female |
| TCGA-49-AAR0 | MX | N0 | Stage IA | T1 | 157 | DiseaseFree | 156.54 | LIVING | Male |
| TCGA-49-AAR2 | MX | N0 | Stage IB | T2 | 73 | DiseaseFree | 73.06 | LIVING | Male |
| TCGA-49-AAR3 | MX | N1 | Stage IIB | T2 | 62 | Recurred/Progressed | 62.19 | LIVING | Male |
| TCGA-49-AAR4 | MX | N2 | Stage IIIA | T2 | NA | NA | 28.88 | DECEASED | Male |
| TCGA-49-AAR9 | MX | N0 | Stage IIB | T3 | NA | NA | 8.54 | DECEASED | Male |
| TCGA-49-AARE | MX | N0 | Stage IA | T1 | 13 | Recurred/Progressed | 40.37 | DECEASED | Female |
| TCGA-49-AARN | MX | N0 | Stage IA | T1 | NA | NA | 37.29 | DECEASED | Female |
| TCGA-49-AARO | MX | N0 | Stage IA | T1a | 116 | Recurred/Progressed | 123.49 | LIVING | Female |
| TCGA-49-AARQ | MX | N0 | Stage I | T2 | 221 | DiseaseFree | 221.16 | LIVING | Female |
| TCGA-49-AARR | MX | N0 | Stage IA | T1 | 158 | Recurred/Progressed | 163.99 | LIVING | Male |
| TCGA-4B-A93V | M0 | N0 | Stage IA | T1b | 8.3 | Recurred/Progressed | 9.86 | DECEASED | Female |
| TCGA-50-5044 | M0 | N1 | Stage IIIB | T4 | 16 | Recurred/Progressed | 20.5 | DECEASED | Female |
| TCGA-50-5045 | M0 | N1 | NA | T2 | 47 | Recurred/Progressed | 71.42 | DECEASED | Female |
| TCGA-50-5049 | M0 | N0 | Stage IA | T2 | 84 | Recurred/Progressed | 101.64 | LIVING | Male |
| TCGA-50-5051 | M0 | N2 | Stage IIIA | T2 | 6 | Recurred/Progressed | 15.7 | DECEASED | Female |
| TCGA-50-5055 | M0 | N1 | Stage IIA | T1 | 25 | Recurred/Progressed | 60.12 | DECEASED | Female |
| TCGA-50-5066 | M0 | N0 | Stage IB | T2 | 16 | Recurred/Progressed | 47.37 | LIVING | Male |
| TCGA-50-5066 | M0 | N0 | Stage IB | T2 | 16 | Recurred/Progressed | 47.37 | LIVING | Male |
| TCGA-50-5068 | MX | N1 | Stage IIB | T2 | 23 | Recurred/Progressed | 49.24 | DECEASED | Female |
| TCGA-50-5072 | M0 | N2 | Stage IIIA | T2 | 7 | Recurred/Progressed | 8.21 | DECEASED | Male |
| TCGA-50-5930 | M0 | N2 | Stage IIIA | T2 | 5.8 | Recurred/Progressed | 9.26 | DECEASED | Male |
| TCGA-50-5931 | M0 | N0 | Stage IB | T2 | 14 | Recurred/Progressed | 14.26 | DECEASED | Female |
| TCGA-50-5932 | M0 | N1 | Stage IIB | T2 | 36 | Recurred/Progressed | 40.57 | DECEASED | Male |
| TCGA-50-5933 | M0 | N2 | Stage IIIB | T4 | NA | NA | 78.61 | DECEASED | Male |
| TCGA-50-5935 | M0 | N0 | Stage IA | T1 | NA | NA | 21.45 | DECEASED | Female |
| TCGA-50-5936 | M0 | N2 | Stage IIIA | T2 | 4.9 | Recurred/Progressed | 8.44 | DECEASED | Male |
| TCGA-50-5939 | M0 | N0 | Stage IB | T2 | NA | NA | 15.11 | DECEASED | Male |
| TCGA-50-5941 | M0 | N2 | Stage IIIA | T2a | 48 | DiseaseFree | 48.42 | LIVING | Female |
| TCGA-50-5942 | M0 | N0 | Stage IA | T1 | 45 | Recurred/Progressed | 60.68 | LIVING | Female |
| TCGA-50-5944 | M0 | N0 | Stage IA | T1 | 57 | DiseaseFree | 57.49 | LIVING | Female |
| TCGA-50-5946 | MX | N0 | Stage IA | T1 | 27 | Recurred/Progressed | 53.12 | LIVING | Male |
| TCGA-50-5946 | MX | N0 | Stage IA | T1 | 27 | Recurred/Progressed | 53.12 | LIVING | Male |
| TCGA-50-6590 | M0 | N0 | Stage IB | T2 | NA | NA | 42.31 | DECEASED | Female |
| TCGA-50-6591 | M1 | N0 | Stage IV | T2 | NA | NA | 3.91 | DECEASED | Female |
| TCGA-50-6592 | M0 | N0 | Stage IB | T2 | NA | NA | 25.53 | DECEASED | Female |
| TCGA-50-6593 | M0 | N2 | Stage IIIA | T1 | 8.7 | Recurred/Progressed | 11.04 | DECEASED | Female |
| TCGA-50-6594 | M0 | N2 | Stage IIIA | T3 | 9.4 | Recurred/Progressed | 12.16 | DECEASED | Female |
| TCGA-50-6595 | M0 | N2 | Stage IIIA | T2 | 6 | Recurred/Progressed | 6.21 | DECEASED | Female |
| TCGA-50-6597 | M0 | N0 | Stage IB | T2 | NA | NA | 41.66 | DECEASED | Female |
| TCGA-50-6673 | M0 | N0 | Stage I | T1 | NA | NA | 0.72 | DECEASED | Female |
| TCGA-50-7109 | M0 | N0 | Stage IA | T1 | 0.5 | Recurred/Progressed | 10.12 | DECEASED | Male |
| TCGA-50-8457 | M0 | N0 | Stage IA | T1a | 37 | DiseaseFree | 36.96 | LIVING | Female |
| TCGA-50-8459 | M0 | N0 | Stage IIB | T3 | 14 | Recurred/Progressed | 36.76 | LIVING | Male |
| TCGA-50-8460 | M0 | N0 | Stage IA | T1a | 27 | DiseaseFree | 27.23 | LIVING | Male |
| TCGA-53-7624 | M1 | N0 | Stage IV | T2 | 13 | Recurred/Progressed | 34.26 | DECEASED | Female |
| TCGA-53-7626 | M0 | N1 | Stage IIA | T1 | 28 | Recurred/Progressed | 30.52 | DECEASED | Female |
| TCGA-53-7813 | M0 | N0 | Stage IIIB | T4 | 14 | DiseaseFree | 13.93 | LIVING | Female |
| TCGA-53-A4EZ | MX | N1 | Stage IIA | T2a | 35 | DiseaseFree | 35.18 | LIVING | Male |
| TCGA-55-1592 | M0 | N0 | Stage IA | T2 | 15 | Recurred/Progressed | 23.03 | DECEASED | Male |
| TCGA-55-1594 | M0 | N2 | Stage IIIA | T2 | 39 | DiseaseFree | 38.7 | LIVING | Male |
| TCGA-55-1595 | M0 | N0 | Stage IA | T1 | 49 | DiseaseFree | 48.59 | LIVING | Female |
| TCGA-55-1596 | M0 | N1 | Stage IIB | T2 | 68 | DiseaseFree | 67.84 | LIVING | Male |
| TCGA-55-5899 | M0 | N1 | NA | T1a | 31 | DiseaseFree | 30.55 | LIVING | Male |
| TCGA-55-6543 | MX | N0 | Stage IA | T1b | 14 | DiseaseFree | 14.29 | LIVING | Female |
| TCGA-55-6642 | MX | N0 | Stage IB | T2 | 80 | DiseaseFree | 80.45 | LIVING | Male |
| TCGA-55-6712 | MX | N1 | Stage IIA | T2a | NA | NA | 5.62 | DECEASED | Male |
| TCGA-55-6968 | M1 | N0 | Stage IV | T1 | NA | NA | 42.48 | DECEASED | Male |
| TCGA-55-6969 | M0 | N0 | Stage IB | T2 | 41 | DiseaseFree | 40.7 | LIVING | Male |
| TCGA-55-6970 | MX | N2 | Stage IIIA | T2 | 15 | Recurred/Progressed | 15.24 | DECEASED | Female |
| TCGA-55-6971 | MX | N0 | Stage IB | T2 | 46 | DiseaseFree | 45.99 | LIVING | Female |
| TCGA-55-6972 | M0 | N0 | Stage IB | T2 | NA | NA | 53.61 | DECEASED | Male |
| TCGA-55-6975 | M0 | N1 | Stage IIB | T2 | NA | NA | 3.88 | DECEASED | Male |
| TCGA-55-6978 | MX | N0 | Stage IIA | T2b | 1.4 | Recurred/Progressed | 5.78 | DECEASED | Male |
| TCGA-55-6979 | M0 | N1 | Stage IIB | T2 | 6.4 | Recurred/Progressed | 7.79 | DECEASED | Female |
| TCGA-55-6980 | M0 | N0 | Stage IA | T1 | 69 | DiseaseFree | 69.28 | LIVING | Male |
| TCGA-55-6981 | M0 | N2 | Stage IIIA | T1 | NA | NA | 45.3 | DECEASED | Female |
| TCGA-55-6982 | M0 | N1 | Stage IIB | T2 | 6 | Recurred/Progressed | 32.69 | DECEASED | Female |
| TCGA-55-6983 | M0 | N1 | Stage IIB | T2 | 93 | DiseaseFree | 92.74 | LIVING | Male |
| TCGA-55-6984 | M0 | N1 | Stage IIB | T2 | 24 | Recurred/Progressed | 24.97 | DECEASED | Female |
| TCGA-55-6985 | MX | N0 | Stage IB | T2 | 41 | DiseaseFree | 40.51 | LIVING | Female |
| TCGA-55-6986 | M0 | N0 | Stage IB | T2 | 107 | DiseaseFree | 107.13 | LIVING | Female |
| TCGA-55-6987 | M0 | N0 | Stage IA | T1 | 70 | DiseaseFree | 70.2 | LIVING | Male |
| TCGA-55-7227 | MX | N1 | Stage IIIA | T3 | 8.4 | Recurred/Progressed | 31.27 | DECEASED | Male |
| TCGA-55-7281 | M0 | N0 | Stage IA | T1b | 11 | Recurred/Progressed | 28.65 | LIVING | Female |
| TCGA-55-7283 | MX | N2 | Stage IIIA | T3 | 20 | DiseaseFree | 20.01 | LIVING | Female |
| TCGA-55-7284 | MX | N0 | Stage IIB | T3 | 7.6 | Recurred/Progressed | 7.98 | DECEASED | Male |
| TCGA-55-7570 | MX | N0 | Stage IA | T1a | 27 | DiseaseFree | 27.07 | LIVING | Male |
| TCGA-55-7573 | MX | N0 | Stage IA | T1b | 16 | DiseaseFree | 16 | LIVING | Female |
| TCGA-55-7574 | M0 | N0 | Stage IB | T2a | 16 | Recurred/Progressed | 32.69 | DECEASED | Female |
| TCGA-55-7576 | M0 | N0 | Stage IB | T2a | 22 | DiseaseFree | 22.01 | LIVING | Male |
| TCGA-55-7724 | MX | N0 | Stage IB | T2a | 23 | DiseaseFree | 23.16 | LIVING | Female |
| TCGA-55-7725 | MX | N0 | Stage IA | T1a | 15 | DiseaseFree | 14.52 | LIVING | Female |
| TCGA-55-7726 | MX | N0 | Stage IA | T1b | 21 | DiseaseFree | 21.42 | LIVING | Female |
| TCGA-55-7727 | MX | N2 | Stage IIIA | T1a | 3.9 | DiseaseFree | 3.91 | LIVING | Male |
| TCGA-55-7728 | MX | N0 | Stage IB | T2a | 23 | DiseaseFree | 23.13 | LIVING | Female |
| TCGA-55-7815 | MX | N0 | Stage IB | T2a | 19 | Recurred/Progressed | 25.39 | LIVING | Male |
| TCGA-55-7816 | MX | NX | Stage IV | TX | NA | NA | 15.37 | DECEASED | Female |
| TCGA-55-7903 | MX | N0 | Stage IA | T1b | 19 | DiseaseFree | 18.63 | LIVING | Male |
| TCGA-55-7907 | MX | N1 | Stage IIA | T2a | 9.7 | Recurred/Progressed | 11.27 | DECEASED | Male |
| TCGA-55-7910 | M0 | N0 | Stage IIA | T2b | 33 | Recurred/Progressed | 34.17 | LIVING | Female |
| TCGA-55-7911 | MX | N0 | Stage IA | T1a | 17 | Recurred/Progressed | 17.64 | LIVING | Female |
| TCGA-55-7913 | MX | N0 | Stage IA | T1b | 16 | Recurred/Progressed | 18.43 | DECEASED | Female |
| TCGA-55-7914 | MX | N1 | Stage IIA | T1b | NA | NA | 6.14 | DECEASED | Female |
| TCGA-55-7994 | MX | N0 | Stage IIB | T3 | 20 | DiseaseFree | 19.81 | LIVING | Male |
| TCGA-55-7995 | M0 | N0 | Stage IA | T1b | 15 | Recurred/Progressed | 29.2 | LIVING | Female |
| TCGA-55-8085 | M0 | N0 | Stage IA | T1b | 30 | DiseaseFree | 29.7 | LIVING | Male |
| TCGA-55-8087 | MX | N0 | Stage IB | T2a | 15 | DiseaseFree | 15.18 | LIVING | Female |
| TCGA-55-8089 | M0 | N0 | Stage IA | T1a | NA | NA | 23.06 | DECEASED | Male |
| TCGA-55-8090 | M0 | N0 | Stage IA | T1a | 18 | Recurred/Progressed | 19.65 | DECEASED | Male |
| TCGA-55-8091 | MX | N0 | Stage IB | T2 | 20 | DiseaseFree | 19.71 | LIVING | Male |
| TCGA-55-8092 | MX | N0 | Stage IIB | T3 | 4.2 | Recurred/Progressed | 5.06 | DECEASED | Male |
| TCGA-55-8094 | M1b | N0 | Stage IV | T2b | 18 | DiseaseFree | 17.77 | LIVING | Male |
| TCGA-55-8096 | MX | N0 | Stage IB | T2a | 19 | Recurred/Progressed | 23.62 | DECEASED | Female |
| TCGA-55-8097 | MX | N0 | Stage IA | T1a | 16 | DiseaseFree | 15.64 | LIVING | Female |
| TCGA-55-8203 | M0 | N0 | Stage IA | T1b | 18 | DiseaseFree | 17.97 | LIVING | Female |
| TCGA-55-8204 | MX | N0 | Stage IB | T2a | 17 | DiseaseFree | 16.92 | LIVING | Female |
| TCGA-55-8205 | M0 | N0 | Stage IIA | T2b | 16 | Recurred/Progressed | 19.68 | LIVING | Female |
| TCGA-55-8206 | M0 | N0 | Stage IA | T1b | 29 | DiseaseFree | 29.17 | LIVING | Male |
| TCGA-55-8207 | MX | N0 | Stage IB | T2a | 32 | DiseaseFree | 32.1 | LIVING | Male |
| TCGA-55-8208 | M0 | N0 | Stage IA | T1b | 17 | Recurred/Progressed | 22.14 | LIVING | Female |
| TCGA-55-8299 | MX | N0 | Stage IA | T1b | 9 | Recurred/Progressed | 15.41 | DECEASED | Female |
| TCGA-55-8301 | MX | N0 | Stage IB | T2a | 7.8 | Recurred/Progressed | 17.54 | LIVING | Male |
| TCGA-55-8302 | MX | N0 | Stage IB | T2 | 16 | DiseaseFree | 15.7 | LIVING | Male |
| TCGA-55-8505 | MX | N2 | Stage IIIA | T1a | 14 | DiseaseFree | 14.45 | LIVING | Male |
| TCGA-55-8506 | MX | N0 | Stage IIB | T3 | 0.4 | DiseaseFree | 0.36 | LIVING | Female |
| TCGA-55-8507 | MX | N0 | Stage IA | T1a | 14 | DiseaseFree | 13.73 | LIVING | Male |
| TCGA-55-8508 | MX | N1 | Stage IIA | T2a | 20 | DiseaseFree | 20.27 | LIVING | Female |
| TCGA-55-8510 | MX | N0 | Stage IB | T2a | 18 | DiseaseFree | 17.71 | LIVING | Female |
| TCGA-55-8511 | MX | N0 | Stage IB | T2a | 15 | Recurred/Progressed | 18.13 | LIVING | Female |
| TCGA-55-8512 | M1b | N1 | Stage IV | T1a | NA | NA | 19.94 | DECEASED | Male |
| TCGA-55-8513 | MX | N0 | Stage IIB | T3 | 10 | Recurred/Progressed | 25.99 | LIVING | Female |
| TCGA-55-8514 | MX | N0 | Stage IB | T2a | 17 | DiseaseFree | 17.08 | LIVING | Female |
| TCGA-55-8614 | MX | N0 | Stage IB | T2a | 18 | DiseaseFree | 17.61 | LIVING | Male |
| TCGA-55-8615 | MX | N2 | Stage IIIA | T3 | 7.4 | Recurred/Progressed | 14.65 | LIVING | Male |
| TCGA-55-8616 | M0 | N0 | Stage IB | T2a | 1.6 | DiseaseFree | 1.58 | LIVING | Female |
| TCGA-55-8619 | MX | N0 | Stage IIB | T3 | 14 | DiseaseFree | 13.67 | LIVING | Female |
| TCGA-55-8620 | M1b | N1 | Stage IV | T1a | NA | NA | 12.32 | DECEASED | Male |
| TCGA-55-8621 | MX | N0 | Stage IA | T1a | 17 | DiseaseFree | 16.92 | LIVING | Female |
| TCGA-55-A48X | M0 | N1 | Stage IIA | T1b | 20 | Recurred/Progressed | 22.63 | LIVING | Female |
| TCGA-55-A48Y | M0 | N0 | Stage IIA | T2b | 21 | DiseaseFree | 20.7 | LIVING | Male |
| TCGA-55-A48Z | MX | N3 | Stage IIIB | T1a | 18 | Recurred/Progressed | 21.39 | LIVING | Female |
| TCGA-55-A490 | MX | N0 | Stage IIA | T2b | NA | NA | 3.25 | DECEASED | Male |
| TCGA-55-A491 | MX | N0 | Stage IA | T1b | 21 | DiseaseFree | 20.57 | LIVING | Female |
| TCGA-55-A492 | MX | N0 | Stage IA | T1a | 20 | DiseaseFree | 19.58 | LIVING | Female |
| TCGA-55-A493 | M0 | N0 | Stage IB | T2a | 0.9 | DiseaseFree | 0.92 | LIVING | Female |
| TCGA-55-A494 | MX | N0 | Stage IB | T2a | 16 | DiseaseFree | 15.8 | LIVING | Female |
| TCGA-55-A4DF | MX | N0 | Stage IA | T1b | 17 | Recurred/Progressed | 20.17 | DECEASED | Male |
| TCGA-55-A4DG | MX | N0 | Stage IA | T1b | 20 | DiseaseFree | 19.97 | LIVING | Male |
| TCGA-55-A57B | M0 | N0 | Stage IA | T1b | 18 | DiseaseFree | 17.94 | LIVING | Female |
| TCGA-62-8394 | M0 | N2 | Stage IIIB | T4 | NA | NA | 4.57 | DECEASED | Female |
| TCGA-62-8395 | M0 | N0 | Stage IIB | T3 | 16 | Recurred/Progressed | 39.95 | LIVING | Female |
| TCGA-62-8397 | M0 | N0 | Stage IIB | T3 | 42 | DiseaseFree | 42.35 | LIVING | Female |
| TCGA-62-8398 | M0 | N2 | Stage IIIA | T2 | NA | NA | 14.59 | DECEASED | Male |
| TCGA-62-8399 | M0 | N2 | Stage IIIA | T2 | 89 | DiseaseFree | 88.57 | LIVING | Male |
| TCGA-62-8402 | M0 | N2 | Stage IIIA | T2 | 25 | Recurred/Progressed | 49.21 | DECEASED | Female |
| TCGA-62-A46O | M0 | N0 | Stage IB | T2 | 31 | Recurred/Progressed | 47.77 | DECEASED | Female |
| TCGA-62-A46P | M0 | N0 | Stage IB | T2 | 8.8 | Recurred/Progressed | 19.51 | DECEASED | Male |
| TCGA-62-A46R | M0 | N0 | Stage IB | T2 | NA | NA | 56.67 | DECEASED | Female |
| TCGA-62-A46S | M0 | N0 | Stage IB | T2 | 17 | Recurred/Progressed | 54.3 | DECEASED | Male |
| TCGA-62-A46U | M0 | N1 | Stage IIB | T2 | 68 | DiseaseFree | 67.9 | LIVING | Female |
| TCGA-62-A46V | M0 | N0 | Stage IB | T2 | 72 | DiseaseFree | 72.24 | LIVING | Female |
| TCGA-62-A46Y | M0 | N2 | Stage IIIA | T2 | 10 | Recurred/Progressed | 13.6 | DECEASED | Female |
| TCGA-62-A470 | M0 | N0 | Stage IB | T2 | 18 | Recurred/Progressed | 39.22 | DECEASED | Male |
| TCGA-62-A471 | M0 | N1 | Stage IIB | T2b | 41 | DiseaseFree | 40.93 | LIVING | Male |
| TCGA-62-A472 | M0 | N0 | Stage IIB | T3 | 9.5 | Recurred/Progressed | 29.89 | LIVING | Male |
| TCGA-64-1676 | M0 | N0 | Stage IA | T1a | 57 | DiseaseFree | 56.77 | LIVING | Male |
| TCGA-64-1677 | M0 | N2 | Stage IIIA | T2 | 12 | Recurred/Progressed | 20.63 | DECEASED | Female |
| TCGA-64-1678 | M0 | N0 | NA | T2b | 39 | DiseaseFree | 39.06 | LIVING | Female |
| TCGA-64-1679 | M0 | N2 | Stage IIIA | T1 | 82 | DiseaseFree | 81.73 | LIVING | Female |
| TCGA-64-1680 | M1 | N2 | Stage IV | T2a | 37 | DiseaseFree | 36.99 | LIVING | Male |
| TCGA-64-1681 | M0 | N0 | Stage IA | T1 | 14 | Recurred/Progressed | 38.34 | DECEASED | Female |
| TCGA-64-5774 | M0 | N0 | Stage IB | T2 | 9.1 | Recurred/Progressed | 87.91 | LIVING | Male |
| TCGA-64-5775 | M0 | N0 | Stage IIIA | T4 | NA | NA | 2.04 | DECEASED | Male |
| TCGA-64-5778 | M0 | N0 | Stage IB | T2 | 38 | Recurred/Progressed | 42.87 | LIVING | Male |
| TCGA-64-5779 | M0 | N2 | Stage IIIA | T2 | 26 | Recurred/Progressed | 28.38 | LIVING | Male |
| TCGA-64-5781 | M0 | N0 | Stage IB | T2 | 3.2 | Recurred/Progressed | 51.22 | LIVING | Female |
| TCGA-64-5815 | M0 | N1 | Stage IIB | T2 | 28 | DiseaseFree | 28.45 | LIVING | Male |
| TCGA-67-3770 | M0 | N0 | Stage IA | T1 | 20 | DiseaseFree | 20.04 | LIVING | Female |
| TCGA-67-3771 | M0 | N0 | Stage IA | T1 | 20 | DiseaseFree | 20.04 | LIVING | Female |
| TCGA-67-3772 | M0 | N0 | Stage IB | T2 | 19 | DiseaseFree | 18.82 | LIVING | Female |
| TCGA-67-3773 | M0 | N0 | Stage IB | T2 | 14 | DiseaseFree | 14.03 | LIVING | Female |
| TCGA-67-3774 | M0 | N0 | Stage IB | T2 | 13 | DiseaseFree | 12.65 | LIVING | Female |
| TCGA-67-4679 | M0 | N0 | NA | T3 | 15 | DiseaseFree | 14.72 | LIVING | Male |
| TCGA-67-6215 | M0 | N0 | Stage IB | T2a | 5.7 | DiseaseFree | 5.72 | LIVING | Female |
| TCGA-67-6216 | M0 | N0 | Stage IA | T1a | 4.6 | DiseaseFree | 4.63 | LIVING | Female |
| TCGA-67-6217 | M0 | N1 | Stage IIA | T2a | 9.7 | Recurred/Progressed | 13.86 | LIVING | Female |
| TCGA-69-7760 | M0 | N0 | Stage IIB | T3 | 6.6 | DiseaseFree | 6.64 | LIVING | Male |
| TCGA-69-7761 | MX | N0 | Stage IB | T2a | 6.1 | DiseaseFree | 6.11 | LIVING | Male |
| TCGA-69-7763 | M0 | N0 | Stage IA | T1b | 23 | DiseaseFree | 22.67 | LIVING | Male |
| TCGA-69-7764 | M0 | N0 | Stage IA | T1b | 14 | DiseaseFree | 13.6 | LIVING | Male |
| TCGA-69-7765 | MX | N0 | NA | T4 | 5.4 | DiseaseFree | 5.42 | LIVING | Male |
| TCGA-69-7973 | M0 | N0 | Stage IB | T2a | 7.6 | DiseaseFree | 7.56 | LIVING | Female |
| TCGA-69-7974 | MX | N2 | Stage IIIA | T2a | 6 | DiseaseFree | 6.04 | LIVING | Female |
| TCGA-69-7978 | MX | N1 | Stage IIB | T2b | 4.4 | DiseaseFree | 4.4 | LIVING | Male |
| TCGA-69-7979 | MX | N0 | Stage IB | T2a | 13 | DiseaseFree | 13.4 | LIVING | Female |
| TCGA-69-7980 | M0 | N0 | Stage I | T1b | 14 | DiseaseFree | 13.5 | LIVING | Female |
| TCGA-69-8253 | MX | N1 | Stage IIA | T1a | 14 | DiseaseFree | 13.99 | LIVING | Female |
| TCGA-69-8254 | NA | NA | NA | T2b | 13 | DiseaseFree | 13.44 | LIVING | Male |
| TCGA-69-8255 | M0 | N0 | Stage IA | T1a | 4.2 | DiseaseFree | 4.24 | LIVING | Male |
| TCGA-69-8453 | MX | N0 | Stage IIB | T3 | 11 | Recurred/Progressed | 26.71 | LIVING | Male |
| TCGA-69-A59K | M0 | N0 | Stage IIB | T3 | 19 | DiseaseFree | 19.42 | LIVING | Female |
| TCGA-71-6725 | M0 | N0 | Stage IB | T2 | 5.4 | Recurred/Progressed | 8.41 | LIVING | Female |
| TCGA-71-8520 | M0 | N0 | Stage IB | T2 | 5.9 | Recurred/Progressed | 6.9 | DECEASED | Female |
| TCGA-73-4658 | M0 | N0 | Stage IB | T2 | NA | NA | 52.56 | DECEASED | Female |
| TCGA-73-4659 | M0 | N2 | Stage IIIA | T2 | 10 | Recurred/Progressed | 23.36 | DECEASED | Male |
| TCGA-73-4662 | M0 | N0 | Stage IA | T1 | 0.7 | Recurred/Progressed | 82.62 | LIVING | Female |
| TCGA-73-4666 | M1 | N0 | Stage IV | T1 | 26 | DiseaseFree | 26.28 | LIVING | Female |
| TCGA-73-4668 | M0 | N1 | Stage IIB | T2 | 9.3 | Recurred/Progressed | 15.34 | LIVING | Female |
| TCGA-73-4670 | M1 | N0 | Stage IV | T2 | 4.3 | DiseaseFree | 4.3 | LIVING | Female |
| TCGA-73-4675 | M0 | N1 | Stage IIIA | T3 | 12 | Recurred/Progressed | 30.29 | DECEASED | Male |
| TCGA-73-4676 | M0 | N1 | Stage IIA | T2a | NA | NA | 9.23 | DECEASED | Male |
| TCGA-73-4677 | M0 | N0 | NA | T2a | NA | NA | 1.25 | DECEASED | Male |
| TCGA-73-7498 | M0 | N0 | Stage IA | T1b | 39 | DiseaseFree | 39.06 | LIVING | Female |
| TCGA-73-7499 | M0 | N0 | Stage IB | T2a | 48 | Recurred/Progressed | 50.3 | DECEASED | Female |
| TCGA-73-A9RS | M0 | N0 | Stage IIB | T3 | 3.5 | Recurred/Progressed | 11.17 | DECEASED | Male |
| TCGA-75-5122 | M0 | N0 | Stage IB | T2 | NA | NA | NA | DECEASED | Male |
| TCGA-75-5125 | M0 | N1 | Stage IIB | T2 | 58 | Recurred/Progressed | 66.59 | DECEASED | Male |
| TCGA-75-5126 | M0 | N2 | Stage IIIA | T3 | NA | DiseaseFree | NA | LIVING | Female |
| TCGA-75-5146 | M0 | N0 | Stage IB | T2 | 58 | Recurred/Progressed | 77.79 | LIVING | Male |
| TCGA-75-5147 | M0 | N0 | Stage IB | T2 | 44 | DiseaseFree | 43.79 | LIVING | Female |
| TCGA-75-6203 | M0 | N2 | Stage IIIA | T2 | NA | DiseaseFree | NA | LIVING | Female |
| TCGA-75-6205 | M0 | N0 | Stage IB | T2a | NA | NA | NA | DECEASED | Female |
| TCGA-75-6206 | M0 | N0 | Stage IB | T2 | 85 | DiseaseFree | 85.09 | LIVING | Male |
| TCGA-75-6207 | M0 | N2 | Stage IIIA | T2 | NA | NA | NA | DECEASED | Male |
| TCGA-75-6211 | M0 | N0 | Stage IB | T2 | NA | NA | NA | DECEASED | Female |
| TCGA-75-6212 | M0 | N1 | Stage IIB | T2 | 44 | Recurred/Progressed | 49.8 | DECEASED | Female |
| TCGA-75-6214 | M0 | N2 | Stage IIIA | T2 | 14 | Recurred/Progressed | 36.63 | DECEASED | Female |
| TCGA-75-7025 | M0 | N0 | Stage IB | T2 | 49 | Recurred/Progressed | 108.57 | LIVING | Male |
| TCGA-75-7027 | M0 | N0 | Stage IB | T2 | 97 | Recurred/Progressed | 100.49 | LIVING | Male |
| TCGA-75-7030 | M0 | N0 | Stage IIB | T3 | NA | DiseaseFree | NA | LIVING | Male |
| TCGA-75-7031 | M0 | N0 | Stage IB | T2 | NA | DiseaseFree | NA | LIVING | Female |
| TCGA-78-7143 | M0 | N0 | Stage IB | T2 | 151 | Recurred/Progressed | 162.98 | DECEASED | Female |
| TCGA-78-7145 | M1 | N1 | Stage IV | T4 | 14 | Recurred/Progressed | 27.14 | DECEASED | Female |
| TCGA-78-7146 | M0 | N2 | Stage IIIA | T2 | NA | NA | 5.68 | DECEASED | Female |
| TCGA-78-7147 | M0 | N1 | Stage IIB | T2 | 19 | Recurred/Progressed | 19.25 | DECEASED | Female |
| TCGA-78-7148 | M0 | N1 | Stage IIB | T2 | 6 | Recurred/Progressed | 20.57 | DECEASED | Male |
| TCGA-78-7149 | M0 | N0 | Stage IIIB | T4 | 129 | DiseaseFree | 129.43 | LIVING | Male |
| TCGA-78-7150 | M0 | N1 | Stage IIB | T2 | 4.6 | Recurred/Progressed | 21.88 | DECEASED | Male |
| TCGA-78-7152 | M0 | N0 | Stage IB | T2 | 39 | Recurred/Progressed | 39.91 | DECEASED | Male |
| TCGA-78-7153 | M0 | N0 | Stage IB | T2 | 119 | DiseaseFree | 119.42 | LIVING | Female |
| TCGA-78-7154 | M0 | N2 | Stage IIIA | T3 | NA | NA | 19.48 | DECEASED | Male |
| TCGA-78-7155 | M0 | N0 | Stage IB | T2 | 6.9 | Recurred/Progressed | 38.47 | DECEASED | Male |
| TCGA-78-7156 | M1 | N1 | Stage IV | T4 | NA | NA | 32.06 | DECEASED | Male |
| TCGA-78-7158 | M0 | N2 | Stage IIIB | T4 | 4.3 | Recurred/Progressed | 5.88 | DECEASED | Female |
| TCGA-78-7159 | M0 | NX | Stage IA | T1 | 65 | DiseaseFree | 64.85 | LIVING | Female |
| TCGA-78-7160 | M1 | N2 | Stage IV | T4 | NA | NA | 22.9 | DECEASED | Male |
| TCGA-78-7161 | M0 | N0 | Stage IIB | T3 | 5.3 | Recurred/Progressed | 9.56 | DECEASED | Female |
| TCGA-78-7162 | M0 | N0 | Stage IA | T1 | 90 | Recurred/Progressed | 104.11 | DECEASED | Male |
| TCGA-78-7163 | M0 | N0 | Stage IB | T2 | 238 | DiseaseFree | 238.11 | LIVING | Male |
| TCGA-78-7166 | M0 | N1 | Stage IIB | T2 | NA | NA | 8.48 | DECEASED | Male |
| TCGA-78-7167 | M1 | N0 | Stage IV | T2 | 24 | Recurred/Progressed | 88.07 | DECEASED | Male |
| TCGA-78-7220 | M0 | N2 | Stage IIIA | T2 | 17 | Recurred/Progressed | 26.51 | DECEASED | Female |
| TCGA-78-7535 | M0 | N0 | Stage IB | T2 | 27 | Recurred/Progressed | 31.18 | DECEASED | Male |
| TCGA-78-7536 | M0 | N2 | Stage IIIA | T2 | 7.6 | Recurred/Progressed | 8.02 | DECEASED | Male |
| TCGA-78-7537 | M0 | N0 | Stage IB | T2 | NA | NA | 53.29 | DECEASED | Male |
| TCGA-78-7539 | M0 | N0 | Stage IIA | T2b | 23 | Recurred/Progressed | 25.99 | LIVING | Female |
| TCGA-78-7540 | M0 | N0 | Stage IB | T2 | NA | NA | 39.32 | DECEASED | Female |
| TCGA-78-7542 | M0 | N0 | Stage IB | T2 | NA | NA | 10.55 | DECEASED | Male |
| TCGA-78-7633 | M0 | N0 | Stage IB | T2 | 48 | Recurred/Progressed | 50.2 | DECEASED | Male |
| TCGA-78-8640 | M0 | N1 | Stage IIA | T1 | 232 | DiseaseFree | 232 | LIVING | Male |
| TCGA-78-8648 | M0 | N0 | Stage IIB | T3 | 24 | Recurred/Progressed | 39.72 | DECEASED | Female |
| TCGA-78-8655 | M0 | N0 | Stage IA | T1 | 78 | DiseaseFree | 77.53 | LIVING | Female |
| TCGA-78-8660 | M0 | N1 | Stage IIB | T2 | 8 | Recurred/Progressed | 10.55 | DECEASED | Male |
| TCGA-78-8662 | M0 | N0 | Stage IB | T2 | 100 | Recurred/Progressed | 110.41 | DECEASED | Female |
| TCGA-80-5607 | M0 | N1 | Stage IIB | T2 | NA | DiseaseFree | NA | LIVING | Female |
| TCGA-80-5608 | M0 | N0 | Stage IA | T1 | 93 | DiseaseFree | 93.04 | LIVING | Female |
| TCGA-80-5611 | M0 | N0 | Stage IB | T2 | 85 | DiseaseFree | 85.25 | LIVING | Male |
| TCGA-83-5908 | M0 | N0 | Stage IA | T1 | 27 | DiseaseFree | 27.07 | LIVING | Female |
| TCGA-86-6562 | M0 | N1 | Stage IIA | T2a | 9 | Recurred/Progressed | 12.35 | DECEASED | Male |
| TCGA-86-6851 | M0 | N1 | Stage IIA | T1b | 5.9 | DiseaseFree | 5.88 | LIVING | Female |
| TCGA-86-7701 | M1 | N0 | Stage IV | T2 | 30 | Recurred/Progressed | 31.11 | LIVING | Male |
| TCGA-86-7711 | M0 | N1 | Stage IIA | T2a | NA | NA | 34.36 | DECEASED | Male |
| TCGA-86-7713 | M0 | N0 | Stage IIA | T2b | 38 | DiseaseFree | 38.01 | LIVING | Male |
| TCGA-86-7714 | M0 | N2 | Stage IIIA | T1b | NA | NA | 20.53 | DECEASED | Female |
| TCGA-86-7953 | M0 | N0 | Stage IA | T1b | 33 | DiseaseFree | 32.75 | LIVING | Female |
| TCGA-86-7954 | M0 | N0 | Stage IB | T2 | 20 | DiseaseFree | 19.88 | LIVING | Female |
| TCGA-86-7955 | M0 | N0 | Stage IB | T2a | 27 | Recurred/Progressed | 35.22 | LIVING | Male |
| TCGA-86-8054 | M0 | N1 | Stage IIB | T2b | 38 | DiseaseFree | 37.71 | LIVING | Male |
| TCGA-86-8055 | M0 | N1 | Stage IIA | T2a | NA | NA | 4.07 | DECEASED | Male |
| TCGA-86-8056 | M0 | N0 | Stage IIIA | T4 | 4.6 | DiseaseFree | 4.57 | LIVING | Female |
| TCGA-86-8073 | M0 | N0 | Stage IB | T2a | 24 | DiseaseFree | 24.31 | LIVING | Male |
| TCGA-86-8074 | M0 | N1 | Stage IIA | T1b | 0.8 | DiseaseFree | 0.79 | LIVING | Female |
| TCGA-86-8075 | M0 | N0 | Stage IB | T2 | 6.5 | Recurred/Progressed | 22.8 | DECEASED | Female |
| TCGA-86-8076 | M0 | N0 | Stage IA | T1 | 33 | DiseaseFree | 32.62 | LIVING | Male |
| TCGA-86-8278 | M0 | N1 | Stage IIB | T2 | 1 | Recurred/Progressed | 31.01 | LIVING | Female |
| TCGA-86-8279 | M0 | N1 | Stage IIA | T2a | 31 | DiseaseFree | 31.18 | LIVING | Male |
| TCGA-86-8280 | M0 | N0 | Stage IIA | T2b | 23 | DiseaseFree | 23.03 | LIVING | Female |
| TCGA-86-8281 | M0 | NX | Stage IA | T1 | 0 | DiseaseFree | 0 | LIVING | Male |
| TCGA-86-8358 | M0 | N0 | Stage IB | T2a | 21 | DiseaseFree | 21.45 | LIVING | Male |
| TCGA-86-8359 | M0 | N2 | Stage IIIA | T3 | NA | NA | 14.59 | DECEASED | Male |
| TCGA-86-8585 | M0 | N0 | Stage IB | T2a | 12 | DiseaseFree | 11.6 | LIVING | Male |
| TCGA-86-8668 | M0 | N0 | Stage IA | T1b | 14 | DiseaseFree | 13.9 | LIVING | Female |
| TCGA-86-8669 | M0 | N0 | Stage IA | T1b | 28 | Recurred/Progressed | 30.81 | LIVING | Male |
| TCGA-86-8671 | M0 | N1 | Stage IIB | T2b | 28 | DiseaseFree | 27.56 | LIVING | Female |
| TCGA-86-8672 | M0 | N0 | Stage IIB | T3 | NA | NA | 0.62 | DECEASED | Male |
| TCGA-86-8673 | M0 | N0 | Stage IB | T2 | 21 | Recurred/Progressed | 28.32 | LIVING | Male |
| TCGA-86-8674 | M0 | N1 | Stage IIA | T2a | 11 | Recurred/Progressed | 26.48 | LIVING | Male |
| TCGA-86-A456 | M0 | N0 | Stage IA | T1a | 29 | DiseaseFree | 29.43 | LIVING | Female |
| TCGA-86-A4D0 | M0 | N0 | Stage IIA | T2b | NA | NA | 3.81 | DECEASED | Male |
| TCGA-86-A4JF | M0 | N0 | Stage IIB | T3 | 14 | Recurred/Progressed | 24.21 | DECEASED | Male |
| TCGA-86-A4P7 | M0 | N0 | Stage IB | T2a | 14 | DiseaseFree | 13.63 | LIVING | Female |
| TCGA-86-A4P8 | MX | N2 | Stage IIIA | T1b | 26 | DiseaseFree | 26.45 | LIVING | Female |
| TCGA-91-6828 | M0 | N0 | Stage IA | T1a | 11 | DiseaseFree | 10.61 | LIVING | Male |
| TCGA-91-6829 | MX | N0 | Stage IB | T2 | NA | NA | 41.33 | DECEASED | Male |
| TCGA-91-6830 | MX | N1 | Stage IIA | T1 | 0.6 | Recurred/Progressed | 1.97 | LIVING | Female |
| TCGA-91-6831 | MX | N0 | Stage IB | T2 | 10 | DiseaseFree | 10.18 | LIVING | Male |
| TCGA-91-6835 | M0 | N0 | Stage IA | T1 | 2.6 | DiseaseFree | 2.6 | LIVING | Female |
| TCGA-91-6836 | MX | N0 | Stage IB | T2 | 14 | DiseaseFree | 13.7 | LIVING | Female |
| TCGA-91-6840 | M0 | N0 | Stage IA | T1b | 12 | DiseaseFree | 12.22 | LIVING | Female |
| TCGA-91-6847 | MX | N0 | Stage IB | T2 | 25 | Recurred/Progressed | 27.66 | LIVING | Female |
| TCGA-91-6848 | MX | N2 | Stage IIIA | T2 | 7.4 | DiseaseFree | 7.36 | LIVING | Male |
| TCGA-91-6849 | MX | N2 | Stage IIIA | T2 | 1.2 | DiseaseFree | 1.15 | LIVING | Female |
| TCGA-91-7771 | MX | N0 | Stage IIB | T3 | 16 | DiseaseFree | 16.16 | LIVING | Male |
| TCGA-91-8496 | MX | NX | Stage IB | T2a | 17 | DiseaseFree | 16.59 | LIVING | Female |
| TCGA-91-8497 | MX | N0 | Stage IA | T1a | NA | NA | 14.26 | DECEASED | Female |
| TCGA-91-8499 | MX | N0 | Stage IA | T1b | 1.2 | DiseaseFree | 1.18 | LIVING | Female |
| TCGA-91-A4BC | MX | N0 | Stage IIA | T2b | 1.5 | DiseaseFree | 1.45 | LIVING | Male |
| TCGA-91-A4BD | MX | N1 | Stage IIA | T1b | 20 | DiseaseFree | 19.81 | LIVING | Male |
| TCGA-93-7347 | MX | N0 | Stage IA | T1a | 22 | DiseaseFree | 22.44 | LIVING | Female |
| TCGA-93-7348 | MX | N0 | Stage IA | T1a | 17 | DiseaseFree | 17.44 | LIVING | Female |
| TCGA-93-8067 | MX | N0 | Stage IB | T2a | 6.1 | DiseaseFree | 6.11 | LIVING | Male |
| TCGA-93-A4JN | M1a | N0 | Stage IV | T2a | 24 | DiseaseFree | 23.59 | LIVING | Male |
| TCGA-93-A4JO | MX | N0 | Stage IA | T1a | NA | NA | 1.08 | DECEASED | Male |
| TCGA-93-A4JP | M1b | NX | Stage IV | TX | 16 | Recurred/Progressed | 18.99 | LIVING | Male |
| TCGA-93-A4JQ | MX | N0 | Stage IA | T1b | 17 | DiseaseFree | 17.28 | LIVING | Male |
| TCGA-95-7039 | MX | N0 | Stage IIB | T3 | 41 | Recurred/Progressed | 41.79 | LIVING | Female |
| TCGA-95-7043 | MX | N0 | Stage IA | T1a | NA | NA | 16.52 | DECEASED | Female |
| TCGA-95-7562 | M0 | N1 | Stage IIA | T2a | NA | NA | 2.86 | DECEASED | Male |
| TCGA-95-7567 | M0 | N1 | Stage IIB | T2b | 19 | DiseaseFree | 18.66 | LIVING | Male |
| TCGA-95-7944 | M0 | N0 | Stage IA | T1a | 12 | DiseaseFree | 12.39 | LIVING | Male |
| TCGA-95-7947 | M0 | N0 | Stage IA | T1a | 16 | DiseaseFree | 15.67 | LIVING | Male |
| TCGA-95-7948 | M0 | N0 | Stage IB | T2a | 16 | DiseaseFree | 15.64 | LIVING | Female |
| TCGA-95-8039 | MX | N0 | Stage IA | T1 | 7.5 | Recurred/Progressed | 27.27 | LIVING | Male |
| TCGA-95-8494 | M0 | N1 | Stage IIA | T2a | 2.8 | DiseaseFree | 2.76 | LIVING | Male |
| TCGA-95-A4VK | M0 | N2 | Stage IIIA | T2b | 16 | Recurred/Progressed | 21.39 | LIVING | Female |
| TCGA-95-A4VN | M0 | N1 | Stage IIA | T2a | 18 | DiseaseFree | 18.17 | LIVING | Female |
| TCGA-95-A4VP | M0 | N2 | Stage IIIA | T2b | 7.1 | Recurred/Progressed | 19.88 | LIVING | Female |
| TCGA-97-7546 | MX | N0 | Stage IA | T1 | 41 | Recurred/Progressed | 42.21 | LIVING | Female |
| TCGA-97-7547 | MX | N0 | Stage IB | T2 | 38 | Recurred/Progressed | 64.55 | LIVING | Female |
| TCGA-97-7552 | MX | N0 | Stage IB | T2 | 26 | Recurred/Progressed | 63.47 | LIVING | Male |
| TCGA-97-7553 | MX | N0 | Stage IA | T1 | 61 | DiseaseFree | 61.43 | LIVING | Female |
| TCGA-97-7554 | M0 | N2 | Stage IIIA | T2a | 25 | DiseaseFree | 25.46 | LIVING | Female |
| TCGA-97-7937 | MX | N0 | Stage IB | T2a | 19 | DiseaseFree | 18.53 | LIVING | Male |
| TCGA-97-7938 | MX | N0 | Stage IA | T1a | NA | NA | 0.59 | DECEASED | Female |
| TCGA-97-7941 | MX | N0 | Stage IA | T1b | 16 | DiseaseFree | 15.9 | LIVING | Female |
| TCGA-97-8171 | M1a | N2 | Stage IV | T2a | 14 | Recurred/Progressed | 18.66 | LIVING | Male |
| TCGA-97-8172 | M0 | N0 | Stage IB | T2a | 18 | DiseaseFree | 17.9 | LIVING | Female |
| TCGA-97-8174 | M0 | N0 | Stage IIA | T2b | NA | NA | 5.39 | DECEASED | Male |
| TCGA-97-8175 | M0 | N0 | Stage IB | T2a | 9.7 | Recurred/Progressed | 18.1 | LIVING | Female |
| TCGA-97-8176 | M0 | N1 | Stage IIIA | T3 | 1.3 | Recurred/Progressed | 15.37 | DECEASED | Male |
| TCGA-97-8177 | M0 | N0 | Stage IB | T2a | 16 | DiseaseFree | 16.39 | LIVING | Female |
| TCGA-97-8179 | M0 | N0 | Stage IA | T1a | 14 | DiseaseFree | 14.29 | LIVING | Male |
| TCGA-97-8547 | MX | N2 | Stage IIIA | T2a | 22 | DiseaseFree | 21.58 | LIVING | Female |
| TCGA-97-8552 | MX | N0 | Stage I | T1a | 21 | DiseaseFree | 20.57 | LIVING | Female |
| TCGA-97-A4LX | M0 | N0 | Stage IB | T2a | 20 | DiseaseFree | 20.17 | LIVING | Male |
| TCGA-97-A4M0 | M0 | N0 | Stage IB | T2a | 21 | DiseaseFree | 21.42 | LIVING | Female |
| TCGA-97-A4M1 | M0 | N0 | Stage IA | T1a | 20 | DiseaseFree | 19.74 | LIVING | Female |
| TCGA-97-A4M2 | M0 | N0 | Stage IA | T1a | 21 | DiseaseFree | 20.5 | LIVING | Male |
| TCGA-97-A4M3 | M0 | N0 | Stage IA | T1b | 1.8 | Recurred/Progressed | 17.74 | LIVING | Female |
| TCGA-97-A4M5 | M0 | N0 | Stage IA | T1b | 21 | DiseaseFree | 20.83 | LIVING | Male |
| TCGA-97-A4M6 | M0 | N0 | Stage IA | T1a | 19 | DiseaseFree | 18.66 | LIVING | Female |
| TCGA-97-A4M7 | M0 | N0 | Stage IA | T1b | 21 | DiseaseFree | 20.66 | LIVING | Male |
| TCGA-99-7458 | M0 | N0 | Stage IIIA | T4 | 25 | DiseaseFree | 24.54 | LIVING | Female |
| TCGA-99-8025 | M0 | N2 | Stage IIIA | T3 | 35 | DiseaseFree | 34.82 | LIVING | Female |
| TCGA-99-8028 | M0 | N0 | Stage IA | T1a | 37 | DiseaseFree | 36.73 | LIVING | Female |
| TCGA-99-8032 | M0 | N0 | Stage IA | T1a | 1.5 | DiseaseFree | 1.45 | LIVING | Male |
| TCGA-99-8033 | M1 | NX | Stage IV | TX | NA | NA | 21.55 | DECEASED | Female |
| TCGA-99-AA5R | M0 | N0 | Stage IA | T1a | 22 | DiseaseFree | 21.62 | LIVING | Female |
| TCGA-J2-8192 | MX | N1 | Stage IIA | T2a | 16 | Recurred/Progressed | 24.28 | LIVING | Female |
| TCGA-J2-8194 | MX | N0 | Stage IIB | T3 | 15 | Recurred/Progressed | 23.78 | LIVING | Female |
| TCGA-J2-A4AD | MX | N0 | Stage IA | T1a | 17 | Recurred/Progressed | 18.07 | DECEASED | Female |
| TCGA-J2-A4AE | MX | N0 | Stage IA | T1a | 35 | DiseaseFree | 35.45 | LIVING | Female |
| TCGA-J2-A4AG | MX | N0 | Stage IA | T1b | 32 | DiseaseFree | 32.46 | LIVING | Female |
| TCGA-L4-A4E5 | M0 | N0 | Stage I | T1 | 19 | DiseaseFree | 18.99 | LIVING | Female |
| TCGA-L4-A4E6 | M0 | N0 | Stage IA | T1 | 14 | DiseaseFree | 14.29 | LIVING | Male |
| TCGA-L9-A443 | MX | N0 | Stage IA | T1a | NA | NA | 6.34 | DECEASED | Female |
| TCGA-L9-A444 | MX | N0 | Stage IA | T1a | 10 | DiseaseFree | 10.09 | LIVING | Female |
| TCGA-L9-A50W | MX | N1 | Stage IIA | T1b | 13 | Recurred/Progressed | 14.52 | DECEASED | Male |
| TCGA-L9-A5IP | M1b | N2 | Stage IV | T3 | 1.6 | Recurred/Progressed | 1.91 | DECEASED | Female |
| TCGA-L9-A743 | M0 | N1 | Stage IIA | T2a | 22 | DiseaseFree | 21.81 | LIVING | Male |
| TCGA-L9-A7SV | M0 | N1 | Stage IIA | T2a | 19 | DiseaseFree | 18.56 | LIVING | Male |
| TCGA-L9-A8F4 | MX | N0 | Stage IB | T2a | 16 | DiseaseFree | 15.64 | LIVING | Female |
| TCGA-MN-A4N1 | M0 | N1 | Stage IIA | T2a | 27 | DiseaseFree | 27.17 | LIVING | Male |
| TCGA-MN-A4N4 | M0 | N0 | Stage IA | T1b | 39 | DiseaseFree | 38.6 | LIVING | Male |
| TCGA-MN-A4N5 | M0 | N0 | Stage IA | T1a | 2.8 | DiseaseFree | 2.76 | LIVING | Male |
| TCGA-MP-A4SV | M0 | N0 | Stage IB | T2 | NA | NA | 86.07 | DECEASED | Male |
| TCGA-MP-A4SW | M0 | N1 | Stage IIB | T2 | NA | NA | 58.41 | DECEASED | Male |
| TCGA-MP-A4SY | M0 | N1 | Stage IIB | T2 | 16 | Recurred/Progressed | 49.31 | DECEASED | Male |
| TCGA-MP-A4T4 | M0 | N1 | Stage IIB | T2 | NA | NA | 85.97 | DECEASED | Female |
| TCGA-MP-A4T6 | MX | N2 | Stage IIIA | T1 | NA | NA | 58.8 | DECEASED | Female |
| TCGA-MP-A4T7 | M1 | N0 | Stage IV | T2 | NA | NA | 5.49 | DECEASED | Female |
| TCGA-MP-A4T8 | M0 | N2 | Stage IIIA | T2 | NA | NA | 5.29 | DECEASED | Male |
| TCGA-MP-A4T9 | MX | N2 | Stage IIIA | T2 | 41 | Recurred/Progressed | 41.56 | DECEASED | Female |
| TCGA-MP-A4TA | M0 | N0 | Stage IA | T1 | 24 | Recurred/Progressed | 31.21 | DECEASED | Female |
| TCGA-MP-A4TC | M0 | N2 | Stage IIIA | T1 | NA | NA | 2.43 | DECEASED | Male |
| TCGA-MP-A4TD | M0 | N2 | Stage IIIA | T2 | 7.4 | Recurred/Progressed | 10.09 | DECEASED | Male |
| TCGA-MP-A4TE | MX | N0 | Stage IIA | T2b | 28 | Recurred/Progressed | 29.43 | DECEASED | Male |
| TCGA-MP-A4TF | M0 | N0 | Stage IIA | T2b | 6.4 | Recurred/Progressed | 11.04 | DECEASED | Female |
| TCGA-MP-A4TH | M0 | N0 | Stage IA | T1a | 24 | DiseaseFree | 24.34 | LIVING | Female |
| TCGA-MP-A4TI | M0 | N1 | Stage IIA | T2a | 2.8 | Recurred/Progressed | 14.09 | DECEASED | Male |
| TCGA-MP-A4TJ | M0 | N0 | Stage IA | T1 | NA | NA | 11.14 | DECEASED | Female |
| TCGA-MP-A4TK | MX | N1 | Stage IIB | T2 | 18 | Recurred/Progressed | 19.12 | DECEASED | Female |
| TCGA-MP-A5C7 | M0 | N0 | Stage IB | T2 | 74 | DiseaseFree | 73.85 | LIVING | Female |
| TCGA-NJ-A4YF | M0 | N0 | Stage IA | T1 | 71 | DiseaseFree | 70.99 | LIVING | Female |
| TCGA-NJ-A4YG | M0 | N0 | Stage IB | T2 | 74 | DiseaseFree | 74.28 | LIVING | Male |
| TCGA-NJ-A4YI | M0 | N2 | Stage IIIA | T2 | NA | NA | 0.13 | DECEASED | Female |
| TCGA-NJ-A4YP | M0 | N0 | Stage IB | T2a | 1.6 | DiseaseFree | 1.64 | LIVING | Male |
| TCGA-NJ-A4YQ | M0 | N0 | Stage IA | T1b | 47 | DiseaseFree | 47.04 | LIVING | Female |
| TCGA-NJ-A55A | M0 | N0 | Stage IB | T2 | 0.5 | DiseaseFree | 0.49 | LIVING | Female |
| TCGA-NJ-A55O | M0 | N1 | Stage IIA | T1b | 0.4 | DiseaseFree | 0.43 | LIVING | Female |
| TCGA-NJ-A55R | MX | N0 | Stage IA | T1b | 20 | DiseaseFree | 19.81 | LIVING | Male |
| TCGA-NJ-A7XG | M0 | N1 | Stage IIIA | T4 | 20 | DiseaseFree | 20.27 | LIVING | Male |
| TCGA-O1-A52J | MX | N0 | Stage IA | T1 | 30 | Recurred/Progressed | 59.07 | DECEASED | Female |
| TCGA-S2-AA1A | M0 | N0 | Stage IA | T1b | 17 | DiseaseFree | 16.85 | LIVING | Female |
