# Supplementary material for: TRIM28 is a distinct prognostic biomarker that worsens the tumor immune microenvironment in lung adenocarcinoma
Source: Aging (Albany NY). 2020 Oct 22;12(20):20308–31. doi: 10.18632/aging.103804 (PMC7655206; doi:10.18632/aging.103804)
Supplement: Supplementary Table 2 [file aging-12-103804-s003..docx]

Supplementary Table 2. The immune and stromal scores of 517 LUAD patients.

| ID | Stromal_score | Immune_score | ESTIMATE_score |
| --- | --- | --- | --- |
| TCGA-05-4244-01 | -470.99 | 933.74 | 462.76 |
| TCGA-05-4249-01 | -0.14 | 1033.35 | 1033.21 |
| TCGA-05-4250-01 | 263.1 | 1184.84 | 1447.95 |
| TCGA-05-4382-01 | 1205.42 | 1641.81 | 2847.24 |
| TCGA-05-4384-01 | -75.43 | 767.77 | 692.34 |
| TCGA-05-4389-01 | -397.74 | 1456.58 | 1058.84 |
| TCGA-05-4390-01 | 124.48 | 76.91 | 201.39 |
| TCGA-05-4395-01 | -620.75 | 854.14 | 233.39 |
| TCGA-05-4396-01 | -714.18 | -204.96 | -919.14 |
| TCGA-05-4397-01 | -688.16 | 463.66 | -224.49 |
| TCGA-05-4398-01 | 362.65 | 1514.83 | 1877.48 |
| TCGA-05-4402-01 | 208.5 | 848.38 | 1056.88 |
| TCGA-05-4403-01 | 92.1 | 1327.2 | 1419.3 |
| TCGA-05-4405-01 | 873.93 | 845.61 | 1719.54 |
| TCGA-05-4410-01 | -103.47 | 1449.18 | 1345.71 |
| TCGA-05-4415-01 | -1124.07 | 52.89 | -1071.18 |
| TCGA-05-4417-01 | 1292.02 | 1580.98 | 2873 |
| TCGA-05-4418-01 | -111.7 | 1233.33 | 1121.62 |
| TCGA-05-4420-01 | -421.95 | -179.48 | -601.43 |
| TCGA-05-4422-01 | -693 | 1425.83 | 732.83 |
| TCGA-05-4424-01 | 509.42 | 1169.53 | 1678.95 |
| TCGA-05-4425-01 | 440.03 | 1600.66 | 2040.69 |
| TCGA-05-4426-01 | -590.25 | 695.94 | 105.7 |
| TCGA-05-4427-01 | 77.18 | 823.37 | 900.55 |
| TCGA-05-4430-01 | 1057.05 | 1304.67 | 2361.71 |
| TCGA-05-4432-01 | -140.24 | 822.09 | 681.85 |
| TCGA-05-4433-01 | -305.36 | 1499.08 | 1193.72 |
| TCGA-05-4434-01 | 617.81 | 1881.89 | 2499.7 |
| TCGA-05-5420-01 | 644.13 | 2704.93 | 3349.06 |
| TCGA-05-5423-01 | 90.11 | 1820.79 | 1910.9 |
| TCGA-05-5425-01 | 165.33 | 1753.64 | 1918.97 |
| TCGA-05-5428-01 | 93.84 | 831.02 | 924.87 |
| TCGA-05-5429-01 | -495.96 | -66.98 | -562.94 |
| TCGA-05-5715-01 | 457.88 | 944.86 | 1402.74 |
| TCGA-35-3615-01 | -422.99 | 405.82 | -17.17 |
| TCGA-35-4122-01 | 744.23 | 2295.05 | 3039.28 |
| TCGA-35-4123-01 | 622.04 | 2138.23 | 2760.27 |
| TCGA-35-5375-01 | -225.2 | 1039.07 | 813.87 |
| TCGA-38-4625-01 | -138.22 | 964.93 | 826.71 |
| TCGA-38-4626-01 | 1082.46 | 1926.47 | 3008.93 |
| TCGA-38-4627-01 | 2098.77 | 1512.22 | 3610.98 |
| TCGA-38-4628-01 | 336.32 | 720.64 | 1056.96 |
| TCGA-38-4629-01 | 918.97 | 1883.89 | 2802.86 |
| TCGA-38-4630-01 | -412.46 | -361.37 | -773.82 |
| TCGA-38-4631-01 | -828.13 | 254.82 | -573.31 |
| TCGA-38-4632-01 | 60.86 | 1531.5 | 1592.36 |
| TCGA-38-6178-01 | 305.45 | 409.24 | 714.69 |
| TCGA-38-7271-01 | 1191.36 | 2743.79 | 3935.15 |
| TCGA-38-A44F-01 | 426.69 | 1875.62 | 2302.31 |
| TCGA-44-2655-01 | -437.38 | 861.14 | 423.77 |
| TCGA-44-2656-01 | 731.06 | 2175.91 | 2906.97 |
| TCGA-44-2657-01 | 679.86 | 2387.9 | 3067.76 |
| TCGA-44-2659-01 | 641.16 | 1273.63 | 1914.79 |
| TCGA-44-2661-01 | 1019.75 | 2577.1 | 3596.85 |
| TCGA-44-2662-01 | 819.69 | 1702.24 | 2521.93 |
| TCGA-44-2665-01 | 1496.26 | 1324.81 | 2821.06 |
| TCGA-44-2666-01 | -715.25 | 135.45 | -579.79 |
| TCGA-44-2668-01 | 959.18 | 1861.47 | 2820.64 |
| TCGA-44-3396-01 | 1133.45 | 1885.83 | 3019.28 |
| TCGA-44-3398-01 | 1230.85 | 1896.59 | 3127.44 |
| TCGA-44-3918-01 | 1092.2 | 2304 | 3396.2 |
| TCGA-44-3919-01 | 452.54 | 1640.92 | 2093.46 |
| TCGA-44-4112-01 | 543.91 | 626.49 | 1170.4 |
| TCGA-44-5643-01 | -1181.08 | 412.41 | -768.67 |
| TCGA-44-5644-01 | -1149.84 | -640.82 | -1790.66 |
| TCGA-44-5645-01 | 204.95 | 1680.22 | 1885.17 |
| TCGA-44-6145-01 | 686.36 | 1890.66 | 2577.02 |
| TCGA-44-6146-01 | -944.86 | -208.62 | -1153.48 |
| TCGA-44-6147-01 | 552.01 | 1026.48 | 1578.49 |
| TCGA-44-6148-01 | 452.62 | 689.74 | 1142.35 |
| TCGA-44-6774-01 | 1888.92 | 1020.11 | 2909.03 |
| TCGA-44-6775-01 | 1357.55 | 1481.59 | 2839.15 |
| TCGA-44-6776-01 | -897.75 | -274.42 | -1172.17 |
| TCGA-44-6777-01 | 1986.65 | 2264.99 | 4251.64 |
| TCGA-44-6778-01 | 643.57 | 2425.86 | 3069.43 |
| TCGA-44-6779-01 | 612.95 | 1886.98 | 2499.92 |
| TCGA-44-7659-01 | -593.67 | 614.81 | 21.15 |
| TCGA-44-7660-01 | -805.51 | 255.7 | -549.8 |
| TCGA-44-7661-01 | 858.57 | 1668.3 | 2526.87 |
| TCGA-44-7662-01 | 1127.28 | 1013.44 | 2140.72 |
| TCGA-44-7667-01 | -642.18 | -314.04 | -956.22 |
| TCGA-44-7669-01 | -163.71 | 737.61 | 573.9 |
| TCGA-44-7670-01 | -1226.8 | -288.38 | -1515.17 |
| TCGA-44-7671-01 | 39.83 | -185.72 | -145.89 |
| TCGA-44-7672-01 | 1021.73 | 2095.52 | 3117.25 |
| TCGA-44-8117-01 | 145.48 | 286.52 | 432 |
| TCGA-44-8119-01 | 941.34 | 726.62 | 1667.96 |
| TCGA-44-8120-01 | 371.86 | 557.79 | 929.65 |
| TCGA-44-A479-01 | 945.27 | 2044.8 | 2990.07 |
| TCGA-44-A47A-01 | -232.64 | 1396.49 | 1163.85 |
| TCGA-44-A47B-01 | -220.14 | 800.34 | 580.2 |
| TCGA-44-A47G-01 | 848.83 | 2129.05 | 2977.88 |
| TCGA-44-A4SS-01 | 500.03 | 1623.66 | 2123.69 |
| TCGA-44-A4SU-01 | -214.02 | 560.74 | 346.72 |
| TCGA-49-4486-01 | -1555.62 | -260.68 | -1816.3 |
| TCGA-49-4487-01 | 518.91 | 1750.25 | 2269.17 |
| TCGA-49-4488-01 | -538.81 | 952.61 | 413.79 |
| TCGA-49-4490-01 | 69.22 | 422.16 | 491.37 |
| TCGA-49-4494-01 | -441.17 | 896.17 | 455 |
| TCGA-49-4501-01 | 373.71 | 1166.92 | 1540.63 |
| TCGA-49-4505-01 | 409.47 | 1593.88 | 2003.35 |
| TCGA-49-4506-01 | -377.23 | 1325.99 | 948.76 |
| TCGA-49-4507-01 | -390.59 | 1596.48 | 1205.89 |
| TCGA-49-4510-01 | -383.28 | 134.65 | -248.62 |
| TCGA-49-4512-01 | 741.51 | 946.42 | 1687.93 |
| TCGA-49-4514-01 | -809.02 | 736.9 | -72.12 |
| TCGA-49-6742-01 | -1066.96 | -310.81 | -1377.77 |
| TCGA-49-6743-01 | 360.33 | 706.77 | 1067.1 |
| TCGA-49-6744-01 | 1055.95 | 1972.73 | 3028.68 |
| TCGA-49-6745-01 | 602.25 | 1535.71 | 2137.96 |
| TCGA-49-6761-01 | -197.34 | 1072.12 | 874.78 |
| TCGA-49-6767-01 | -478.74 | 1054.45 | 575.71 |
| TCGA-49-AAQV-01 | -703.28 | 893.75 | 190.47 |
| TCGA-49-AAR0-01 | -179.27 | 1253.03 | 1073.76 |
| TCGA-49-AAR2-01 | -802.72 | 814.21 | 11.49 |
| TCGA-49-AAR3-01 | 602.12 | 2039.42 | 2641.54 |
| TCGA-49-AAR4-01 | -102.76 | 1933.89 | 1831.13 |
| TCGA-49-AAR9-01 | -961.13 | -428.43 | -1389.55 |
| TCGA-49-AARE-01 | -239.25 | 487.32 | 248.08 |
| TCGA-49-AARN-01 | -334.94 | 559.96 | 225.02 |
| TCGA-49-AARO-01 | 574.25 | 1748.28 | 2322.53 |
| TCGA-49-AARQ-01 | -1111.21 | 617 | -494.21 |
| TCGA-49-AARR-01 | 437.88 | 1279.55 | 1717.43 |
| TCGA-4B-A93V-01 | -899.27 | 386.93 | -512.34 |
| TCGA-50-5044-01 | 148.32 | 1010.87 | 1159.18 |
| TCGA-50-5045-01 | 1430.36 | 2418.37 | 3848.73 |
| TCGA-50-5049-01 | 1639.69 | 2846.07 | 4485.76 |
| TCGA-50-5051-01 | -757.94 | -204.87 | -962.81 |
| TCGA-50-5055-01 | 1252.77 | 2735.92 | 3988.69 |
| TCGA-50-5066-01 | 913.2 | 2207.84 | 3121.04 |
| TCGA-50-5066-02 | -90.17 | 423.83 | 333.66 |
| TCGA-50-5068-01 | 515.4 | 2162.42 | 2677.82 |
| TCGA-50-5072-01 | -172.59 | 86.92 | -85.67 |
| TCGA-50-5930-01 | 484.25 | 1171.53 | 1655.78 |
| TCGA-50-5931-01 | -593.62 | -703.47 | -1297.09 |
| TCGA-50-5932-01 | -718.49 | -21.09 | -739.58 |
| TCGA-50-5933-01 | 1425.35 | 1453.04 | 2878.39 |
| TCGA-50-5935-01 | 35 | 938.7 | 973.7 |
| TCGA-50-5936-01 | 147.12 | 577.59 | 724.71 |
| TCGA-50-5939-01 | 457.22 | 1354.23 | 1811.45 |
| TCGA-50-5941-01 | 569.04 | 2174.4 | 2743.45 |
| TCGA-50-5942-01 | -30.52 | 501.69 | 471.17 |
| TCGA-50-5944-01 | 650.65 | 562.64 | 1213.29 |
| TCGA-50-5946-01 | -1100.38 | -559.22 | -1659.61 |
| TCGA-50-5946-02 | 960.71 | 324.6 | 1285.31 |
| TCGA-50-6590-01 | 337.99 | 1747.56 | 2085.55 |
| TCGA-50-6591-01 | -917.66 | -1355.85 | -2273.51 |
| TCGA-50-6592-01 | 298.71 | 1411.86 | 1710.57 |
| TCGA-50-6593-01 | 767.06 | 1117.37 | 1884.43 |
| TCGA-50-6594-01 | -318.95 | 378.12 | 59.16 |
| TCGA-50-6595-01 | 1052.86 | 1101.63 | 2154.49 |
| TCGA-50-6597-01 | -461.69 | 1239.12 | 777.44 |
| TCGA-50-6673-01 | 265.73 | 552.94 | 818.66 |
| TCGA-50-7109-01 | 85.6 | 683.67 | 769.27 |
| TCGA-50-8457-01 | 665.15 | 1706.71 | 2371.86 |
| TCGA-50-8459-01 | 1794.24 | 1991.35 | 3785.59 |
| TCGA-50-8460-01 | 89.89 | 1476.01 | 1565.9 |
| TCGA-53-7624-01 | -962.99 | 134.43 | -828.56 |
| TCGA-53-7626-01 | 654.18 | 2014.96 | 2669.14 |
| TCGA-53-7813-01 | -958.2 | 321.87 | -636.33 |
| TCGA-53-A4EZ-01 | -1114.01 | -158.97 | -1272.99 |
| TCGA-55-1592-01 | -83.44 | 678.65 | 595.2 |
| TCGA-55-1594-01 | -560.24 | 388.8 | -171.44 |
| TCGA-55-1595-01 | 492.12 | 657.33 | 1149.45 |
| TCGA-55-1596-01 | -776.21 | 115.69 | -660.52 |
| TCGA-55-5899-01 | 201.5 | 546.84 | 748.34 |
| TCGA-55-6543-01 | 1.75 | 1030.03 | 1031.78 |
| TCGA-55-6642-01 | 1331.89 | 1015.45 | 2347.34 |
| TCGA-55-6712-01 | 344.08 | 1724.57 | 2068.65 |
| TCGA-55-6968-01 | -141.36 | 1004.19 | 862.82 |
| TCGA-55-6969-01 | 765.58 | 1553.53 | 2319.11 |
| TCGA-55-6970-01 | -108.48 | 1165.87 | 1057.39 |
| TCGA-55-6971-01 | 590.76 | 2188.12 | 2778.88 |
| TCGA-55-6972-01 | -1958.42 | -890.33 | -2848.75 |
| TCGA-55-6975-01 | 856.13 | 320.2 | 1176.33 |
| TCGA-55-6978-01 | 1379.97 | 2104.05 | 3484.02 |
| TCGA-55-6979-01 | 971.94 | 2374.28 | 3346.23 |
| TCGA-55-6980-01 | 731.14 | 1570.8 | 2301.94 |
| TCGA-55-6981-01 | -197.04 | 377.39 | 180.35 |
| TCGA-55-6982-01 | 904.48 | 1098.55 | 2003.03 |
| TCGA-55-6983-01 | 385.8 | 1311.47 | 1697.27 |
| TCGA-55-6984-01 | -977.46 | 317.67 | -659.8 |
| TCGA-55-6985-01 | 507.82 | 1333.03 | 1840.85 |
| TCGA-55-6986-01 | -389.05 | 795.08 | 406.03 |
| TCGA-55-6987-01 | 512.27 | 2592.94 | 3105.21 |
| TCGA-55-7227-01 | 722.58 | 1529.54 | 2252.12 |
| TCGA-55-7281-01 | 625.98 | 1660.24 | 2286.22 |
| TCGA-55-7283-01 | -340.51 | 769.03 | 428.52 |
| TCGA-55-7284-01 | 203.86 | 1150.73 | 1354.59 |
| TCGA-55-7570-01 | -830.19 | -760.68 | -1590.87 |
| TCGA-55-7573-01 | 176.48 | 1275.68 | 1452.16 |
| TCGA-55-7574-01 | 1026.57 | 1929.01 | 2955.58 |
| TCGA-55-7576-01 | 154.81 | 812.72 | 967.54 |
| TCGA-55-7724-01 | 673.85 | 1534.79 | 2208.64 |
| TCGA-55-7725-01 | -280.89 | 1556.43 | 1275.54 |
| TCGA-55-7726-01 | 969.53 | 645.05 | 1614.58 |
| TCGA-55-7727-01 | -396.88 | 1009.84 | 612.96 |
| TCGA-55-7728-01 | -198.83 | 2426.3 | 2227.46 |
| TCGA-55-7815-01 | 867.86 | 1254.93 | 2122.79 |
| TCGA-55-7816-01 | 1655.48 | 1701.33 | 3356.81 |
| TCGA-55-7903-01 | -645.51 | 827.74 | 182.23 |
| TCGA-55-7907-01 | 236.51 | 1114.45 | 1350.96 |
| TCGA-55-7910-01 | -465.83 | -32.1 | -497.93 |
| TCGA-55-7911-01 | -270.08 | 1628.37 | 1358.29 |
| TCGA-55-7913-01 | -1508.49 | -333.2 | -1841.7 |
| TCGA-55-7914-01 | -133.73 | 755.31 | 621.57 |
| TCGA-55-7994-01 | -374.59 | 1675.55 | 1300.97 |
| TCGA-55-7995-01 | -69.72 | 1934.22 | 1864.5 |
| TCGA-55-8085-01 | -253.73 | 1308.46 | 1054.73 |
| TCGA-55-8087-01 | -375.95 | 283.83 | -92.11 |
| TCGA-55-8089-01 | 572.96 | 2495.84 | 3068.8 |
| TCGA-55-8090-01 | -43.21 | 771.29 | 728.08 |
| TCGA-55-8091-01 | 973.34 | 1378.71 | 2352.05 |
| TCGA-55-8092-01 | 152.69 | 1472.48 | 1625.17 |
| TCGA-55-8094-01 | -1959.31 | -1004.32 | -2963.63 |
| TCGA-55-8096-01 | 944.87 | 1211.05 | 2155.92 |
| TCGA-55-8097-01 | -64.86 | 660.76 | 595.9 |
| TCGA-55-8203-01 | -70.47 | 935.04 | 864.57 |
| TCGA-55-8204-01 | -140.39 | 834.84 | 694.45 |
| TCGA-55-8205-01 | 647.93 | 2213.07 | 2861.01 |
| TCGA-55-8206-01 | 313.09 | 1882.97 | 2196.07 |
| TCGA-55-8207-01 | 931.94 | 1218.22 | 2150.16 |
| TCGA-55-8208-01 | 1229.39 | 2493.47 | 3722.86 |
| TCGA-55-8299-01 | 1285.57 | 2096.41 | 3381.98 |
| TCGA-55-8301-01 | 632.28 | 2126.21 | 2758.49 |
| TCGA-55-8302-01 | -497.37 | 760.01 | 262.65 |
| TCGA-55-8505-01 | 147.09 | -273.52 | -126.43 |
| TCGA-55-8506-01 | -78.06 | 812.03 | 733.97 |
| TCGA-55-8507-01 | -301.67 | 282.23 | -19.43 |
| TCGA-55-8508-01 | -88.69 | 728.07 | 639.38 |
| TCGA-55-8510-01 | 608.95 | 1830.03 | 2438.98 |
| TCGA-55-8511-01 | 549.38 | 1367.24 | 1916.62 |
| TCGA-55-8512-01 | -591.69 | 215.31 | -376.38 |
| TCGA-55-8513-01 | 503.88 | 1754.91 | 2258.79 |
| TCGA-55-8514-01 | -466.73 | 640.12 | 173.39 |
| TCGA-55-8614-01 | 23.52 | 236.29 | 259.81 |
| TCGA-55-8615-01 | -727.37 | -272.08 | -999.45 |
| TCGA-55-8616-01 | -419.49 | 278.38 | -141.1 |
| TCGA-55-8619-01 | 542.51 | 2150.43 | 2692.94 |
| TCGA-55-8620-01 | -1030.18 | 433.59 | -596.58 |
| TCGA-55-8621-01 | 979.96 | 2115.34 | 3095.3 |
| TCGA-55-A48X-01 | 86.77 | 1495.84 | 1582.61 |
| TCGA-55-A48Y-01 | 518.75 | 421.03 | 939.78 |
| TCGA-55-A48Z-01 | 562.93 | 791.06 | 1353.99 |
| TCGA-55-A490-01 | 234.04 | 683.14 | 917.18 |
| TCGA-55-A491-01 | 122.44 | 1166.68 | 1289.12 |
| TCGA-55-A492-01 | -782.79 | 173.43 | -609.36 |
| TCGA-55-A493-01 | 84.21 | 1603.38 | 1687.58 |
| TCGA-55-A494-01 | -1324.01 | -1014.81 | -2338.82 |
| TCGA-55-A4DF-01 | -429.06 | 1137.51 | 708.45 |
| TCGA-55-A4DG-01 | -512.14 | 904.58 | 392.44 |
| TCGA-55-A57B-01 | 248.14 | 918.24 | 1166.37 |
| TCGA-62-8394-01 | -517.26 | 375.35 | -141.91 |
| TCGA-62-8395-01 | 286.13 | 175.31 | 461.43 |
| TCGA-62-8397-01 | -240.22 | 895.35 | 655.14 |
| TCGA-62-8398-01 | -240.51 | 695.22 | 454.71 |
| TCGA-62-8399-01 | -73.15 | 107.36 | 34.22 |
| TCGA-62-8402-01 | -807.52 | 1313.22 | 505.7 |
| TCGA-62-A46O-01 | -1647.23 | -1098.74 | -2745.97 |
| TCGA-62-A46P-01 | -619.26 | -47.42 | -666.68 |
| TCGA-62-A46R-01 | -93.64 | 1204.85 | 1111.21 |
| TCGA-62-A46S-01 | -464.19 | 641.82 | 177.64 |
| TCGA-62-A46U-01 | -5.09 | 2534.86 | 2529.77 |
| TCGA-62-A46V-01 | 103.57 | 185.23 | 288.8 |
| TCGA-62-A46Y-01 | -332.99 | 1483.53 | 1150.54 |
| TCGA-62-A470-01 | -703.08 | 133.7 | -569.38 |
| TCGA-62-A471-01 | -1446.62 | -186.32 | -1632.94 |
| TCGA-62-A472-01 | -680.93 | 859.27 | 178.34 |
| TCGA-64-1676-01 | 534.96 | 1720.12 | 2255.08 |
| TCGA-64-1677-01 | -888.39 | 802.42 | -85.97 |
| TCGA-64-1678-01 | -1092.84 | -609.99 | -1702.82 |
| TCGA-64-1679-01 | 1064.08 | 877.76 | 1941.84 |
| TCGA-64-1680-01 | -915.82 | 226.42 | -689.4 |
| TCGA-64-1681-01 | 99.19 | 948.01 | 1047.2 |
| TCGA-64-5774-01 | -692.62 | -529.23 | -1221.85 |
| TCGA-64-5775-01 | -130.35 | 407.23 | 276.87 |
| TCGA-64-5778-01 | -537.66 | 1864.53 | 1326.87 |
| TCGA-64-5779-01 | 360.85 | 725.43 | 1086.28 |
| TCGA-64-5781-01 | 6.68 | 1368.25 | 1374.93 |
| TCGA-64-5815-01 | 1457.51 | 1464.83 | 2922.34 |
| TCGA-67-3770-01 | -189.5 | 1238.99 | 1049.5 |
| TCGA-67-3771-01 | 271.68 | 1066.71 | 1338.39 |
| TCGA-67-3772-01 | 771.41 | 1171.82 | 1943.23 |
| TCGA-67-3773-01 | 422.7 | 1548.55 | 1971.25 |
| TCGA-67-3774-01 | -6.81 | 1124.26 | 1117.45 |
| TCGA-67-4679-01 | 349.41 | 1172.87 | 1522.28 |
| TCGA-67-6215-01 | -825.59 | 693.22 | -132.37 |
| TCGA-67-6216-01 | -337.91 | 1315.55 | 977.64 |
| TCGA-67-6217-01 | 50.99 | 1251.38 | 1302.36 |
| TCGA-69-7760-01 | -678.68 | -457.5 | -1136.18 |
| TCGA-69-7761-01 | 373.6 | 1822.97 | 2196.56 |
| TCGA-69-7763-01 | 645.59 | 558.97 | 1204.56 |
| TCGA-69-7764-01 | -412.83 | 330.91 | -81.91 |
| TCGA-69-7765-01 | 1086.21 | 1079.11 | 2165.32 |
| TCGA-69-7973-01 | -201.31 | -142.75 | -344.06 |
| TCGA-69-7974-01 | 437.33 | 1577.74 | 2015.07 |
| TCGA-69-7978-01 | 939.94 | 2205.25 | 3145.2 |
| TCGA-69-7979-01 | -199.09 | -181.52 | -380.61 |
| TCGA-69-7980-01 | 151.19 | 1096.91 | 1248.11 |
| TCGA-69-8253-01 | -689.81 | 379.46 | -310.36 |
| TCGA-69-8254-01 | -565.74 | 1026.02 | 460.27 |
| TCGA-69-8255-01 | -987.02 | 1350.23 | 363.21 |
| TCGA-69-8453-01 | 421.26 | 2188.01 | 2609.27 |
| TCGA-69-A59K-01 | -349.33 | 761.1 | 411.77 |
| TCGA-71-6725-01 | -953.41 | 141.19 | -812.22 |
| TCGA-71-8520-01 | 432.86 | 309.91 | 742.77 |
| TCGA-73-4658-01 | 1336.99 | 1917.98 | 3254.97 |
| TCGA-73-4659-01 | 435.09 | 698.1 | 1133.19 |
| TCGA-73-4662-01 | 64.72 | 1359.37 | 1424.09 |
| TCGA-73-4666-01 | -87.83 | 1814.71 | 1726.88 |
| TCGA-73-4668-01 | -31.24 | 191.44 | 160.2 |
| TCGA-73-4670-01 | -34.45 | 182.59 | 148.14 |
| TCGA-73-4675-01 | 174.63 | 381.76 | 556.39 |
| TCGA-73-4676-01 | -284.68 | 537.66 | 252.98 |
| TCGA-73-4677-01 | -462.7 | 605.39 | 142.7 |
| TCGA-73-7498-01 | 186.29 | 685.92 | 872.21 |
| TCGA-73-7499-01 | -102.58 | 1678.33 | 1575.74 |
| TCGA-73-A9RS-01 | -1064.24 | -104.38 | -1168.62 |
| TCGA-75-5122-01 | 688.81 | 3286.67 | 3975.48 |
| TCGA-75-5125-01 | 255.7 | 1493.59 | 1749.29 |
| TCGA-75-5126-01 | 615.01 | 2175.77 | 2790.78 |
| TCGA-75-5146-01 | -228.78 | 1050.63 | 821.85 |
| TCGA-75-5147-01 | 56.27 | 1108.3 | 1164.56 |
| TCGA-75-6203-01 | 744.6 | 2188.81 | 2933.42 |
| TCGA-75-6205-01 | 1321.03 | 2536.32 | 3857.35 |
| TCGA-75-6206-01 | 150.48 | 649.07 | 799.55 |
| TCGA-75-6207-01 | -644.98 | 241.76 | -403.22 |
| TCGA-75-6211-01 | -179 | -73.49 | -252.49 |
| TCGA-75-6212-01 | -1.06 | 1540.49 | 1539.42 |
| TCGA-75-6214-01 | -1118.06 | -91.39 | -1209.45 |
| TCGA-75-7025-01 | 356.8 | 1211.78 | 1568.58 |
| TCGA-75-7027-01 | 299.69 | -73.13 | 226.57 |
| TCGA-75-7030-01 | 904.53 | 1186.74 | 2091.27 |
| TCGA-75-7031-01 | -586.62 | 883.23 | 296.62 |
| TCGA-78-7143-01 | -856.84 | 728.44 | -128.39 |
| TCGA-78-7145-01 | -19.3 | 431.58 | 412.28 |
| TCGA-78-7146-01 | -724.67 | 239.26 | -485.41 |
| TCGA-78-7147-01 | -1030.99 | 401.51 | -629.49 |
| TCGA-78-7148-01 | -572.54 | 420.29 | -152.26 |
| TCGA-78-7149-01 | -255.7 | -147.69 | -403.39 |
| TCGA-78-7150-01 | -67.35 | -407.19 | -474.54 |
| TCGA-78-7152-01 | -289.63 | 980.35 | 690.72 |
| TCGA-78-7153-01 | -774.99 | 31.06 | -743.93 |
| TCGA-78-7154-01 | -567.99 | -166.77 | -734.76 |
| TCGA-78-7155-01 | -1206.67 | -639.02 | -1845.69 |
| TCGA-78-7156-01 | -841.28 | -228.36 | -1069.64 |
| TCGA-78-7158-01 | -790.49 | 69.42 | -721.07 |
| TCGA-78-7159-01 | -92.51 | 104.02 | 11.51 |
| TCGA-78-7160-01 | 555.45 | 1564.1 | 2119.55 |
| TCGA-78-7161-01 | -365 | -559.96 | -924.96 |
| TCGA-78-7162-01 | 235.08 | 1047.91 | 1283 |
| TCGA-78-7163-01 | -1926.5 | -204.63 | -2131.13 |
| TCGA-78-7166-01 | -1109.82 | 187.51 | -922.31 |
| TCGA-78-7167-01 | -754.65 | -380.76 | -1135.42 |
| TCGA-78-7220-01 | -881.72 | -429.82 | -1311.53 |
| TCGA-78-7535-01 | -226 | 1204.4 | 978.4 |
| TCGA-78-7536-01 | -511.46 | 653.59 | 142.13 |
| TCGA-78-7537-01 | -17.33 | 243.82 | 226.49 |
| TCGA-78-7539-01 | -313.48 | 1514.13 | 1200.65 |
| TCGA-78-7540-01 | -910.95 | 401.41 | -509.54 |
| TCGA-78-7542-01 | -1411.15 | 335.17 | -1075.98 |
| TCGA-78-7633-01 | -653.54 | -337.96 | -991.5 |
| TCGA-78-8640-01 | -842.08 | 495.31 | -346.77 |
| TCGA-78-8648-01 | 1939.43 | 2471.95 | 4411.38 |
| TCGA-78-8655-01 | 12.6 | 1264.9 | 1277.49 |
| TCGA-78-8660-01 | -104.42 | 1661.9 | 1557.48 |
| TCGA-78-8662-01 | -1050.79 | -446.71 | -1497.51 |
| TCGA-80-5607-01 | -310.11 | 1154.88 | 844.77 |
| TCGA-80-5608-01 | -833.11 | 284.49 | -548.62 |
| TCGA-80-5611-01 | -350.78 | 1564.17 | 1213.39 |
| TCGA-83-5908-01 | 212.01 | 1830.63 | 2042.64 |
| TCGA-86-6562-01 | 700.02 | 577.68 | 1277.7 |
| TCGA-86-6851-01 | 304.41 | 2246.5 | 2550.9 |
| TCGA-86-7701-01 | 561.46 | 1485.1 | 2046.57 |
| TCGA-86-7711-01 | 540.5 | 1246.27 | 1786.76 |
| TCGA-86-7713-01 | -1116.3 | -568.26 | -1684.57 |
| TCGA-86-7714-01 | -56.39 | 860.95 | 804.56 |
| TCGA-86-7953-01 | 32.37 | 1306.8 | 1339.18 |
| TCGA-86-7954-01 | 349.27 | 1723.88 | 2073.15 |
| TCGA-86-7955-01 | -1774.01 | -934.73 | -2708.74 |
| TCGA-86-8054-01 | -676.66 | -621.28 | -1297.94 |
| TCGA-86-8055-01 | 1229.16 | 898.35 | 2127.52 |
| TCGA-86-8056-01 | -96.79 | 817.3 | 720.51 |
| TCGA-86-8073-01 | 333.15 | 529.26 | 862.41 |
| TCGA-86-8074-01 | 639.36 | 867.19 | 1506.56 |
| TCGA-86-8075-01 | 1090.75 | 912.49 | 2003.24 |
| TCGA-86-8076-01 | 66.86 | 1860 | 1926.86 |
| TCGA-86-8278-01 | 381.27 | 731.87 | 1113.14 |
| TCGA-86-8279-01 | 241.37 | 74.03 | 315.4 |
| TCGA-86-8280-01 | 611.91 | 1600.49 | 2212.41 |
| TCGA-86-8281-01 | -402.9 | 159.13 | -243.78 |
| TCGA-86-8358-01 | -617.74 | -263.38 | -881.12 |
| TCGA-86-8359-01 | -369.79 | 1166.42 | 796.63 |
| TCGA-86-8585-01 | -118.7 | 1648.05 | 1529.35 |
| TCGA-86-8668-01 | 716.37 | 1089.62 | 1806 |
| TCGA-86-8669-01 | -388.39 | 744.53 | 356.14 |
| TCGA-86-8671-01 | 1394.5 | 2905.3 | 4299.81 |
| TCGA-86-8672-01 | 457.64 | 1380.47 | 1838.11 |
| TCGA-86-8673-01 | -138.92 | 521.22 | 382.3 |
| TCGA-86-8674-01 | -1110.3 | -506.36 | -1616.66 |
| TCGA-86-A456-01 | 128.48 | 1206.48 | 1334.96 |
| TCGA-86-A4D0-01 | -1233.81 | -279.35 | -1513.16 |
| TCGA-86-A4JF-01 | -412.06 | 1148.44 | 736.38 |
| TCGA-86-A4P7-01 | 468.31 | 1483.82 | 1952.13 |
| TCGA-86-A4P8-01 | 914.62 | 2466.21 | 3380.83 |
| TCGA-91-6828-01 | 522.58 | 1559.91 | 2082.49 |
| TCGA-91-6829-01 | 1133.05 | 334.11 | 1467.16 |
| TCGA-91-6830-01 | 662.42 | 1121.7 | 1784.12 |
| TCGA-91-6831-01 | 623.64 | 675.8 | 1299.44 |
| TCGA-91-6835-01 | 889.87 | 2383.01 | 3272.87 |
| TCGA-91-6836-01 | -1044.37 | -40.84 | -1085.21 |
| TCGA-91-6840-01 | -353.97 | 644.69 | 290.72 |
| TCGA-91-6847-01 | -1800.3 | -975.56 | -2775.86 |
| TCGA-91-6848-01 | 1015.64 | 1540.41 | 2556.05 |
| TCGA-91-6849-01 | -66.35 | 1208.09 | 1141.74 |
| TCGA-91-7771-01 | 646.44 | 1633.44 | 2279.88 |
| TCGA-91-8496-01 | -7.83 | 1772.54 | 1764.71 |
| TCGA-91-8497-01 | 460.48 | 1619.55 | 2080.03 |
| TCGA-91-8499-01 | -485.42 | 399.84 | -85.58 |
| TCGA-91-A4BC-01 | 962.48 | 2279.43 | 3241.92 |
| TCGA-91-A4BD-01 | -707.39 | 1485.39 | 778 |
| TCGA-93-7347-01 | 874 | 2212.91 | 3086.91 |
| TCGA-93-7348-01 | 423.65 | 737.06 | 1160.72 |
| TCGA-93-8067-01 | -717.86 | 45.21 | -672.65 |
| TCGA-93-A4JN-01 | 173.2 | 727.37 | 900.58 |
| TCGA-93-A4JO-01 | 344.7 | 1898.74 | 2243.44 |
| TCGA-93-A4JP-01 | 370.73 | 1175.01 | 1545.74 |
| TCGA-93-A4JQ-01 | 805.11 | 1754.53 | 2559.64 |
| TCGA-95-7039-01 | -15.51 | 326.9 | 311.39 |
| TCGA-95-7043-01 | -660.62 | -624.74 | -1285.36 |
| TCGA-95-7562-01 | -149.93 | 681.77 | 531.84 |
| TCGA-95-7567-01 | 36.85 | 754.17 | 791.01 |
| TCGA-95-7944-01 | 24.14 | 1836.14 | 1860.28 |
| TCGA-95-7947-01 | -866.7 | 621.22 | -245.48 |
| TCGA-95-7948-01 | -575.76 | 138.89 | -436.87 |
| TCGA-95-8039-01 | -71.44 | 1382 | 1310.56 |
| TCGA-95-8494-01 | -581.14 | 867.64 | 286.5 |
| TCGA-95-A4VK-01 | -519.51 | 707.03 | 187.52 |
| TCGA-95-A4VN-01 | 149.1 | 1743.76 | 1892.86 |
| TCGA-95-A4VP-01 | 127.65 | 984.41 | 1112.07 |
| TCGA-97-7546-01 | 659.77 | 1221.68 | 1881.45 |
| TCGA-97-7547-01 | 47.88 | 616.86 | 664.73 |
| TCGA-97-7552-01 | 311.03 | 2267.1 | 2578.12 |
| TCGA-97-7553-01 | 807.24 | 2375.74 | 3182.98 |
| TCGA-97-7554-01 | 1054.4 | 837.9 | 1892.29 |
| TCGA-97-7937-01 | -299.04 | -38.36 | -337.39 |
| TCGA-97-7938-01 | -103.95 | 385.76 | 281.81 |
| TCGA-97-7941-01 | 5.96 | 697.48 | 703.44 |
| TCGA-97-8171-01 | -1263.52 | -404.22 | -1667.74 |
| TCGA-97-8172-01 | 798.9 | 1642.24 | 2441.15 |
| TCGA-97-8174-01 | 759.14 | 1072.77 | 1831.91 |
| TCGA-97-8175-01 | -354.97 | 1120.1 | 765.14 |
| TCGA-97-8176-01 | -468.26 | 528.15 | 59.89 |
| TCGA-97-8177-01 | 890.52 | 1682.16 | 2572.68 |
| TCGA-97-8179-01 | -203.94 | 238.11 | 34.17 |
| TCGA-97-8547-01 | 933.31 | 816 | 1749.31 |
| TCGA-97-8552-01 | -56.58 | 1425.01 | 1368.44 |
| TCGA-97-A4LX-01 | 1073.04 | 2312.56 | 3385.61 |
| TCGA-97-A4M0-01 | -353.83 | 1550.94 | 1197.11 |
| TCGA-97-A4M1-01 | -62.73 | 1148.37 | 1085.64 |
| TCGA-97-A4M2-01 | 310.71 | 2085.83 | 2396.55 |
| TCGA-97-A4M3-01 | -96.53 | -49.79 | -146.32 |
| TCGA-97-A4M5-01 | 455.52 | 1140.02 | 1595.54 |
| TCGA-97-A4M6-01 | 323.17 | 1743.83 | 2067 |
| TCGA-97-A4M7-01 | 351.63 | 1686.79 | 2038.42 |
| TCGA-99-7458-01 | 734.9 | 1603.9 | 2338.8 |
| TCGA-99-8025-01 | -12.83 | 124.59 | 111.77 |
| TCGA-99-8028-01 | 1305.02 | 2756.73 | 4061.75 |
| TCGA-99-8032-01 | 507.44 | 341.74 | 849.18 |
| TCGA-99-8033-01 | -311.45 | 1082.74 | 771.29 |
| TCGA-99-AA5R-01 | 876.07 | 2313.11 | 3189.18 |
| TCGA-J2-8192-01 | 1487.82 | 1558.13 | 3045.95 |
| TCGA-J2-8194-01 | 119.69 | 630.25 | 749.95 |
| TCGA-J2-A4AD-01 | -432.07 | -399.89 | -831.96 |
| TCGA-J2-A4AE-01 | -605.53 | 1057.3 | 451.77 |
| TCGA-J2-A4AG-01 | 334.08 | 1381.48 | 1715.55 |
| TCGA-L4-A4E5-01 | -707.04 | 26.44 | -680.6 |
| TCGA-L4-A4E6-01 | 934.25 | 2309.16 | 3243.41 |
| TCGA-L9-A443-01 | 190.21 | 473.85 | 664.06 |
| TCGA-L9-A444-01 | 598.66 | 2247.22 | 2845.88 |
| TCGA-L9-A50W-01 | -443.11 | 673.3 | 230.19 |
| TCGA-L9-A5IP-01 | -713.87 | 503.8 | -210.07 |
| TCGA-L9-A743-01 | 601.71 | 1926.79 | 2528.5 |
| TCGA-L9-A7SV-01 | -1057.04 | -241.17 | -1298.21 |
| TCGA-L9-A8F4-01 | 85.67 | 1507.06 | 1592.73 |
| TCGA-MN-A4N1-01 | -670.72 | -248.15 | -918.87 |
| TCGA-MN-A4N4-01 | 554.3 | 502.62 | 1056.92 |
| TCGA-MN-A4N5-01 | -654.03 | 911.88 | 257.85 |
| TCGA-MP-A4SV-01 | -31.72 | 1142.36 | 1110.64 |
| TCGA-MP-A4SW-01 | -55.39 | 1282.55 | 1227.16 |
| TCGA-MP-A4SY-01 | 514.21 | 460.25 | 974.46 |
| TCGA-MP-A4T4-01 | 709.1 | 1941.73 | 2650.84 |
| TCGA-MP-A4T6-01 | -1165.17 | 843.52 | -321.65 |
| TCGA-MP-A4T7-01 | -689.28 | 760.09 | 70.82 |
| TCGA-MP-A4T8-01 | -62.11 | -250.01 | -312.11 |
| TCGA-MP-A4T9-01 | 291.21 | 1080.11 | 1371.32 |
| TCGA-MP-A4TA-01 | -430.12 | 584.79 | 154.67 |
| TCGA-MP-A4TC-01 | 876.36 | 1139.87 | 2016.23 |
| TCGA-MP-A4TD-01 | 746.26 | 764.72 | 1510.98 |
| TCGA-MP-A4TE-01 | -1233.68 | -792.11 | -2025.79 |
| TCGA-MP-A4TF-01 | -1257.44 | 188.26 | -1069.18 |
| TCGA-MP-A4TH-01 | 106.63 | 1736.31 | 1842.94 |
| TCGA-MP-A4TI-01 | 1180.44 | 2588.41 | 3768.84 |
| TCGA-MP-A4TJ-01 | 912.74 | 2446.84 | 3359.58 |
| TCGA-MP-A4TK-01 | 1259 | 1478.45 | 2737.45 |
| TCGA-MP-A5C7-01 | -1179.69 | -553.01 | -1732.69 |
| TCGA-NJ-A4YF-01 | -802.78 | -168.6 | -971.38 |
| TCGA-NJ-A4YG-01 | 127.1 | 1076.06 | 1203.16 |
| TCGA-NJ-A4YI-01 | 80.5 | 936.15 | 1016.64 |
| TCGA-NJ-A4YP-01 | 746.63 | 594.12 | 1340.75 |
| TCGA-NJ-A4YQ-01 | -63.7 | 2022.22 | 1958.52 |
| TCGA-NJ-A55A-01 | 197.29 | 1419.34 | 1616.63 |
| TCGA-NJ-A55O-01 | -339.44 | 922.95 | 583.51 |
| TCGA-NJ-A55R-01 | -437.48 | -189.12 | -626.6 |
| TCGA-NJ-A7XG-01 | -1710.14 | -282.7 | -1992.84 |
| TCGA-O1-A52J-01 | -297.9 | 1265.87 | 967.97 |
| TCGA-S2-AA1A-01 | 735.65 | 1916.74 | 2652.39 |
