# Supplementary material for: TRIM28 is a distinct prognostic biomarker that worsens the tumor immune microenvironment in lung adenocarcinoma
Source: Aging (Albany NY). 2020 Oct 22;12(20):20308–31. doi: 10.18632/aging.103804 (PMC7655206; doi:10.18632/aging.103804)
Supplement: Supplementary Table 3 [file aging-12-103804-s004..docx]

Supplementary Table 3. The hub genes were screened by applying the cytoHubba plugin.

| node_name | MCC | DMNC | MNC | Degree | EPC | BottleNeck | EcCentricity | Closeness | Radiality | Betweenness | Stress | ClusteringCoefficient |
| --- | --- | --- | --- | --- | --- | --- | --- | --- | --- | --- | --- | --- |
| BDH1 | 22.00 | 0.29 | 7.00 | 11.00 | 48.02 | 8.00 | 0.25 | 38.83 | 5.53 | 590.42 | 1708.00 | 0.16 |
| ARFIP2 | 6.00 | 0.31 | 2.00 | 6.00 | 40.23 | 5.00 | 0.25 | 37.25 | 5.53 | 482.36 | 1218.00 | 0.07 |
| RAB25 | 131065.00 | 0.47 | 17.00 | 20.00 | 53.13 | 13.00 | 0.20 | 45.78 | 5.80 | 1080.81 | 3230.00 | 0.31 |
| TRIM28 | 31.00 | 0.28 | 10.00 | 11.00 | 49.34 | 7.00 | 0.20 | 40.28 | 5.62 | 571.76 | 1782.00 | 0.25 |
| EIF2B4 | 7.00 | 0.31 | 3.00 | 6.00 | 42.24 | 7.00 | 0.20 | 36.62 | 5.47 | 283.92 | 950.00 | 0.13 |
| PIM3 | 5.00 | 0.31 | 2.00 | 5.00 | 37.07 | 7.00 | 0.20 | 34.78 | 5.33 | 245.76 | 734.00 | 0.10 |
| BAG6 | 26.00 | 0.24 | 10.00 | 10.00 | 48.67 | 6.00 | 0.20 | 39.12 | 5.55 | 487.52 | 1442.00 | 0.27 |
| DDX1 | 16.00 | 0.29 | 6.00 | 10.00 | 45.35 | 6.00 | 0.20 | 37.12 | 5.38 | 599.04 | 1882.00 | 0.16 |
| CLUH | 11.00 | 0.26 | 5.00 | 8.00 | 47.53 | 5.00 | 0.20 | 37.57 | 5.46 | 435.32 | 1362.00 | 0.18 |
| LETM1 | 5.00 | 0.00 | 1.00 | 5.00 | 27.21 | 5.00 | 0.20 | 30.43 | 4.85 | 435.72 | 1090.00 | 0.00 |
| ARHGEF16 | 132488.00 | 0.73 | 13.00 | 15.00 | 52.49 | 4.00 | 0.20 | 41.53 | 5.59 | 488.16 | 1378.00 | 0.54 |
| COPG1 | 7.00 | 0.31 | 2.00 | 7.00 | 45.46 | 4.00 | 0.20 | 38.87 | 5.62 | 447.22 | 1338.00 | 0.05 |
| COASY | 19.00 | 0.38 | 6.00 | 7.00 | 43.98 | 4.00 | 0.20 | 34.20 | 5.14 | 302.92 | 854.00 | 0.38 |
| GSTP1 | 8.00 | 0.46 | 3.00 | 5.00 | 41.79 | 4.00 | 0.20 | 35.62 | 5.41 | 320.45 | 924.00 | 0.30 |
| ATG4D | 2.00 | 0.00 | 1.00 | 2.00 | 19.61 | 4.00 | 0.20 | 27.28 | 4.68 | 66.57 | 202.00 | 0.00 |
| TKT | 20.00 | 0.45 | 5.00 | 7.00 | 45.58 | 3.00 | 0.20 | 35.37 | 5.34 | 252.52 | 772.00 | 0.33 |
| NDUFS2 | 14.00 | 0.47 | 4.00 | 6.00 | 42.84 | 3.00 | 0.20 | 32.15 | 4.98 | 110.00 | 294.00 | 0.33 |
| PYCR2 | 5.00 | 0.31 | 2.00 | 5.00 | 37.57 | 3.00 | 0.20 | 32.73 | 5.13 | 140.64 | 368.00 | 0.10 |
| DHRS13 | 4.00 | 0.00 | 1.00 | 4.00 | 31.84 | 3.00 | 0.20 | 30.90 | 5.01 | 92.70 | 252.00 | 0.00 |
| PACSIN3 | 4.00 | 0.00 | 1.00 | 4.00 | 29.54 | 3.00 | 0.20 | 32.35 | 5.13 | 286.06 | 668.00 | 0.00 |
| SHMT1 | 28.00 | 0.24 | 10.00 | 10.00 | 48.39 | 2.00 | 0.20 | 36.60 | 5.32 | 220.15 | 890.00 | 0.27 |
| MRPS9 | 19.00 | 0.45 | 5.00 | 8.00 | 45.94 | 2.00 | 0.20 | 35.40 | 5.26 | 277.21 | 856.00 | 0.29 |
| MPHOSPH10 | 15.00 | 0.47 | 4.00 | 7.00 | 42.93 | 2.00 | 0.20 | 34.87 | 5.28 | 235.52 | 720.00 | 0.24 |
| GNL3 | 15.00 | 0.39 | 5.00 | 6.00 | 42.86 | 2.00 | 0.20 | 36.62 | 5.47 | 309.35 | 994.00 | 0.40 |
| ZBTB12 | 9.00 | 0.26 | 5.00 | 6.00 | 41.09 | 2.00 | 0.20 | 33.77 | 5.19 | 212.00 | 560.00 | 0.27 |
| NFS1 | 7.00 | 0.31 | 3.00 | 6.00 | 39.60 | 2.00 | 0.20 | 31.07 | 4.86 | 124.98 | 344.00 | 0.13 |
| TRAF4 | 5.00 | 0.31 | 2.00 | 5.00 | 38.57 | 2.00 | 0.20 | 33.70 | 5.24 | 128.64 | 392.00 | 0.10 |
| TRIM27 | 5.00 | 0.31 | 3.00 | 4.00 | 33.61 | 2.00 | 0.20 | 31.98 | 5.11 | 76.47 | 170.00 | 0.33 |
| CCAR1 | 7.00 | 0.46 | 3.00 | 4.00 | 31.88 | 2.00 | 0.20 | 28.90 | 4.76 | 39.43 | 114.00 | 0.50 |
| ABCB6 | 4.00 | 0.00 | 1.00 | 4.00 | 31.15 | 2.00 | 0.20 | 29.55 | 4.84 | 181.66 | 410.00 | 0.00 |
| DHTKD1 | 16.00 | 0.38 | 6.00 | 6.00 | 44.56 | 1.00 | 0.20 | 32.03 | 4.96 | 38.28 | 168.00 | 0.53 |
| KIAA1804 | 9.00 | 0.38 | 4.00 | 5.00 | 42.78 | 1.00 | 0.20 | 35.12 | 5.35 | 62.37 | 226.00 | 0.40 |
| DARS2 | 10.00 | 0.38 | 4.00 | 6.00 | 42.47 | 1.00 | 0.20 | 33.32 | 5.14 | 122.62 | 374.00 | 0.27 |
| SMARCD2 | 11.00 | 0.32 | 5.00 | 6.00 | 40.82 | 1.00 | 0.20 | 31.67 | 4.96 | 76.29 | 224.00 | 0.33 |
| RBM4 | 4.00 | 0.31 | 2.00 | 4.00 | 39.81 | 1.00 | 0.20 | 34.32 | 5.33 | 148.76 | 674.00 | 0.17 |
| NOL10 | 14.00 | 0.39 | 5.00 | 5.00 | 37.53 | 1.00 | 0.20 | 31.35 | 4.99 | 28.90 | 110.00 | 0.60 |
| NELFA | 4.00 | 0.00 | 1.00 | 4.00 | 31.61 | 1.00 | 0.20 | 32.12 | 5.14 | 192.77 | 516.00 | 0.00 |
| ICA1 | 3.00 | 0.31 | 2.00 | 3.00 | 30.07 | 1.00 | 0.20 | 31.20 | 5.09 | 35.49 | 150.00 | 0.33 |
| NELFCD | 3.00 | 0.31 | 2.00 | 3.00 | 30.06 | 1.00 | 0.20 | 30.02 | 4.95 | 60.61 | 164.00 | 0.33 |
| SMPD4 | 2.00 | 0.00 | 1.00 | 2.00 | 26.54 | 1.00 | 0.20 | 30.43 | 5.01 | 31.17 | 98.00 | 0.00 |
| STARD10 | 2.00 | 0.00 | 1.00 | 2.00 | 25.66 | 1.00 | 0.20 | 28.80 | 4.85 | 23.87 | 114.00 | 0.00 |
| PRPF40B | 3.00 | 0.00 | 1.00 | 3.00 | 24.66 | 1.00 | 0.20 | 30.13 | 4.93 | 163.49 | 434.00 | 0.00 |
| FN3K | 2.00 | 0.00 | 1.00 | 2.00 | 21.37 | 1.00 | 0.20 | 27.10 | 4.65 | 11.43 | 36.00 | 0.00 |
| EPCAM | 120972.00 | 0.66 | 12.00 | 16.00 | 53.13 | 15.00 | 0.17 | 44.45 | 5.80 | 1070.79 | 3202.00 | 0.38 |
| LLGL2 | 86409.00 | 0.63 | 13.00 | 14.00 | 52.91 | 6.00 | 0.17 | 42.12 | 5.65 | 378.87 | 1340.00 | 0.54 |
| EHMT2 | 15.00 | 0.32 | 5.00 | 10.00 | 46.69 | 6.00 | 0.17 | 38.45 | 5.47 | 655.77 | 1744.00 | 0.13 |
| RBBP8NL | 132481.00 | 0.80 | 12.00 | 13.00 | 52.85 | 4.00 | 0.17 | 41.12 | 5.61 | 246.12 | 900.00 | 0.71 |
| EPN3 | 11527.00 | 0.53 | 12.00 | 13.00 | 52.43 | 4.00 | 0.17 | 39.07 | 5.40 | 351.19 | 1522.00 | 0.46 |
| HID1 | 5.00 | 0.31 | 2.00 | 5.00 | 39.13 | 4.00 | 0.17 | 32.30 | 5.04 | 153.39 | 540.00 | 0.10 |
| AP1M2 | 131050.00 | 0.66 | 13.00 | 15.00 | 52.89 | 3.00 | 0.17 | 41.20 | 5.54 | 396.57 | 1152.00 | 0.50 |
| FAM83H | 40322.00 | 0.82 | 8.00 | 10.00 | 50.81 | 3.00 | 0.17 | 37.78 | 5.41 | 334.00 | 794.00 | 0.62 |
| METTL21A | 3.00 | 0.31 | 2.00 | 3.00 | 27.67 | 3.00 | 0.17 | 29.42 | 4.85 | 59.81 | 178.00 | 0.33 |
| ESRP1 | 132486.00 | 0.65 | 14.00 | 14.00 | 52.89 | 2.00 | 0.17 | 40.70 | 5.54 | 115.69 | 656.00 | 0.64 |
| CLDN7 | 132480.00 | 0.80 | 12.00 | 12.00 | 52.61 | 2.00 | 0.17 | 39.37 | 5.48 | 21.80 | 224.00 | 0.83 |
| CAMSAP3 | 1444.00 | 0.61 | 8.00 | 10.00 | 51.52 | 2.00 | 0.17 | 36.27 | 5.24 | 152.42 | 608.00 | 0.47 |
| PATZ1 | 14.00 | 0.39 | 5.00 | 5.00 | 41.11 | 2.00 | 0.17 | 34.37 | 5.27 | 53.35 | 226.00 | 0.60 |
| ARHGAP8 | 9.00 | 0.38 | 4.00 | 5.00 | 40.24 | 2.00 | 0.17 | 32.15 | 4.98 | 174.83 | 654.00 | 0.40 |
| SPIRE2 | 3.00 | 0.31 | 2.00 | 3.00 | 33.61 | 2.00 | 0.17 | 33.53 | 5.28 | 71.47 | 178.00 | 0.33 |
| RHBDD3 | 2.00 | 0.00 | 1.00 | 2.00 | 9.80 | 2.00 | 0.17 | 22.68 | 3.88 | 168.00 | 426.00 | 0.00 |
| OVOL2 | 46080.00 | 0.78 | 10.00 | 10.00 | 52.49 | 1.00 | 0.17 | 37.70 | 5.40 | 14.01 | 156.00 | 0.87 |
| KDF1 | 80640.00 | 0.84 | 9.00 | 9.00 | 51.47 | 1.00 | 0.17 | 36.95 | 5.38 | 0.60 | 8.00 | 0.97 |
| MAZ | 14.00 | 0.39 | 5.00 | 5.00 | 40.83 | 1.00 | 0.17 | 31.25 | 4.95 | 19.37 | 82.00 | 0.60 |
| IRF2BP1 | 7.00 | 0.28 | 4.00 | 5.00 | 38.73 | 1.00 | 0.17 | 32.22 | 5.07 | 95.86 | 266.00 | 0.30 |
| RAB40B | 7.00 | 0.28 | 4.00 | 5.00 | 38.29 | 1.00 | 0.17 | 32.17 | 5.02 | 73.93 | 264.00 | 0.30 |
| RAB3D | 3.00 | 0.31 | 2.00 | 3.00 | 34.42 | 1.00 | 0.17 | 30.73 | 4.93 | 5.53 | 36.00 | 0.33 |
| RGL3 | 3.00 | 0.31 | 2.00 | 3.00 | 33.08 | 1.00 | 0.17 | 31.30 | 5.01 | 45.90 | 186.00 | 0.33 |
| VPS52 | 2.00 | 0.00 | 1.00 | 2.00 | 24.81 | 1.00 | 0.17 | 29.50 | 4.88 | 25.78 | 74.00 | 0.00 |
| RCCD1 | 2.00 | 0.00 | 1.00 | 2.00 | 24.78 | 1.00 | 0.17 | 28.30 | 4.80 | 8.99 | 34.00 | 0.00 |
| MRPS26 | 2.00 | 0.00 | 1.00 | 2.00 | 24.75 | 1.00 | 0.17 | 26.10 | 4.42 | 3.40 | 18.00 | 0.00 |
| PIR | 2.00 | 0.00 | 1.00 | 2.00 | 23.02 | 1.00 | 0.17 | 26.33 | 4.51 | 16.20 | 36.00 | 0.00 |
| CHTOP | 2.00 | 0.31 | 2.00 | 2.00 | 18.95 | 1.00 | 0.17 | 25.67 | 4.40 | 0.00 | 0.00 | 1.00 |
| ANP32A | 2.00 | 0.31 | 2.00 | 2.00 | 17.96 | 1.00 | 0.17 | 25.67 | 4.40 | 0.00 | 0.00 | 1.00 |
| RTKN | 1.00 | 0.00 | 1.00 | 1.00 | 17.47 | 1.00 | 0.17 | 26.98 | 4.60 | 0.00 | 0.00 | 0.00 |
| GKAP1 | 1.00 | 0.00 | 1.00 | 1.00 | 15.47 | 1.00 | 0.17 | 25.57 | 4.47 | 0.00 | 0.00 | 0.00 |
| BEND3 | 1.00 | 0.00 | 1.00 | 1.00 | 12.81 | 1.00 | 0.17 | 23.77 | 4.20 | 0.00 | 0.00 | 0.00 |
| AARSD1 | 1.00 | 0.00 | 1.00 | 1.00 | 10.50 | 1.00 | 0.17 | 21.68 | 3.85 | 0.00 | 0.00 | 0.00 |
| NR2F6 | 1.00 | 0.00 | 1.00 | 1.00 | 10.05 | 1.00 | 0.17 | 23.22 | 4.14 | 0.00 | 0.00 | 0.00 |
| HOOK1 | 4.00 | 0.31 | 3.00 | 3.00 | 35.90 | 1.00 | 0.14 | 30.63 | 4.95 | 5.00 | 62.00 | 0.67 |
| SETD6 | 2.00 | 0.31 | 2.00 | 2.00 | 22.66 | 1.00 | 0.14 | 26.89 | 4.56 | 0.00 | 0.00 | 1.00 |
| BLOC1S4 | 1.00 | 0.00 | 1.00 | 1.00 | 17.80 | 1.00 | 0.14 | 26.79 | 4.55 | 0.00 | 0.00 | 0.00 |
| ZSCAN16 | 1.00 | 0.00 | 1.00 | 1.00 | 17.34 | 1.00 | 0.14 | 25.84 | 4.48 | 0.00 | 0.00 | 0.00 |
| SLC29A2 | 1.00 | 0.00 | 1.00 | 1.00 | 17.03 | 1.00 | 0.14 | 25.84 | 4.41 | 0.00 | 0.00 | 0.00 |
| C1orf35 | 1.00 | 0.00 | 1.00 | 1.00 | 16.54 | 1.00 | 0.14 | 25.49 | 4.42 | 0.00 | 0.00 | 0.00 |
| CLCN2 | 1.00 | 0.00 | 1.00 | 1.00 | 16.39 | 1.00 | 0.14 | 25.49 | 4.42 | 0.00 | 0.00 | 0.00 |
| C2CD4D | 1.00 | 0.00 | 1.00 | 1.00 | 12.48 | 1.00 | 0.14 | 22.89 | 3.99 | 0.00 | 0.00 | 0.00 |
| PGAP2 | 1.00 | 0.00 | 1.00 | 1.00 | 4.21 | 1.00 | 0.14 | 17.84 | 2.89 | 0.00 | 0.00 | 0.00 |
