# Supplementary material for: TRIM28 is a distinct prognostic biomarker that worsens the tumor immune microenvironment in lung adenocarcinoma
Source: Aging (Albany NY). 2020 Oct 22;12(20):20308–31. doi: 10.18632/aging.103804 (PMC7655206; doi:10.18632/aging.103804)
Supplement: Supplementary Table 4 [file aging-12-103804-s005..docx]

Supplementary Table 4. The substrates were predicted by using UbiBrowser.

| E3 | E3GENE | SUB | SUBGENE | HOMO | PFAM | GO | NET | MOTIF | SCORE |
| --- | --- | --- | --- | --- | --- | --- | --- | --- | --- |
| Q13263 | TRIM28 | P29590 | PML | 1 | 3.17 | 2.33 | 1.7 | 1 | 0.728 |
| Q13263 | TRIM28 | P22674 | CCNO | 1 | 3.17 | 2.33 | 1.29 | 1 | 0.727 |
| Q13263 | TRIM28 | P14373 | TRIM27 | 1 | 3.17 | 1.27 | 1.84 | 1 | 0.705 |
| Q13263 | TRIM28 | Q16512 | PKN1 | 1 | 1.73 | 2.33 | 2.39 | 1 | 0.705 |
| Q13263 | TRIM28 | P20248 | CCNA2 | 1 | 3.17 | 1.25 | 1.84 | 1 | 0.703 |
| Q13263 | TRIM28 | O60563 | CCNT1 | 1 | 3.17 | 1.25 | 2.39 | 1 | 0.703 |
| Q13263 | TRIM28 | Q00839 | HNRNPU | 1 | 3.17 | 1.24 | 2.17 | 1 | 0.703 |
| Q13263 | TRIM28 | Q9H3D4 | TP63 | 1 | 3.18 | 1.24 | 1.84 | 1 | 0.703 |
| Q13263 | TRIM28 | P14635 | CCNB1 | 1 | 3.17 | 1.25 | 1.84 | 1 | 0.703 |
| Q13263 | TRIM28 | P30281 | CCND3 | 1 | 3.17 | 1.25 | 1.84 | 1 | 0.703 |
| Q13263 | TRIM28 | P16885 | PLCG2 | 1 | 1.73 | 2.33 | 1.77 | 1 | 0.701 |
| Q13263 | TRIM28 | P30279 | CCND2 | 1 | 3.17 | 1.25 | 1.77 | 1 | 0.7 |
| Q13263 | TRIM28 | Q15306 | IRF4 | 1 | 3.17 | 1.25 | 1.77 | 1 | 0.7 |
| Q13263 | TRIM28 | O96020 | CCNE2 | 1 | 3.17 | 1.25 | 1.77 | 1 | 0.7 |
| Q13263 | TRIM28 | Q15542 | TAF5 | 1 | 1 | 3.77 | 1.84 | 1 | 0.699 |
| Q13263 | TRIM28 | P49848 | TAF6 | 1 | 1 | 3.77 | 1.84 | 1 | 0.699 |
| Q13263 | TRIM28 | O60869 | EDF1 | 1 | 1 | 3.77 | 1.84 | 1 | 0.699 |
| Q13263 | TRIM28 | Q16514 | TAF12 | 1 | 1 | 3.77 | 1.84 | 1 | 0.699 |
| Q13263 | TRIM28 | Q9BUJ2 | HNRNPUL1 | 1 | 3.17 | 1.24 | 1.77 | 1 | 0.699 |
| Q13263 | TRIM28 | O95361 | TRIM16 | 1 | 3.17 | 1.51 | 1.87 | 1 | 0.698 |
| Q13263 | TRIM28 | P24864 | CCNE1 | 1 | 3.17 | 1.27 | 1.69 | 1 | 0.697 |
| Q13263 | TRIM28 | Q7Z7C8 | TAF8 | 1 | 1 | 3.77 | 1.77 | 1 | 0.695 |
| Q13263 | TRIM28 | Q15543 | TAF13 | 1 | 1 | 3.77 | 1.77 | 1 | 0.695 |
| Q13263 | TRIM28 | P51946 | CCNH | 1 | 3.17 | 1.25 | 2.2 | 1 | 0.695 |
| Q13263 | TRIM28 | Q6P1X5 | TAF2 | 1 | 1 | 3.77 | 1.77 | 1 | 0.695 |
| Q13263 | TRIM28 | Q14653 | IRF3 | 1 | 3.17 | 1.25 | 1.69 | 1 | 0.695 |
| Q13263 | TRIM28 | P14316 | IRF2 | 1 | 3.17 | 1.13 | 1.84 | 1 | 0.694 |
| Q13263 | TRIM28 | Q92985 | IRF7 | 1 | 3.17 | 1.13 | 1.84 | 1 | 0.694 |
| Q13263 | TRIM28 | P10914 | IRF1 | 1 | 3.17 | 1.13 | 1.84 | 1 | 0.694 |
| Q13263 | TRIM28 | Q96S59 | RANBP9 | 1 | 3.17 | 1.13 | 1.84 | 1 | 0.694 |
| Q13263 | TRIM28 | Q14134 | TRIM29 | 1 | 3.17 | 1.13 | 2.39 | 1 | 0.694 |
| Q13263 | TRIM28 | Q9UPN9 | TRIM33 | 1 | 3.17 | 1.13 | 2.39 | 1 | 0.694 |
| Q13263 | TRIM28 | P78396 | CCNA1 | 1 | 3.17 | 1.13 | 1.84 | 1 | 0.694 |
| Q13263 | TRIM28 | O00268 | TAF4 | 1 | 1 | 3.77 | 1.99 | 1 | 0.691 |
| Q13263 | TRIM28 | Q15545 | TAF7 | 1 | 1 | 3.77 | 2.2 | 1 | 0.691 |
| Q13263 | TRIM28 | Q12962 | TAF10 | 1 | 1 | 3.77 | 2.2 | 1 | 0.691 |
| Q13263 | TRIM28 | Q15544 | TAF11 | 1 | 1 | 3.77 | 2.2 | 1 | 0.691 |
| Q13263 | TRIM28 | Q969Q1 | TRIM63 | 1 | 3.17 | 1.13 | 1.77 | 1 | 0.69 |
| Q13263 | TRIM28 | P21817 | RYR1 | 1 | 3.17 | 1.13 | 1.77 | 1 | 0.69 |
| Q13263 | TRIM28 | Q8IWR1 | TRIM59 | 1 | 3.17 | 1.13 | 2.3 | 1 | 0.69 |
| Q13263 | TRIM28 | Q13263 | TRIM28 | 1 | 3.17 | 1.27 | 2.06 | 1 | 0.69 |
| Q13263 | TRIM28 | Q8TDN4 | CABLES1 | 1 | 3.17 | 1.13 | 1.77 | 1 | 0.69 |
| Q13263 | TRIM28 | O94972 | TRIM37 | 1 | 3.17 | 1.13 | 1.77 | 1 | 0.69 |
| Q13263 | TRIM28 | Q92499 | DDX1 | 1 | 3.17 | 1.13 | 1.77 | 1 | 0.69 |
| Q13263 | TRIM28 | O43763 | TLX2 | 1 | 1.73 | 2.33 | 1.44 | 1 | 0.682 |
| Q13263 | TRIM28 | Q07687 | DLX2 | 1 | 1.73 | 2.33 | 1.44 | 1 | 0.682 |
| Q13263 | TRIM28 | Q92736 | RYR2 | 1 | 3.17 | 1.25 | 1.44 | 1 | 0.681 |
| Q13263 | TRIM28 | P24385 | CCND1 | 1 | 3.17 | 1.25 | 1.87 | 1 | 0.681 |
| Q13263 | TRIM28 | Q9UBL3 | ASH2L | 1 | 3.17 | 1.25 | 1.44 | 1 | 0.681 |
| Q13263 | TRIM28 | O60583 | CCNT2 | 1 | 3.17 | 1.25 | 1.44 | 1 | 0.681 |
| Q13263 | TRIM28 | Q86WT6 | TRIM69 | 1 | 3.17 | 1.25 | 1.44 | 1 | 0.681 |
| Q13263 | TRIM28 | O15350 | TP73 | 1 | 3.18 | 1.24 | 1.87 | 1 | 0.68 |
| Q13263 | TRIM28 | P51959 | CCNG1 | 1 | 3.17 | 1 | 1.77 | 1 | 0.679 |
| Q13263 | TRIM28 | O15164 | TRIM24 | 1 | 3.17 | 1.13 | 2.06 | 1 | 0.679 |
| Q13263 | TRIM28 | P21675 | TAF1 | 1 | 1 | 3.77 | 1.44 | 1 | 0.676 |
| Q13263 | TRIM28 | Q9HBM6 | TAF9B | 1 | 1 | 3.77 | 1.44 | 1 | 0.676 |
| Q13263 | TRIM28 | Q92750 | TAF4B | 1 | 1 | 3.77 | 1.44 | 1 | 0.676 |
| Q13263 | TRIM28 | Q8IZX4 | TAF1L | 1 | 1 | 3.77 | 1.44 | 1 | 0.676 |
| Q13263 | TRIM28 | P20226 | TBP | 1 | 1 | 3.77 | 1.7 | 1 | 0.676 |
| Q13263 | TRIM28 | Q5H9L4 | TAF7L | 1 | 1 | 3.77 | 1.44 | 1 | 0.676 |
| Q13263 | TRIM28 | P35638 | DDIT3 | 1 | 1 | 2.93 | 2.39 | 1 | 0.675 |
| Q13263 | TRIM28 | P52701 | MSH6 | 1 | 1 | 2.93 | 1.84 | 1 | 0.675 |
| Q13263 | TRIM28 | P11021 | HSPA5 | 1 | 1 | 2.93 | 1.84 | 1 | 0.675 |
| Q13263 | TRIM28 | P52333 | JAK3 | 1 | 1 | 2.88 | 1.84 | 1 | 0.674 |
| Q13263 | TRIM28 | P10721 | KIT | 1 | 1 | 2.88 | 1.84 | 1 | 0.674 |
| Q13263 | TRIM28 | Q8TAU0 | NKX2-3 | 1 | 1.73 | 2.33 | 1.29 | 1 | 0.672 |
| Q13263 | TRIM28 | P05023 | ATP1A1 | 1 | 1 | 2.93 | 1.77 | 1 | 0.671 |
| Q13263 | TRIM28 | P43246 | MSH2 | 1 | 1 | 2.93 | 1.77 | 1 | 0.671 |
| Q13263 | TRIM28 | O95067 | CCNB2 | 1 | 3.17 | 1.13 | 1.44 | 1 | 0.671 |
| Q13263 | TRIM28 | P36406 | TRIM23 | 1 | 3.17 | 1.13 | 1.44 | 1 | 0.671 |
| Q13263 | TRIM28 | O75909 | CCNK | 1 | 3.17 | 1.13 | 1.44 | 1 | 0.671 |
| Q13263 | TRIM28 | Q9NZS9 | BFAR | 1 | 3.17 | 1.13 | 1.44 | 1 | 0.671 |
| Q13263 | TRIM28 | Q02556 | IRF8 | 1 | 3.17 | 1.13 | 1.44 | 1 | 0.671 |
| Q13263 | TRIM28 | Q9BVG3 | TRIM62 | 1 | 3.17 | 1.13 | 1.44 | 1 | 0.671 |
| Q13263 | TRIM28 | P19474 | TRIM21 | 1 | 3.17 | 1.13 | 1.44 | 1 | 0.671 |
| Q13263 | TRIM28 | Q9BTV7 | CABLES2 | 1 | 3.17 | 1.13 | 1.44 | 1 | 0.671 |
| Q13263 | TRIM28 | Q96S94 | CCNL2 | 1 | 3.17 | 1.25 | 1.29 | 1 | 0.67 |
| Q13263 | TRIM28 | P29597 | TYK2 | 1 | 1 | 2.88 | 1.77 | 1 | 0.67 |
| Q13263 | TRIM28 | Q96EB6 | SIRT1 | 1 | 1 | 2.93 | 1.69 | 1 | 0.667 |
| Q13263 | TRIM28 | P07948 | LYN | 1 | 1 | 2.88 | 1.69 | 1 | 0.665 |
| Q13263 | TRIM28 | P62380 | TBPL1 | 1 | 1 | 3.77 | 1.29 | 1 | 0.665 |
| Q13263 | TRIM28 | Q5VWG9 | TAF3 | 1 | 1 | 3.77 | 1.29 | 1 | 0.665 |
| Q13263 | TRIM28 | O60674 | JAK2 | 1 | 1 | 2.88 | 1.69 | 1 | 0.665 |
| Q13263 | TRIM28 | P43405 | SYK | 1 | 1 | 2.88 | 1.69 | 1 | 0.665 |
| Q13263 | TRIM28 | P06213 | INSR | 1 | 1 | 2.88 | 1.69 | 1 | 0.665 |
| Q13263 | TRIM28 | P54132 | BLM | 1 | 1 | 2.88 | 2.2 | 1 | 0.665 |
| Q13263 | TRIM28 | P23458 | JAK1 | 1 | 1 | 2.88 | 1.69 | 1 | 0.665 |
| Q13263 | TRIM28 | Q9BZR9 | TRIM8 | 1 | 3.17 | 1.51 | 1 | 1 | 0.664 |
| Q13263 | TRIM28 | Q14258 | TRIM25 | 1 | 3.17 | 1.13 | 1.29 | 1 | 0.66 |
| Q13263 | TRIM28 | Q5XPI4 | RNF123 | 1 | 3.17 | 1.13 | 1.29 | 1 | 0.66 |
| Q13263 | TRIM28 | Q9C035 | TRIM5 | 1 | 3.17 | 1.13 | 1.29 | 1 | 0.66 |
| Q13263 | TRIM28 | Q5W0U4 | BSPRY | 1 | 3.17 | 1.13 | 1.29 | 1 | 0.66 |
| Q13263 | TRIM28 | Q9UJV3 | MID2 | 1 | 3.17 | 1.13 | 1.29 | 1 | 0.66 |
| Q13263 | TRIM28 | Q96F44 | TRIM11 | 1 | 3.17 | 1.13 | 1.29 | 1 | 0.66 |
| Q13263 | TRIM28 | Q15751 | HERC1 | 1 | 3.17 | 1 | 1.44 | 1 | 0.659 |
| Q13263 | TRIM28 | Q14191 | WRN | 1 | 1 | 2.33 | 1.84 | 1 | 0.653 |
| Q13263 | TRIM28 | P29372 | MPG | 1 | 1 | 2.33 | 2.39 | 1 | 0.653 |
| Q13263 | TRIM28 | Q9Y3Q8 | TSC22D4 | 1 | 1 | 2.33 | 1.84 | 1 | 0.653 |
| Q13263 | TRIM28 | Q16666 | IFI16 | 1 | 1 | 2.33 | 1.84 | 1 | 0.653 |
| Q13263 | TRIM28 | P49841 | GSK3B | 1 | 1 | 2.93 | 1.44 | 1 | 0.651 |
| Q13263 | TRIM28 | Q9H093 | NUAK2 | 1 | 1 | 2.93 | 1.44 | 1 | 0.651 |
| Q13263 | TRIM28 | Q9NZJ5 | EIF2AK3 | 1 | 1 | 2.93 | 1.44 | 1 | 0.651 |
| Q13263 | TRIM28 | O60356 | NUPR1 | 1 | 1 | 2.93 | 1.44 | 1 | 0.651 |
| Q13263 | TRIM28 | Q9UM73 | ALK | 1 | 1 | 2.88 | 1.44 | 1 | 0.65 |
| Q13263 | TRIM28 | P04626 | ERBB2 | 1 | 1 | 2.88 | 1.87 | 1 | 0.65 |
| Q13263 | TRIM28 | Q06418 | TYRO3 | 1 | 1 | 2.88 | 1.44 | 1 | 0.65 |
| Q13263 | TRIM28 | P46063 | RECQL | 1 | 1 | 2.88 | 1.44 | 1 | 0.65 |
| Q13263 | TRIM28 | P46934 | NEDD4 | 1 | 1 | 2.33 | 1.77 | 1 | 0.649 |
| Q13263 | TRIM28 | P00813 | ADA | 1 | 1 | 2.33 | 2.3 | 1 | 0.649 |
| Q13263 | TRIM28 | Q15262 | PTPRK | 1 | 1 | 2.33 | 1.77 | 1 | 0.649 |
| Q13263 | TRIM28 | Q03468 | ERCC6 | 1 | 1 | 2.33 | 1.77 | 1 | 0.649 |
| Q13263 | TRIM28 | Q92630 | DYRK2 | 1 | 1 | 2.33 | 1.77 | 1 | 0.649 |
| Q13263 | TRIM28 | Q9HBA0 | TRPV4 | 1 | 1 | 2.33 | 1.77 | 1 | 0.649 |
| Q13263 | TRIM28 | P23560 | BDNF | 1 | 1 | 2.33 | 2.3 | 1 | 0.649 |
| Q13263 | TRIM28 | Q8WWL7 | CCNB3 | 1 | 3.17 | 1 | 1.29 | 1 | 0.648 |
| Q13263 | TRIM28 | P37275 | ZEB1 | 1 | 1.73 | 1.27 | 1.84 | 1 | 0.647 |
| Q13263 | TRIM28 | Q9C056 | NKX6-2 | 1 | 1.73 | 2.33 | 1 | 1 | 0.647 |
| Q13263 | TRIM28 | P78426 | NKX6-1 | 1 | 1.73 | 2.33 | 1 | 1 | 0.647 |
| Q13263 | TRIM28 | Q8IYM9 | TRIM22 | 1 | 3.17 | 1.27 | 1 | 1 | 0.647 |
| Q13263 | TRIM28 | Q5SQQ9 | VAX1 | 1 | 1.73 | 2.33 | 1 | 1 | 0.647 |
| Q13263 | TRIM28 | Q96PU5 | NEDD4L | 1 | 1.73 | 2.33 | 1 | 1 | 0.647 |
| Q13263 | TRIM28 | P28069 | POU1F1 | 1 | 1.73 | 1.27 | 2.39 | 1 | 0.647 |
| Q13263 | TRIM28 | Q9H0E2 | TOLLIP | 1 | 1.73 | 1.25 | 2.39 | 1 | 0.646 |
| Q13263 | TRIM28 | O43186 | CRX | 1 | 1.73 | 1.25 | 1.84 | 1 | 0.646 |
| Q13263 | TRIM28 | Q9HAU4 | SMURF2 | 1 | 1.73 | 1.25 | 1.84 | 1 | 0.646 |
| Q13263 | TRIM28 | Q15349 | RPS6KA2 | 1 | 1.73 | 1.25 | 1.84 | 1 | 0.646 |
| Q13263 | TRIM28 | P51587 | BRCA2 | 1 | 1 | 2.33 | 1.69 | 1 | 0.645 |
| Q13263 | TRIM28 | P27695 | APEX1 | 1 | 1 | 2.33 | 1.69 | 1 | 0.645 |
| Q13263 | TRIM28 | O75382 | TRIM3 | 1 | 3.17 | 1.25 | 1 | 1 | 0.645 |
| Q13263 | TRIM28 | O15016 | TRIM66 | 1 | 3.17 | 1.25 | 1 | 1 | 0.645 |
| Q13263 | TRIM28 | Q9UK58 | CCNL1 | 1 | 3.17 | 1.25 | 1 | 1 | 0.645 |
| Q13263 | TRIM28 | Q15699 | ALX1 | 1 | 1.73 | 1.27 | 1.77 | 1 | 0.643 |
| Q13263 | TRIM28 | P56915 | GSC | 1 | 1.73 | 1.25 | 1.77 | 1 | 0.642 |
| Q13263 | TRIM28 | P41743 | PRKCI | 1 | 1.73 | 1.25 | 1.77 | 1 | 0.642 |
| Q13263 | TRIM28 | P31270 | HOXA11 | 1 | 1.73 | 1.25 | 1.77 | 1 | 0.642 |
| Q13263 | TRIM28 | Q99697 | PITX2 | 1 | 1.73 | 1.25 | 1.77 | 1 | 0.642 |
| Q13263 | TRIM28 | P21579 | SYT1 | 1 | 1.73 | 1.25 | 2.3 | 1 | 0.642 |
| Q13263 | TRIM28 | P20265 | POU3F2 | 1 | 1.73 | 1.25 | 1.77 | 1 | 0.642 |
| Q13263 | TRIM28 | Q9UKI9 | POU2F3 | 1 | 1.73 | 1.25 | 1.77 | 1 | 0.642 |
| Q13263 | TRIM28 | Q86UR5 | RIMS1 | 1 | 1.73 | 1.25 | 1.77 | 1 | 0.642 |
| Q13263 | TRIM28 | Q6SJ96 | TBPL2 | 1 | 1 | 3.77 | 1 | 1 | 0.64 |
| Q13263 | TRIM28 | P01019 | AGT | 1 | 1 | 2.93 | 1.29 | 1 | 0.64 |
| Q13263 | TRIM28 | P51790 | CLCN3 | 1 | 1 | 2.93 | 1.29 | 1 | 0.64 |
| Q13263 | TRIM28 | P06576 | ATP5B | 1 | 1 | 2.93 | 1.29 | 1 | 0.64 |
| Q13263 | TRIM28 | P20585 | MSH3 | 1 | 1 | 2.93 | 1.29 | 1 | 0.64 |
| Q13263 | TRIM28 | Q9Y3D8 | AK6 | 1 | 1 | 3.77 | 1 | 1 | 0.64 |
| Q13263 | TRIM28 | Q04837 | SSBP1 | 1 | 1 | 2.93 | 1.29 | 1 | 0.64 |
| Q13263 | TRIM28 | Q99626 | CDX2 | 1 | 1.73 | 1.27 | 1.69 | 1 | 0.639 |
| Q13263 | TRIM28 | P43699 | NKX2-1 | 1 | 1.73 | 1.25 | 1.69 | 1 | 0.637 |
| Q13263 | TRIM28 | P09016 | HOXD4 | 1 | 1.73 | 1.13 | 1.84 | 1 | 0.636 |
| Q13263 | TRIM28 | P09086 | POU2F2 | 1 | 1.73 | 1.13 | 2.39 | 1 | 0.636 |
| Q13263 | TRIM28 | P47712 | PLA2G4A | 1 | 1.73 | 1.13 | 2.39 | 1 | 0.636 |
| Q13263 | TRIM28 | P49796 | RGS3 | 1 | 1.73 | 1.13 | 2.39 | 1 | 0.636 |
| Q13263 | TRIM28 | O75676 | RPS6KA4 | 1 | 1.73 | 1.13 | 1.84 | 1 | 0.636 |
| Q13263 | TRIM28 | Q9Y4K3 | TRAF6 | 1 | 1.73 | 1.13 | 1.84 | 1 | 0.636 |
| Q13263 | TRIM28 | Q13464 | ROCK1 | 1 | 1.73 | 1.13 | 2.39 | 1 | 0.636 |
| Q13263 | TRIM28 | Q04759 | PRKCQ | 1 | 1.73 | 1.13 | 1.84 | 1 | 0.636 |
| Q13263 | TRIM28 | Q02156 | PRKCE | 1 | 1.73 | 1.13 | 1.84 | 1 | 0.636 |
| Q13263 | TRIM28 | O00141 | SGK1 | 1 | 1.73 | 1.13 | 1.84 | 1 | 0.636 |
| Q13263 | TRIM28 | Q9UBC0 | ONECUT1 | 1 | 1.73 | 1.13 | 2.39 | 1 | 0.636 |
| Q13263 | TRIM28 | Q9HCE7 | SMURF1 | 1 | 1.73 | 1.13 | 1.84 | 1 | 0.636 |
| Q13263 | TRIM28 | P61371 | ISL1 | 1 | 1.73 | 1.13 | 2.39 | 1 | 0.636 |
| Q13263 | TRIM28 | Q13077 | TRAF1 | 1 | 1.73 | 1.13 | 1.84 | 1 | 0.636 |
| Q13263 | TRIM28 | P14653 | HOXB1 | 1 | 1.73 | 1.13 | 1.84 | 1 | 0.636 |
| Q13263 | TRIM28 | P23443 | RPS6KB1 | 1 | 1.73 | 1.13 | 1.84 | 1 | 0.636 |
| Q13263 | TRIM28 | P31269 | HOXA9 | 1 | 1.73 | 1.13 | 1.84 | 1 | 0.636 |
| Q13263 | TRIM28 | O14813 | PHOX2A | 1 | 1.73 | 1.13 | 1.84 | 1 | 0.636 |
| Q13263 | TRIM28 | P31260 | HOXA10 | 1 | 1.73 | 1.13 | 1.84 | 1 | 0.636 |
| Q13263 | TRIM28 | P31751 | AKT2 | 1 | 1.73 | 1.24 | 2.2 | 1 | 0.636 |
| Q13263 | TRIM28 | P17482 | HOXB9 | 1 | 1.73 | 1.13 | 1.84 | 1 | 0.636 |
| Q13263 | TRIM28 | P17509 | HOXB6 | 1 | 1.73 | 1.13 | 1.84 | 1 | 0.636 |
| Q13263 | TRIM28 | Q9C040 | TRIM2 | 1 | 3.17 | 1.13 | 1 | 1 | 0.635 |
| Q13263 | TRIM28 | Q6ZTA4 | TRIM67 | 1 | 3.17 | 1.13 | 1 | 1 | 0.635 |
| Q13263 | TRIM28 | Q9C029 | TRIM7 | 1 | 3.17 | 1.13 | 1 | 1 | 0.635 |
| Q13263 | TRIM28 | Q9NQ86 | TRIM36 | 1 | 3.17 | 1.13 | 1 | 1 | 0.635 |
| Q13263 | TRIM28 | Q9HCM9 | TRIM39 | 1 | 3.17 | 1.13 | 1 | 1 | 0.635 |
| Q13263 | TRIM28 | Q9C030 | TRIM6 | 1 | 3.17 | 1.13 | 1 | 1 | 0.635 |
| Q13263 | TRIM28 | Q6ZMU5 | TRIM72 | 1 | 3.17 | 1.13 | 1 | 1 | 0.635 |
| Q13263 | TRIM28 | Q9H2S5 | RNF39 | 1 | 3.17 | 1.13 | 1 | 1 | 0.635 |
| Q13263 | TRIM28 | Q9UPQ4 | TRIM35 | 1 | 3.17 | 1.13 | 1 | 1 | 0.635 |
| Q13263 | TRIM28 | Q86XT4 | TRIM50 | 1 | 3.17 | 1.13 | 1 | 1 | 0.635 |
| Q13263 | TRIM28 | Q9UDY6 | TRIM10 | 1 | 3.17 | 1.13 | 1 | 1 | 0.635 |
| Q13263 | TRIM28 | Q7Z4K8 | TRIM46 | 1 | 3.17 | 1.13 | 1 | 1 | 0.635 |
| Q13263 | TRIM28 | Q9Y577 | TRIM17 | 1 | 3.17 | 1.13 | 1 | 1 | 0.635 |
| Q13263 | TRIM28 | Q96BQ3 | TRIM43 | 1 | 3.17 | 1.13 | 1 | 1 | 0.635 |
| Q13263 | TRIM28 | Q86UV6 | TRIM74 | 1 | 3.17 | 1.13 | 1 | 1 | 0.635 |
| Q13263 | TRIM28 | Q13410 | BTN1A1 | 1 | 3.17 | 1.13 | 1 | 1 | 0.635 |
| Q13263 | TRIM28 | Q86UV7 | TRIM73 | 1 | 3.17 | 1.13 | 1 | 1 | 0.635 |
| Q13263 | TRIM28 | Q13568 | IRF5 | 1 | 3.17 | 1.13 | 1 | 1 | 0.635 |
| Q13263 | TRIM28 | Q6ZWI9 | RFPL4B | 1 | 3.17 | 1.13 | 1 | 1 | 0.635 |
| Q13263 | TRIM28 | Q6ZRF8 | RNF207 | 1 | 3.17 | 1.13 | 1 | 1 | 0.635 |
| Q13263 | TRIM28 | Q14142 | TRIM14 | 1 | 3.17 | 1.13 | 1 | 1 | 0.635 |
| Q13263 | TRIM28 | Q6VN20 | RANBP10 | 1 | 3.17 | 1.13 | 1 | 1 | 0.635 |
| Q13263 | TRIM28 | Q15413 | RYR3 | 1 | 3.17 | 1.13 | 1 | 1 | 0.635 |
| Q13263 | TRIM28 | Q8N3K9 | CMYA5 | 1 | 3.17 | 1.13 | 1 | 1 | 0.635 |
| Q13263 | TRIM28 | Q96LD4 | TRIM47 | 1 | 3.17 | 1.13 | 1 | 1 | 0.635 |
| Q13263 | TRIM28 | Q8N9V2 | TRIML1 | 1 | 3.17 | 1.13 | 1 | 1 | 0.635 |
| Q13263 | TRIM28 | Q96DX7 | TRIM44 | 1 | 3.17 | 1.13 | 1 | 1 | 0.635 |
| Q13263 | TRIM28 | Q8NG06 | TRIM58 | 1 | 3.17 | 1.13 | 1 | 1 | 0.635 |
| Q13263 | TRIM28 | Q96DX4 | RSPRY1 | 1 | 3.17 | 1.13 | 1 | 1 | 0.635 |
| Q13263 | TRIM28 | Q1KMD3 | HNRNPUL2 | 1 | 3.17 | 1.13 | 1 | 1 | 0.635 |
| Q13263 | TRIM28 | Q2Q1W2 | TRIM71 | 1 | 3.17 | 1.13 | 1 | 1 | 0.635 |
| Q13263 | TRIM28 | Q495X7 | TRIM60 | 1 | 3.17 | 1.13 | 1 | 1 | 0.635 |
| Q13263 | TRIM28 | Q5EBN2 | TRIM61 | 1 | 3.17 | 1.13 | 1 | 1 | 0.635 |
| Q13263 | TRIM28 | Q96A61 | TRIM52 | 1 | 3.17 | 1.13 | 1 | 1 | 0.635 |
| Q13263 | TRIM28 | Q8IWZ5 | TRIM42 | 1 | 3.17 | 1.13 | 1 | 1 | 0.635 |
| Q13263 | TRIM28 | Q8IWZ4 | TRIM48 | 1 | 3.17 | 1.13 | 1 | 1 | 0.635 |
| Q13263 | TRIM28 | Q6PJ69 | TRIM65 | 1 | 3.17 | 1.13 | 1 | 1 | 0.635 |
| Q13263 | TRIM28 | Q9C026 | TRIM9 | 1 | 3.17 | 1.13 | 1 | 1 | 0.635 |
| Q13263 | TRIM28 | Q9C019 | TRIM15 | 1 | 3.17 | 1.13 | 1 | 1 | 0.635 |
| Q13263 | TRIM28 | Q9BZY9 | TRIM31 | 1 | 3.17 | 1.13 | 1 | 1 | 0.635 |
| Q13263 | TRIM28 | Q9BYV6 | TRIM55 | 1 | 3.17 | 1.13 | 1 | 1 | 0.635 |
| Q13263 | TRIM28 | Q9H8W5 | TRIM45 | 1 | 3.17 | 1.13 | 1 | 1 | 0.635 |
| Q13263 | TRIM28 | Q9BYV2 | TRIM54 | 1 | 3.17 | 1.13 | 1 | 1 | 0.635 |
| Q13263 | TRIM28 | Q9BYJ4 | TRIM34 | 1 | 3.17 | 1.13 | 1 | 1 | 0.635 |
| Q13263 | TRIM28 | Q8IUD6 | RNF135 | 1 | 3.17 | 1.13 | 1 | 1 | 0.635 |
| Q13263 | TRIM28 | Q9BRZ2 | TRIM56 | 1 | 3.17 | 1.13 | 1 | 1 | 0.635 |
| Q13263 | TRIM28 | Q6AZZ1 | TRIM68 | 1 | 3.17 | 1.13 | 1 | 1 | 0.635 |
| Q13263 | TRIM28 | Q8WV44 | TRIM41 | 1 | 3.17 | 1.13 | 1 | 1 | 0.635 |
| Q13263 | TRIM28 | Q12899 | TRIM26 | 1 | 3.17 | 1.13 | 1 | 1 | 0.635 |
| Q13263 | TRIM28 | O00635 | TRIM38 | 1 | 3.17 | 1.13 | 1 | 1 | 0.635 |
| Q13263 | TRIM28 | P24863 | CCNC | 1 | 3.17 | 1.13 | 1 | 1 | 0.635 |
| Q13263 | TRIM28 | P41002 | CCNF | 1 | 3.17 | 1.13 | 1 | 1 | 0.635 |
| Q13263 | TRIM28 | O60858 | TRIM13 | 1 | 3.17 | 1.13 | 1 | 1 | 0.635 |
| Q13263 | TRIM28 | O15553 | MEFV | 1 | 3.17 | 1.13 | 1 | 1 | 0.635 |
| Q13263 | TRIM28 | Q00978 | IRF9 | 1 | 3.17 | 1.13 | 1 | 1 | 0.635 |
| Q13263 | TRIM28 | P0CI26 | TRIM49C | 1 | 3.17 | 1.13 | 1 | 1 | 0.635 |
| Q13263 | TRIM28 | O14896 | IRF6 | 1 | 3.17 | 1.13 | 1 | 1 | 0.635 |
| Q13263 | TRIM28 | O15344 | MID1 | 1 | 3.17 | 1.13 | 1 | 1 | 0.635 |
| Q13263 | TRIM28 | A8MT70 | ZBBX | 1 | 3.17 | 1.13 | 1 | 1 | 0.635 |
| Q13263 | TRIM28 | O75677 | RFPL1 | 1 | 3.17 | 1.13 | 1 | 1 | 0.635 |
| Q13263 | TRIM28 | A6NCK2 | TRIM43B | 1 | 3.17 | 1.13 | 1 | 1 | 0.635 |
| Q13263 | TRIM28 | O75679 | RFPL3 | 1 | 3.17 | 1.13 | 1 | 1 | 0.635 |
| Q13263 | TRIM28 | A6NGJ6 | TRIM64 | 1 | 3.17 | 1.13 | 1 | 1 | 0.635 |
| Q13263 | TRIM28 | A6NI03 | TRIM64B | 1 | 3.17 | 1.13 | 1 | 1 | 0.635 |
| Q13263 | TRIM28 | Q9C037 | TRIM4 | 1 | 3.17 | 1.13 | 1 | 1 | 0.635 |
| Q13263 | TRIM28 | A6NLU0 | RFPL4A | 1 | 3.17 | 1.13 | 1 | 1 | 0.635 |
| Q13263 | TRIM28 | O75678 | RFPL2 | 1 | 3.17 | 1.13 | 1 | 1 | 0.635 |
| Q13263 | TRIM28 | P0CI25 | TRIM49 | 1 | 3.17 | 1.13 | 1 | 1 | 0.635 |
| Q13263 | TRIM28 | P09629 | HOXB7 | 1 | 1.73 | 1.13 | 1.77 | 1 | 0.632 |
| Q13263 | TRIM28 | Q96J02 | ITCH | 1 | 1.73 | 1.13 | 1.77 | 1 | 0.632 |
| Q13263 | TRIM28 | Q93009 | USP7 | 1 | 1.73 | 1.13 | 1.77 | 1 | 0.632 |
| Q13263 | TRIM28 | P14651 | HOXB3 | 1 | 1.73 | 1.13 | 1.77 | 1 | 0.632 |
| Q13263 | TRIM28 | P14652 | HOXB2 | 1 | 1.73 | 1.13 | 1.77 | 1 | 0.632 |
| Q13263 | TRIM28 | P17483 | HOXB4 | 1 | 1.73 | 1.13 | 1.77 | 1 | 0.632 |
| Q13263 | TRIM28 | P31273 | HOXC8 | 1 | 1.73 | 1.13 | 1.77 | 1 | 0.632 |
| Q13263 | TRIM28 | P28358 | HOXD10 | 1 | 1.73 | 1.13 | 1.77 | 1 | 0.632 |
| Q13263 | TRIM28 | P35680 | HNF1B | 1 | 1.73 | 1.13 | 1.77 | 1 | 0.632 |
| Q13263 | TRIM28 | P35548 | MSX2 | 1 | 1.73 | 1.13 | 1.77 | 1 | 0.632 |
| Q13263 | TRIM28 | P05771 | PRKCB | 1 | 1.73 | 1.13 | 1.77 | 1 | 0.632 |
| Q13263 | TRIM28 | P52945 | PDX1 | 1 | 1.73 | 1.13 | 1.77 | 1 | 0.632 |
| Q13263 | TRIM28 | Q01860 | POU5F1 | 1 | 1.73 | 1.13 | 1.77 | 1 | 0.632 |
| Q13263 | TRIM28 | P40424 | PBX1 | 1 | 1.73 | 1.13 | 2.3 | 1 | 0.632 |
| Q13263 | TRIM28 | Q12837 | POU4F2 | 1 | 1.73 | 1.13 | 2.3 | 1 | 0.632 |
| Q13263 | TRIM28 | Q9NQ66 | PLCB1 | 1 | 1.73 | 1.13 | 1.77 | 1 | 0.632 |
| Q13263 | TRIM28 | Q01851 | POU4F1 | 1 | 1.73 | 1.13 | 2.3 | 1 | 0.632 |
| Q13263 | TRIM28 | O00750 | PIK3C2B | 1 | 1.73 | 1.13 | 1.77 | 1 | 0.632 |
| Q13263 | TRIM28 | Q13114 | TRAF3 | 1 | 1.73 | 1.13 | 1.77 | 1 | 0.632 |
| Q13263 | TRIM28 | Q9BUZ4 | TRAF4 | 1 | 1.73 | 1.13 | 1.77 | 1 | 0.632 |
| Q13263 | TRIM28 | Q03014 | HHEX | 1 | 1.73 | 1.13 | 1.77 | 1 | 0.632 |
| Q13263 | TRIM28 | P05362 | ICAM1 | 1 | 1 | 2.33 | 1.44 | 1 | 0.628 |
| Q13263 | TRIM28 | P20749 | BCL3 | 1 | 1 | 2.33 | 1.44 | 1 | 0.628 |
| Q13263 | TRIM28 | Q9UGN5 | PARP2 | 1 | 1 | 2.33 | 1.44 | 1 | 0.628 |
| Q13263 | TRIM28 | Q13569 | TDG | 1 | 1 | 2.33 | 1.87 | 1 | 0.628 |
| Q13263 | TRIM28 | P04216 | THY1 | 1 | 1 | 2.33 | 1.44 | 1 | 0.628 |
| Q13263 | TRIM28 | P29323 | EPHB2 | 1 | 1 | 2.33 | 1.44 | 1 | 0.628 |
| Q13263 | TRIM28 | P35462 | DRD3 | 1 | 1 | 2.33 | 1.44 | 1 | 0.628 |
| Q13263 | TRIM28 | Q969S2 | NEIL2 | 1 | 1 | 2.33 | 1.44 | 1 | 0.628 |
| Q13263 | TRIM28 | Q01201 | RELB | 1 | 1 | 2.33 | 1.44 | 1 | 0.628 |
| Q13263 | TRIM28 | P57727 | TMPRSS3 | 1 | 1 | 2.33 | 1.44 | 1 | 0.628 |
| Q13263 | TRIM28 | P01137 | TGFB1 | 1 | 1 | 2.33 | 1.44 | 1 | 0.628 |
| Q13263 | TRIM28 | Q04917 | YWHAH | 1 | 1 | 2.33 | 1.7 | 1 | 0.628 |
| Q13263 | TRIM28 | P28360 | MSX1 | 1 | 1.73 | 1.13 | 1.69 | 1 | 0.627 |
| Q13263 | TRIM28 | P20823 | HNF1A | 1 | 1.73 | 1.13 | 1.69 | 1 | 0.627 |
| Q13263 | TRIM28 | P19174 | PLCG1 | 1 | 1.73 | 1.13 | 1.69 | 1 | 0.627 |
| Q13263 | TRIM28 | P20936 | RASA1 | 1 | 1.73 | 1.13 | 1.69 | 1 | 0.627 |
| Q13263 | TRIM28 | P11274 | BCR | 1 | 1.73 | 1.13 | 1.69 | 1 | 0.627 |
| Q13263 | TRIM28 | P26367 | PAX6 | 1 | 1.73 | 1.13 | 1.69 | 1 | 0.627 |
| Q13263 | TRIM28 | Q01826 | SATB1 | 1 | 1.73 | 1.13 | 2.2 | 1 | 0.627 |
| Q13263 | TRIM28 | Q12933 | TRAF2 | 1 | 1.73 | 1.13 | 1.69 | 1 | 0.627 |
| Q13263 | TRIM28 | Q96A44 | SPSB4 | 1 | 3.17 | 1 | 1 | 1 | 0.623 |
| Q13263 | TRIM28 | Q9BSJ1 | TRIM51 | 1 | 3.17 | 1 | 1 | 1 | 0.623 |
| Q13263 | TRIM28 | Q9BTV5 | FSD1 | 1 | 3.17 | 1 | 1 | 1 | 0.623 |
| Q13263 | TRIM28 | Q9BXM9 | FSD1L | 1 | 3.17 | 1 | 1 | 1 | 0.623 |
| Q13263 | TRIM28 | Q8ND76 | CCNY | 1 | 3.17 | 1 | 1 | 1 | 0.623 |
| Q13263 | TRIM28 | Q8NCJ5 | SPRYD3 | 1 | 3.17 | 1 | 1 | 1 | 0.623 |
| Q13263 | TRIM28 | Q8N815 | CNTD1 | 1 | 3.17 | 1 | 1 | 1 | 0.623 |
| Q13263 | TRIM28 | Q8N7R7 | CCNYL1 | 1 | 3.17 | 1 | 1 | 1 | 0.623 |
| Q13263 | TRIM28 | Q8N7C3 | TRIML2 | 1 | 3.17 | 1 | 1 | 1 | 0.623 |
| Q13263 | TRIM28 | Q99619 | SPSB2 | 1 | 3.17 | 1 | 1 | 1 | 0.623 |
| Q13263 | TRIM28 | Q8WVV5 | BTN2A2 | 1 | 3.17 | 1 | 1 | 1 | 0.623 |
| Q13263 | TRIM28 | Q16589 | CCNG2 | 1 | 3.17 | 1 | 1 | 1 | 0.623 |
| Q13263 | TRIM28 | Q96BD6 | SPSB1 | 1 | 3.17 | 1 | 1 | 1 | 0.623 |
| Q13263 | TRIM28 | P0C7X3 | CCNYL3 | 1 | 3.17 | 1 | 1 | 1 | 0.623 |
| Q13263 | TRIM28 | P0C7Q3 | FAM58BP | 1 | 3.17 | 1 | 1 | 1 | 0.623 |
| Q13263 | TRIM28 | P0C2W1 | FBXO45 | 1 | 3.17 | 1 | 1 | 1 | 0.623 |
| Q13263 | TRIM28 | Q96KV6 | BTN2A3P | 1 | 3.17 | 1 | 1 | 1 | 0.623 |
| Q13263 | TRIM28 | Q96PL5 | ERMAP | 1 | 3.17 | 1 | 1 | 1 | 0.623 |
| Q13263 | TRIM28 | Q309B1 | TRIM16L | 1 | 3.17 | 1 | 1 | 1 | 0.623 |
| Q13263 | TRIM28 | Q3C1W6 |  | 1 | 3.17 | 1 | 1 | 1 | 0.623 |
| Q13263 | TRIM28 | Q8WW59 | SPRYD4 | 1 | 3.17 | 1 | 1 | 1 | 0.623 |
| Q13263 | TRIM28 | A1L4K1 | FSD2 | 1 | 3.17 | 1 | 1 | 1 | 0.623 |
| Q13263 | TRIM28 | Q8N1B3 | FAM58A | 1 | 3.17 | 1 | 1 | 1 | 0.623 |
| Q13263 | TRIM28 | Q9H8S5 | CNTD2 | 1 | 3.17 | 1 | 1 | 1 | 0.623 |
| Q13263 | TRIM28 | P39880 | CUX1 | 1 | 1.73 | 1 | 1.84 | 1 | 0.623 |
| Q13263 | TRIM28 | Q6PJ21 | SPSB3 | 1 | 3.17 | 1 | 1 | 1 | 0.623 |
| Q13263 | TRIM28 | Q7KYR7 | BTN2A1 | 1 | 3.17 | 1 | 1 | 1 | 0.623 |
| Q13263 | TRIM28 | A6NLI5 | TRIM64C | 1 | 3.17 | 1 | 1 | 1 | 0.623 |
| Q13263 | TRIM28 | A6NK02 | TRIM75P | 1 | 3.17 | 1 | 1 | 1 | 0.623 |
| Q13263 | TRIM28 | A6NDQ2 |  | 1 | 3.17 | 1 | 1 | 1 | 0.623 |
| Q13263 | TRIM28 | A6NDI0 | TRIM49B | 1 | 3.17 | 1 | 1 | 1 | 0.623 |
| Q13263 | TRIM28 | Q6ZMN8 | CCNI2 | 1 | 3.17 | 1 | 1 | 1 | 0.623 |
| Q13263 | TRIM28 | A6NCD1 |  | 1 | 3.17 | 1 | 1 | 1 | 0.623 |
| Q13263 | TRIM28 | Q6UXG8 | BTNL9 | 1 | 3.17 | 1 | 1 | 1 | 0.623 |
| Q13263 | TRIM28 | Q6UXE8 | BTNL3 | 1 | 3.17 | 1 | 1 | 1 | 0.623 |
| Q13263 | TRIM28 | Q5W111 | SPRYD7 | 1 | 3.17 | 1 | 1 | 1 | 0.623 |
| Q13263 | TRIM28 | Q6UX41 | BTNL8 | 1 | 3.17 | 1 | 1 | 1 | 0.623 |
| Q13263 | TRIM28 | O00481 | BTN3A1 | 1 | 3.17 | 1 | 1 | 1 | 0.623 |
| Q13263 | TRIM28 | Q5T5M9 | CCNJ | 1 | 3.17 | 1 | 1 | 1 | 0.623 |
| Q13263 | TRIM28 | O00478 | BTN3A3 | 1 | 3.17 | 1 | 1 | 1 | 0.623 |
| Q13263 | TRIM28 | Q8IV13 | CCNJL | 1 | 3.17 | 1 | 1 | 1 | 0.623 |
| Q13263 | TRIM28 | F8VTS6 | RFPL4AL1 | 1 | 3.17 | 1 | 1 | 1 | 0.623 |
| Q13263 | TRIM28 | Q9UBX0 | HESX1 | 1 | 1.73 | 1.27 | 1.44 | 1 | 0.623 |
| Q13263 | TRIM28 | C9J1S8 | TRIM49D2P | 1 | 3.17 | 1 | 1 | 1 | 0.623 |
| Q13263 | TRIM28 | Q14094 | CCNI | 1 | 3.17 | 1 | 1 | 1 | 0.623 |
| Q13263 | TRIM28 | Q15911 | ZFHX3 | 1 | 1.73 | 1.25 | 1.44 | 1 | 0.621 |
| Q13263 | TRIM28 | Q15418 | RPS6KA1 | 1 | 1.73 | 1.25 | 1.7 | 1 | 0.621 |
| Q13263 | TRIM28 | Q05513 | PRKCZ | 1 | 1.73 | 1.25 | 1.44 | 1 | 0.621 |
| Q13263 | TRIM28 | P51812 | RPS6KA3 | 1 | 1.73 | 1.25 | 1.87 | 1 | 0.621 |
| Q13263 | TRIM28 | O75582 | RPS6KA5 | 1 | 1.73 | 1.25 | 1.87 | 1 | 0.621 |
| Q13263 | TRIM28 | P31749 | AKT1 | 1 | 1.73 | 1.25 | 1.87 | 1 | 0.621 |
| Q13263 | TRIM28 | P40425 | PBX2 | 1 | 1.73 | 1.25 | 1.44 | 1 | 0.621 |
| Q13263 | TRIM28 | P35452 | HOXD12 | 1 | 1.73 | 1.25 | 1.44 | 1 | 0.621 |
| Q13263 | TRIM28 | P56178 | DLX5 | 1 | 1.73 | 1.24 | 1.44 | 1 | 0.62 |
| Q13263 | TRIM28 | P05129 | PRKCG | 1 | 1.73 | 1.24 | 1.44 | 1 | 0.62 |
| Q13263 | TRIM28 | Q9UIF7 | MUTYH | 1 | 1 | 2.33 | 1.29 | 1 | 0.617 |
| Q13263 | TRIM28 | Q9NQC3 | RTN4 | 1 | 1 | 2.33 | 1.29 | 1 | 0.617 |
| Q13263 | TRIM28 | P06746 | POLB | 1 | 1 | 2.33 | 1.29 | 1 | 0.617 |
| Q13263 | TRIM28 | Q96LA8 | PRMT6 | 1 | 1 | 2.33 | 1.29 | 1 | 0.617 |
| Q13263 | TRIM28 | P18075 | BMP7 | 1 | 1 | 2.33 | 1.29 | 1 | 0.617 |
| Q13263 | TRIM28 | P13051 | UNG | 1 | 1 | 2.33 | 1.29 | 1 | 0.617 |
| Q13263 | TRIM28 | O95243 | MBD4 | 1 | 1 | 2.33 | 1.29 | 1 | 0.617 |
| Q13263 | TRIM28 | P32249 | GPR183 | 1 | 1 | 2.33 | 1.29 | 1 | 0.617 |
| Q13263 | TRIM28 | Q12791 | KCNMA1 | 1 | 1 | 2.33 | 1.29 | 1 | 0.617 |
| Q13263 | TRIM28 | O15342 | ATP6V0E1 | 1 | 1 | 2.93 | 1 | 1 | 0.615 |
| Q13263 | TRIM28 | Q5SWA1 | PPP1R15B | 1 | 1 | 2.93 | 1 | 1 | 0.615 |
| Q13263 | TRIM28 | Q8NBQ7 | AQP11 | 1 | 1 | 2.93 | 1 | 1 | 0.615 |
| Q13263 | TRIM28 | O76024 | WFS1 | 1 | 1 | 2.93 | 1 | 1 | 0.615 |
| Q13263 | TRIM28 | Q9BQE4 | VIMP | 1 | 1 | 2.93 | 1 | 1 | 0.615 |
| Q13263 | TRIM28 | P04179 | SOD2 | 1 | 1 | 2.93 | 1 | 1 | 0.615 |
| Q13263 | TRIM28 | O75503 | CLN5 | 1 | 1 | 2.93 | 1 | 1 | 0.615 |
| Q13263 | TRIM28 | O15229 | KMO | 1 | 1 | 2.93 | 1 | 1 | 0.615 |
| Q13263 | TRIM28 | Q9NWW5 | CLN6 | 1 | 1 | 2.93 | 1 | 1 | 0.615 |
| Q13263 | TRIM28 | Q14149 | MORC3 | 1 | 1 | 2.93 | 1 | 1 | 0.615 |
| Q13263 | TRIM28 | P47901 | AVPR1B | 1 | 1 | 2.93 | 1 | 1 | 0.615 |
| Q13263 | TRIM28 | Q9UBV7 | B4GALT7 | 1 | 1 | 2.93 | 1 | 1 | 0.615 |
| Q13263 | TRIM28 | Q9NUX5 | POT1 | 1 | 1 | 2.93 | 1 | 1 | 0.615 |
| Q13263 | TRIM28 | Q9H0Y0 | ATG10 | 1 | 1 | 2.93 | 1 | 1 | 0.615 |
| Q13263 | TRIM28 | Q13733 | ATP1A4 | 1 | 1 | 2.93 | 1 | 1 | 0.615 |
| Q13263 | TRIM28 | P41181 | AQP2 | 1 | 1 | 2.93 | 1 | 1 | 0.615 |
| Q13263 | TRIM28 | Q9Y5S8 | NOX1 | 1 | 1 | 2.93 | 1 | 1 | 0.615 |
| Q13263 | TRIM28 | P50993 | ATP1A2 | 1 | 1 | 2.93 | 1 | 1 | 0.615 |
| Q13263 | TRIM28 | P13637 | ATP1A3 | 1 | 1 | 2.93 | 1 | 1 | 0.615 |
| Q13263 | TRIM28 | P08243 | ASNS | 1 | 1 | 2.93 | 1 | 1 | 0.615 |
| Q13263 | TRIM28 | Q13286 | CLN3 | 1 | 1 | 2.93 | 1 | 1 | 0.615 |
| Q13263 | TRIM28 | P13284 | IFI30 | 1 | 1 | 2.93 | 1 | 1 | 0.615 |
| Q13263 | TRIM28 | P30411 | BDKRB2 | 1 | 1 | 2.93 | 1 | 1 | 0.615 |
| Q13263 | TRIM28 | Q9UI12 | ATP6V1H | 1 | 1 | 2.93 | 1 | 1 | 0.615 |
| Q13263 | TRIM28 | P01375 | TNF | 1 | 1 | 2.93 | 1 | 1 | 0.615 |
| Q13263 | TRIM28 | Q8NHE4 | ATP6V0E2 | 1 | 1 | 2.93 | 1 | 1 | 0.615 |
| Q13263 | TRIM28 | P50897 | PPT1 | 1 | 1 | 2.93 | 1 | 1 | 0.615 |
| Q13263 | TRIM28 | O94761 | RECQL4 | 1 | 1 | 2.88 | 1 | 1 | 0.613 |
| Q13263 | TRIM28 | Q9Y6X8 | ZHX2 | 1 | 1.73 | 1.13 | 1.44 | 1 | 0.611 |
| Q13263 | TRIM28 | O00443 | PIK3C2A | 1 | 1.73 | 1.13 | 1.44 | 1 | 0.611 |
| Q13263 | TRIM28 | Q9Y2J0 | RPH3A | 1 | 1.73 | 1.13 | 1.44 | 1 | 0.611 |
| Q13263 | TRIM28 | Q05655 | PRKCD | 1 | 1.73 | 1.13 | 1.44 | 1 | 0.611 |
| Q13263 | TRIM28 | P32242 | OTX1 | 1 | 1.73 | 1.13 | 1.44 | 1 | 0.611 |
| Q13263 | TRIM28 | P56177 | DLX1 | 1 | 1.73 | 1.13 | 1.44 | 1 | 0.611 |
| Q13263 | TRIM28 | Q9UQ26 | RIMS2 | 1 | 1.73 | 1.13 | 1.44 | 1 | 0.611 |
| Q13263 | TRIM28 | Q05925 | EN1 | 1 | 1.73 | 1.13 | 1.44 | 1 | 0.611 |
| Q13263 | TRIM28 | P24723 | PRKCH | 1 | 1.73 | 1.13 | 1.44 | 1 | 0.611 |
| Q13263 | TRIM28 | Q9UKY1 | ZHX1 | 1 | 1.73 | 1.13 | 1.7 | 1 | 0.611 |
| Q13263 | TRIM28 | Q9UBR4 | LHX3 | 1 | 1.73 | 1.13 | 1.44 | 1 | 0.611 |
| Q13263 | TRIM28 | Q9NYD6 | HOXC10 | 1 | 1.73 | 1.13 | 1.44 | 1 | 0.611 |
| Q13263 | TRIM28 | O00463 | TRAF5 | 1 | 1.73 | 1.13 | 1.44 | 1 | 0.611 |
| Q13263 | TRIM28 | P49639 | HOXA1 | 1 | 1.73 | 1.13 | 1.44 | 1 | 0.611 |
| Q13263 | TRIM28 | P54821 | PRRX1 | 1 | 1.73 | 1.13 | 1.44 | 1 | 0.611 |
| Q13263 | TRIM28 | P48742 | LHX1 | 1 | 1.73 | 1.13 | 1.44 | 1 | 0.611 |
| Q13263 | TRIM28 | P47902 | CDX1 | 1 | 1.73 | 1.13 | 1.44 | 1 | 0.611 |
| Q13263 | TRIM28 | P17252 | PRKCA | 1 | 1.73 | 1.13 | 1.44 | 1 | 0.611 |
| Q13263 | TRIM28 | Q9BQY4 | RHOXF2 | 1 | 1.73 | 1.13 | 1.44 | 1 | 0.611 |
| Q13263 | TRIM28 | P09017 | HOXC4 | 1 | 1.73 | 1.13 | 1.44 | 1 | 0.611 |
| Q13263 | TRIM28 | Q96PV0 | SYNGAP1 | 1 | 1.73 | 1.13 | 1.44 | 1 | 0.611 |
| Q13263 | TRIM28 | P14859 | POU2F1 | 1 | 1.73 | 1.13 | 1.7 | 1 | 0.611 |
| Q13263 | TRIM28 | Q16513 | PKN2 | 1 | 1.73 | 1.13 | 1.44 | 1 | 0.611 |
| Q13263 | TRIM28 | P49335 | POU3F4 | 1 | 1.73 | 1.13 | 1.44 | 1 | 0.611 |
| Q13263 | TRIM28 | Q16820 | MEP1B | 1 | 1.73 | 1.13 | 1.44 | 1 | 0.611 |
| Q13263 | TRIM28 | P20719 | HOXA5 | 1 | 1.73 | 1.13 | 1.44 | 1 | 0.611 |
| Q13263 | TRIM28 | Q9H4I2 | ZHX3 | 1 | 1.73 | 1.13 | 1.44 | 1 | 0.611 |
| Q13263 | TRIM28 | P78337 | PITX1 | 1 | 1.73 | 1.13 | 1.44 | 1 | 0.611 |
| Q13263 | TRIM28 | Q8TE12 | LMX1A | 1 | 1.73 | 1.13 | 1.44 | 1 | 0.611 |
| Q13263 | TRIM28 | Q9H2B2 | SYT4 | 1 | 1.73 | 1.13 | 1.44 | 1 | 0.611 |
| Q13263 | TRIM28 | O43791 | SPOP | 1 | 1.73 | 1.13 | 1.44 | 1 | 0.611 |
| Q13263 | TRIM28 | Q9BPY8 | HOPX | 1 | 1.73 | 1.13 | 1.44 | 1 | 0.611 |
| Q13263 | TRIM28 | Q96C24 | SYTL4 | 1 | 1.73 | 1.25 | 1.29 | 1 | 0.61 |
| Q13263 | TRIM28 | Q03052 | POU3F1 | 1 | 1.73 | 1.25 | 1.29 | 1 | 0.61 |
| Q13263 | TRIM28 | O43581 | SYT7 | 1 | 1.73 | 1.25 | 1.29 | 1 | 0.61 |
| Q13263 | TRIM28 | Q15208 | STK38 | 1 | 1.73 | 1.25 | 1.29 | 1 | 0.61 |
| Q13263 | TRIM28 | P52952 | NKX2-5 | 1 | 1.73 | 1.25 | 1.29 | 1 | 0.61 |
| Q13263 | TRIM28 | Q9UMQ3 | BARX2 | 1 | 1.73 | 1.25 | 1.29 | 1 | 0.61 |
| Q13263 | TRIM28 | Q15475 | SIX1 | 1 | 1.73 | 1.25 | 1.29 | 1 | 0.61 |
| Q13263 | TRIM28 | P09884 | POLA1 | 1 | 1 | 1.51 | 1.84 | 1 | 0.609 |
| Q13263 | TRIM28 | O96017 | CHEK2 | 1 | 1 | 1.51 | 1.84 | 1 | 0.609 |
| Q13263 | TRIM28 | O75593 | FOXH1 | 1 | 1 | 1.51 | 1.84 | 1 | 0.609 |
| Q13263 | TRIM28 | P27708 | CAD | 1 | 1 | 1.51 | 1.84 | 1 | 0.609 |
| Q13263 | TRIM28 | P25490 | YY1 | 1 | 1 | 1.51 | 1.84 | 1 | 0.609 |
| Q13263 | TRIM28 | Q9UBW7 | ZMYM2 | 1 | 1 | 1.51 | 1.84 | 1 | 0.609 |
| Q13263 | TRIM28 | P27694 | RPA1 | 1 | 1 | 1.51 | 1.84 | 1 | 0.609 |
| Q13263 | TRIM28 | Q14980 | NUMA1 | 1 | 1 | 1.51 | 1.77 | 1 | 0.605 |
| Q13263 | TRIM28 | O60934 | NBN | 1 | 1 | 1.51 | 1.77 | 1 | 0.605 |
| Q13263 | TRIM28 | Q9ULX6 | AKAP8L | 1 | 1 | 1.51 | 1.77 | 1 | 0.605 |
| Q13263 | TRIM28 | Q9Y265 | RUVBL1 | 1 | 1 | 1.51 | 1.77 | 1 | 0.605 |
| Q13263 | TRIM28 | Q9Y230 | RUVBL2 | 1 | 1 | 1.51 | 1.77 | 1 | 0.605 |
| Q13263 | TRIM28 | Q99973 | TEP1 | 1 | 1 | 1.51 | 1.77 | 1 | 0.605 |
| Q13263 | TRIM28 | Q92547 | TOPBP1 | 1 | 1 | 1.51 | 1.77 | 1 | 0.605 |
| Q13263 | TRIM28 | O43823 | AKAP8 | 1 | 1 | 1.51 | 1.77 | 1 | 0.605 |
